# Predicting the Functions and Specificity of Triterpenoid Synthases: A Mechanism-Based Multi-intermediate Docking Approach

**DOI:** 10.1371/journal.pcbi.1003874

**Published:** 2014-10-09

**Authors:** Bo-Xue Tian, Frank H. Wallrapp, Gemma L. Holiday, Jeng-Yeong Chow, Patricia C. Babbitt, C. Dale Poulter, Matthew P. Jacobson

**Affiliations:** 1 Department of Pharmaceutical Chemistry, School of Pharmacy, University of California, San Francisco, San Francisco, California, United States of America; 2 California Institute for Quantitative Biomedical Research, University of California, San Francisco, San Francisco, California, United States of America; 3 Department of Bioengineering and Therapeutic Sciences, University of California, San Francisco, San Francisco, California, United States of America; 4 Department of Chemistry, University of Utah, Salt Lake City, Utah, United States of America; Icahn School of Medicine at Mount Sinai, United States of America

## Abstract

Terpenoid synthases construct the carbon skeletons of tens of thousands of natural products. To predict functions and specificity of triterpenoid synthases, a mechanism-based, multi-intermediate docking approach is proposed. In addition to enzyme function prediction, other potential applications of the current approach, such as enzyme mechanistic studies and enzyme redesign by mutagenesis, are discussed.

## Author Summary

The rapid growth in the number of protein sequences presents challenges for enzyme function assignment. Computational methods, such as bioinformatics, homology modeling and docking, are becoming increasingly important for predicting of enzyme functions from protein sequences. Terpenoids are one of largest classes of natural products, and many drugs (e.g. taxol) consist of terpenoids or terpenoid derivatives. Understanding the biosynthesis of the terpenoids is of great interest. Terpenoid synthases catalyze the key cyclization steps of the biosynthesis of terpenoids via carbocation rearrangements, generating numerous multiple-ring carbon skeletons. Triterpenoid synthases, as an important class of terpenoid synthases, catalyze the cyclization of either squalene or oxido-squalene into cyclized products such as sterols (e.g. lanosterol). In this work, we propose a computational approach that can be used to predict product specificity of the triterpenoid synthases. Our approach provides insight into the ‘design principles’ of these fascinating enzymes, and may become a practical approach for function prediction and enzyme engineering.

## Introduction

The terpenoids, also called isoprenoids, are one of the largest and most structurally diverse classes of natural products, and play vital roles in almost all life forms [1], [2]. In the biosynthesis of terpenoids, the isoprene units (C_5_) are assembled by polyprenyl transferases to give long chain terpenes such as geranyl diphosphate, farnesyl diphosphate, geranylgeranyl diphosphate, and squalene, which can then be converted into diverse carbon skeletons by the terpenoid synthases (TPSs) [3], [4]. Understanding the specificity of TPSs is of great significance to biochemistry, organic chemistry and medicinal chemistry.

**Figure 1:**
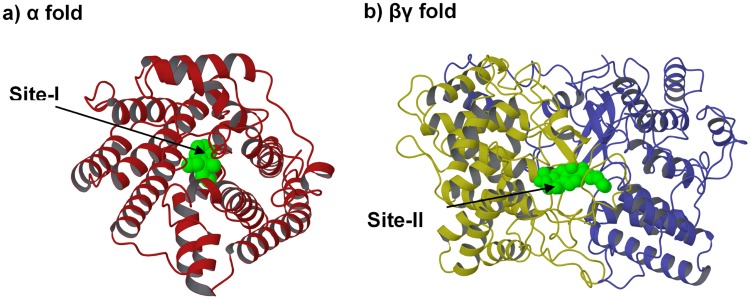
Example structures of TPSs: a) limonene synthase (PDB: 2ONH) [8]; b) squalene-hopene cyclase (PDB: 1SQC) [9], [10].

**Figure 2:**
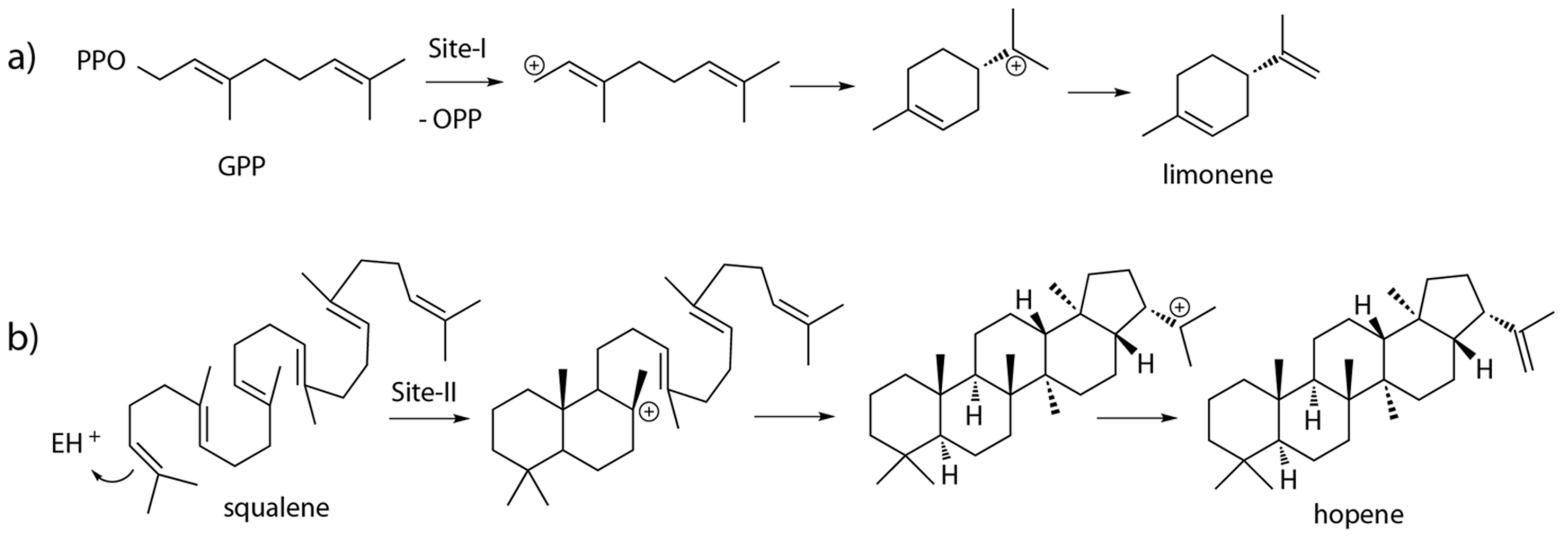
Example reactions of TPSs: a) limonene synthase; b) squalene-hopene cyclase.

According to the number of isoprene units (C_5_) of the substrates, TPSs can be classified into hemiterpenoid (C_5_), monoterpenoid (C_10_), sesquiterpenoid (C_15_), diterpenoid (C_20_), sesterterpenoid (C_25_), triterpenoid (C_30_) and sesquarterpenoid (C_35_) synthases. Most TPSs have one of two distinct protein folds [5]–[7], an α fold (class-I) and a βγ fold (class-II). For “class I” enzymes, the reaction is initiated by Mg^2+^-assisted removal of the diphosphate group, e.g., in limonene synthase [8] (Figure 1a and Figure 2a), while for “class II” enzymes, an acidic residue (normally Asp) initiates protonation of a double bond or an epoxy oxygen, e.g., in squalene-hopene cyclase [9], [10] (Figure 1b and Figure 2b). Both reaction types produce carbocation-olefin intermediates that undergo diverse cyclizations (rearrangements), followed by quenching of the carbocations via deprotonation or hydroxylation [5], [11], [12]. Some diterpenoid synthases that have the αβγ fusion fold can sequentially use both class I and II active sites to catalyze even more complicated reactions, e.g., the abietadiene synthase [13].

Some TPSs are promiscuous, e.g. the baruol synthase from *Arabidopsis thaliana* converts oxido-squalene into baruol (90%) as well as 22 other minor products [14]. Other TPSs are highly specific, e.g. the human lanosterol synthase generates only lanosterol, which has 7 chiral carbons [15]. Sometimes, even a single mutation in the TPSs can completely alter their product specificity, e.g. the H234S and H234T mutants of the lanosterol synthase from *Saccharomyces cerevisiae* produce 100% protosta-12,24-dien-3β-ol and 100% parkeol, respectively [16].

Crystal structures of TPSs [5], [6], [8]–[10], [13], [15], [17]–[30] provide a basis for understanding reaction mechanisms and specificity. As carbocations are short-lived, trapping the enzyme-bound intermediates is experimentally difficult. Therefore, high level quantum mechanics (QM) [31]–[35] and quantum mechanics/molecular mechanics (QM/MM) [36]–[40] calculations have been performed in order to understand the mechanisms of TPSs. Some *in silico* predicted catalytic mechanisms have been confirmed by experiments, e.g. a recent kinetic isotope effect (KIE) study on the mechanism of pentalenene synthase confirmed the QM-derived mechanism [41]. Hong *et al.* studied the catalytic mechanisms of a series of mono-, sesqui- and di-terpenoid synthases using QM methods, which have been summarized in a review article [32]. Based on QM/MM calculations, Rajamani *et al.* proposed that the product specificity of squalene-hopene cyclase is achieved by balancing thermodynamics and kinetic properties [39].

The aim of this and predecessor studies [42]–[53] is the development of robust methods for enzyme function prediction, using available sequence and structural information. In a recent work [50] involving a combination of bioinformatics, docking, homology modeling and enzymology, we have successfully predicted and experimentally validated the functions of 79 diverse members of the trans-polyprenyl transferase subgroup, which produces substrates for TPSs. Our long-term goal is essentially the same for the TPSs, i.e. building models to predict function of unknown enzymes [43]. However, due to the diversity of possible products, the TPSs present a more difficult problem than the polyprenyl transferases.

Both the polyprenyltransferases and the TPSs create challenges for purely sequence-based function prediction, because small sequence changes (including single point mutations) may result in a different product profile [16]. We thus believe, and have demonstrated for the polyprenyltransferases, that structure-based modeling approaches can provide important information about function. In the case of the polyprenyltransferases, product specificity is determined, to a large extent, by the depth of the cavity in which the growing polyisoprenoid chain binds. The situation for TPSs is considerably more complicated, in that the size and shape of the binding site, as well as the ability to differentially stabilize multiple carbocationic intermediates (and the transition states connecting them) all contribute to product specificity [54].

In principle, QM/MM methods [55]–[62] are ideal for studying these complex sequence-structure-function relationships, as has been demonstrated in focused studies of the mechanisms of certain TPS enzymes [37]–[40]. However, these methods are computationally too expensive to be used in large-scale function prediction of uncharacterized enzymes. Even for a single TPS, studying all known reaction channels by QM/MM is time consuming (to our knowledge, no such study has yet been reported). We hypothesize that molecular-mechanics-based “docking” methods, although they have a number of well-documented limitations, can nonetheless provide useful guidance concerning product specificity of TPS enzymes, with a throughput that is suitable for prospective investigations of large numbers of enzymes, as we have demonstrated for other classes of enzymes. The goal of our approach is not to eliminate experimental studies, which will be needed (for the foreseeable future) to test predictions, but rather to guide and focus the experimental studies. For TPS enzymes, long-term goals include the prediction of when/how changes in the binding sites impact specificity, and identification of TPS enzymes that may have novel activity (or conversely, guide the design of such enzymes).

We now describe a mechanism-based carbocation docking approach to predict function, and use the triterpenoid synthases [12], [63]–[66] (a subgroup of the class II TPS, proton initiated) to illustrate this approach. Triterpenoid synthases, which are found in a wide variety species including bacteria, archaea, plants, fungi, and animals, are involved in the biosynthesis of multicyclic metabolites such as sterols and saponins [64]. In this work, we dock against crystal structures and homology models for a wide variety of experimentally characterized triterpenoid synthases, in order to test the mechanism-based carbocation docking approach. Previous enzyme function prediction studies using intermediate docking [42], [44], [67], [68] have been conceptually simpler in that a single intermediate maps to one or a small number of possible substrates and products. In the case of TPSs, the number of possible substrates is small, but the number of potential products is enormous, and the generation of most products involves multiple carbocationic intermediates. Thus, instead of docking a single intermediate per reaction, we dock multiple intermediates along diverse reaction channels, in order to capture the mechanistic diversity (reaction channels) and product diversity of TPSs.

## Results

### Protein sequence similarity network of triterpenoid synthases

**Figure 3:**
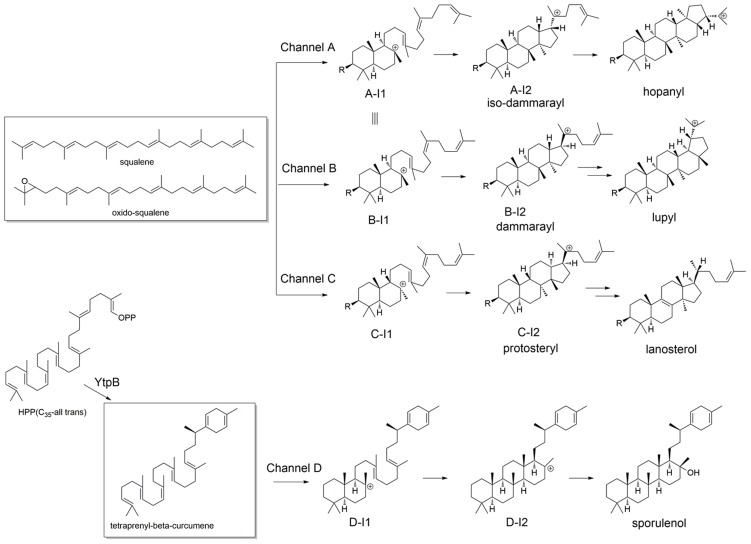
Reaction channels for triterpenoid synthase and triterpenoid synthase-like enzymes [54], [71].

Triterpenoid synthases (also called triterpene cyclases) catalyze the cyclization of squalene or oxido-squalene into hundreds of natural products [63], most of which are tetra- or pentacyclic structures such as lanosterol [15] and hopene [9], [10]. Triterpenoid synthases utilize one of three distinct reaction channels (Figure 3) [54]: 1) the hopene channel (Channel A); 2) the lupeol channel (Channel B); and the lanosterol channel (Channel C). In this work, we used the two known crystal structures for triterpenoid synthases, squalene-hopene cyclase from *Alicyclobacillus acidocaldarius* (PDB: 1SQC) [9], [10] and human lanosterol synthase (PDB: 1W6K) [15], for docking and building homology models, both of which are wild-type and have ligand bound (inhibitor for 1SQC and product for 1W6K).

**Figure 4:**
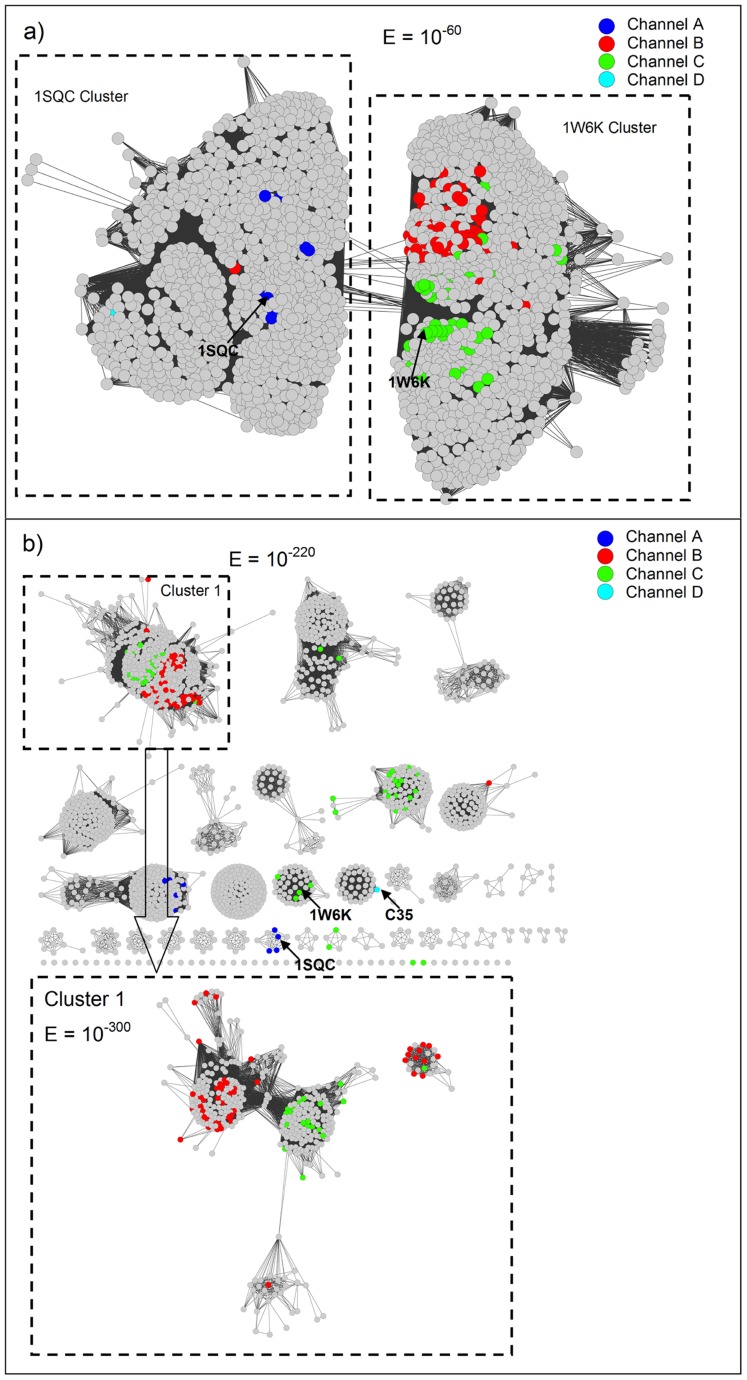
Sequence similarity network of triterpenoid synthase and triterpenoid synthase-like proteins colored by reaction channels. Each node represents a protein sequence, and nodes are connected when the Blast *E*-value for the pair of sequences is more significant than 10^−60^ (panel a) or 10^−220^/10^−300^ (panel b). Gray nodes represent enzymes lacking annotations in the manually curated portion of UniProtKB (Swiss-Prot), i.e., likely to be experimentally uncharacterized.

Figure 4 and Figure S1 show protein sequence similarity networks summarizing the known functions of the triterpenoid synthases, a bioinformatics tool that we have used extensively in the context of enzyme function prediction (for details of network generation, see Methods). Enzyme functions can be defined by the Enzyme Commission (EC) numbers, which describe the overall reaction being performed by an enzyme. The EC number consists of four levels, where the first three levels broadly describe the types of reaction being performed, and the fourth level generally describes the substrate specificity of the enzyme's overall chemical transformation. EC numbers and other related chemical information (e.g., reaction channels) can be mapped onto the sequence similarity networks (Figure 4 and Figure S1). To study enzyme functions with sequence similarity networks, different BLAST *E*-values [69] are scanned to gradually break the sequence similarity networks into smaller clusters until known enzyme functions are well segregated.

At an *E*-value of 1E^−60^ (an average sequence identity of 40%; obtained from the quartile plot see Figure S2), the sequences are separated into two major clusters, each of which contains the structure of one enzyme; for this reason, we label them as the 1SQC cluster and the 1W6K cluster (Figure 4a and Figure S1a). As the products of triterpenoid synthases are diverse, it is difficult to identify trends if we color the nodes according to EC numbers (Figure S1a). Even at an *E*-value of 1E^−220^ or 1E^−300^ (the average sequence identities are 50% and 70%, respectively; Figure S2), enzymes with different EC numbers still do not segregate well (Figure S1b), implying that it will be challenging to precisely predict function (full EC number) based on sequence alone. It is worth noting that the EC number generally only describes a single overall chemical transformation, thus is not well suited to categorizing promiscuous enzymes, which will catalyze several different EC numbers.

However, the products of triterpenoid synthases group into a few classes based on their carbon skeletons, which are related to the “reaction channels” (i.e. the series of carbocationic intermediates leading to various classes of products). Most of the reaction channels for the experimentally characterized enzymes can be separated at an *E*-value of 1E^−300^ in the sequence network (Figure 4), with only a few exceptions in cluster 1 (Figure 4b). Thus, functional relationships that are obscured by EC numbers, based on the exact products, are revealed by focusing instead on the nature of the carbocationic intermediates (and by implication the transition states connecting them) that are, presumably, differentially stabilized by the various classes of enzymes. It should be noted that in Figure 4, besides the three major reaction channels (Channel A–C) mentioned above, we also include a fourth Channel D (cyan; Figure 3), representing a recently discovered sesquarterpenoid (C_35_) synthase [70], [71]. As the crystal structure for this enzyme is not available and the sequence identity between this enzyme and 1SQC is low (∼25%), we cannot create a high quality model for this enzyme. In addition, the C_35_ intermediates corresponding to Channel D are predicted to bind poorly for most of our models (in comparison to the other three channels; Table S1), because the intermediates along Channel D are significantly different from those along Channel A–C in terms of size and shape [70], [71]. Hence, we do not consider Channel D further, and focus only on C_30_ carbocationic intermediates corresponding to Channels A–C.

### Hypotheses for docking

As classical molecular mechanics methods do not correctly describe transition states, docking transition states is impractical. Invoking assumptions similar to those in the “high-energy intermediate” approach of Shoichet and co-workers [67], we dock carbocationic intermediates. The primary difference is that, in this case, there is only one plausible substrate, but multiple possible intermediates that lead to different products. We hypothesized that by docking multiple intermediates (and ranking the results hierarchically), we could predict the dominant reaction channels for triterpenoid synthases, and then predict the likely product/precursor intermediates along the predicted reaction channel (rather than precise structures for the final products). At a minimum, we expected that we could at least exclude some implausible reaction channels, which have intermediates that are poorly stabilized by the enzyme, due to either steric clashes or electrostatic incompatibility. We do not dock every possible carbocation intermediate but only those that help distinguish the different reaction channels and product precursors.

### Docking to crystal structures of triterpenoid synthases

**Figure 5:**
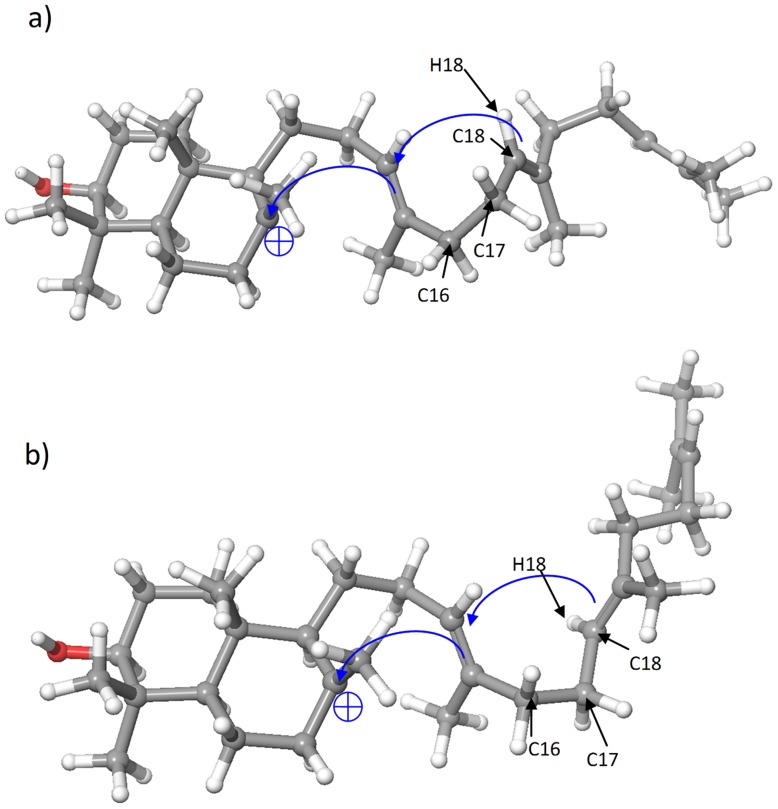
Illustration of the key dihedral angle C16-C17-C18-H18 that determines the conversion of I1 to I2: a) A-I1; b) B-I1.

We first discuss the docking results for the two crystal structures mentioned above, i.e., 1SQC and 1W6K, as an important test of the methodology. The key difference between the three major reaction channels (Channels A–C) is the stereochemistry of the 6,6-bicyclic and 6,6,6,5-tetracyclic carbocationic intermediates I1 and I2, respectively (Figure 3). It should be noted that A-I1 and B-I1 are chemically identical but are represented by different conformations which can convert to chemically different intermediates A-I2 and B-I2 (Figure 5). The rule of configuration transmission in triterpenoid synthases has been extensively discussed [54]; the key concept is that, with limited rotational freedom in the active site cavity, conformational differences in the upstream intermediates will be transferred to the downstream intermediates. As a practical matter, docking different conformations of the same intermediate (e.g. A-I1 and B-I1) results in different docking scores (see Methods for details), which we interpret in terms of the predicted reaction channel.

In order to take active site flexibility into account, an induced fit docking protocol is used for all docking calculations. Receptor flexibility is important to the current work because rearrangements of the carbocationic intermediates may slightly change the conformations of the active site residues. (In addition, when using homology models, as described below, receptor flexibility can compensate for small errors in the models.) To ensure the ligands are docked into a catalytically-relevant position, constraints were applied during the docking, which are essential for maintaining consistent poses of the carbocationic intermediates along the same reaction channel. Detailed procedures and parameters are provided in Methods.

According to previous QM/MM studies on squalene-hopene cyclase [39] and lanosterol synthase [38], there is only one transition state between I1 and I2, whose reaction barrier is significant (>10 kcal/mol). Therefore, we suggest that the transition state between I1 and I2 is a key specificity determinant for the three reaction channels defined above, and that stabilization of intermediates I1 and I2 can be used to distinguish reaction channels. However, we are aware that for some cases in which the binding affinities of the intermediates along different channels are very similar, this assumption may be insufficient.

**Figure 6:**
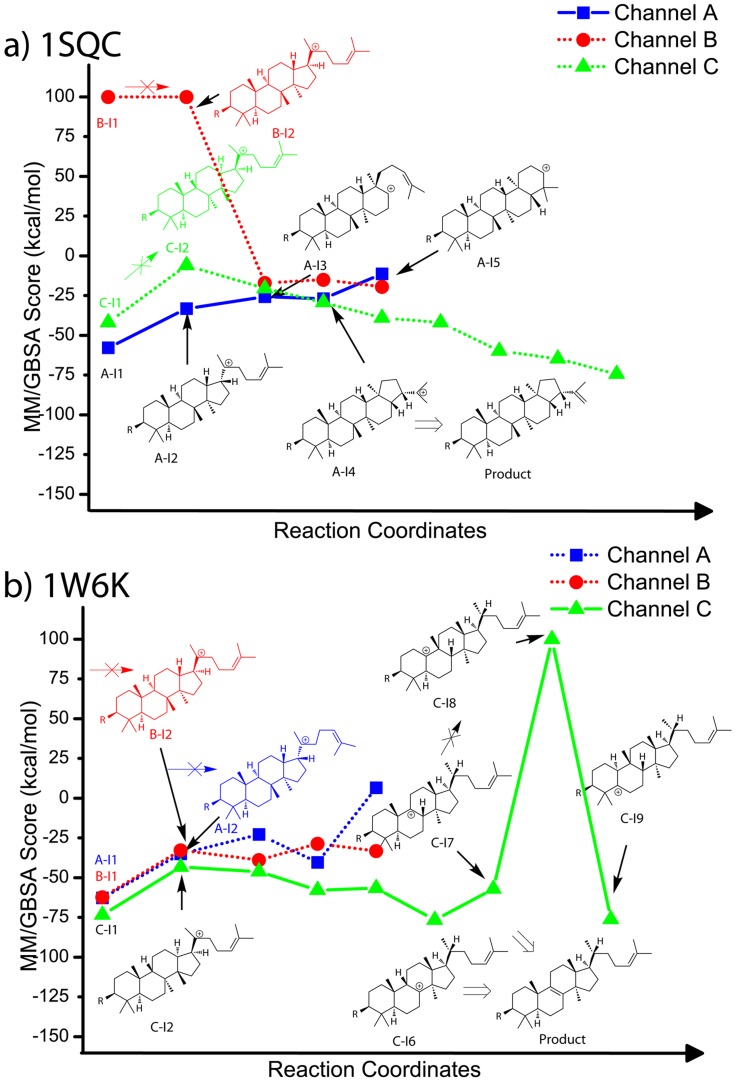
Carbocationic intermediate docking scores (MM/GBSA) along the reaction coordinates of a) 1SQC and b) 1W6K. We arbitrarily assigned a score of +100 kcal/mol to intermediates that could not be successfully docked.

Figure 6a shows the docking scores for intermediates along three major reaction channels docked to the squalene-hopene cyclase crystal structure (1SQC). Intermediates along reaction channel A (blue), which leads to the correct product hopene, clearly receive the most favorable docking scores. At present, we are unable to predict the specific products based simply on the docking scores. That is, the product precursor hopanyl cation (A-I4) is only the third best binder, implying that the current docking approach is not able to accurately predict the correct precursor cation of the major product; quantum mechanical methods may be necessary to achieve such a goal. However, as the TPSs are often promiscuous, carbocation docking can at least identify several possible intermediates that could lead to the final products, e.g. the second best binder A-I2 is the precursor of 6,6,6,5-tetracyclic byproducts of squalene-hopene cyclase. In a previous QM/MM study on 1SQC [39], the free energy barriers for the formation of the A-I2 and A-I4 intermediates were determined to be very similar (1.8 kcal/mol difference), but A-I4 is thermodynamically more stable (>10 kcal/mol difference). One possible way to improve the prediction results is to run further QM/MM calculations to evaluate the most likely intermediates from our docking hits, as well as transition states between the intermediates, but this approach is computationally expensive and beyond the scope of the current work.

**Figure 7:**
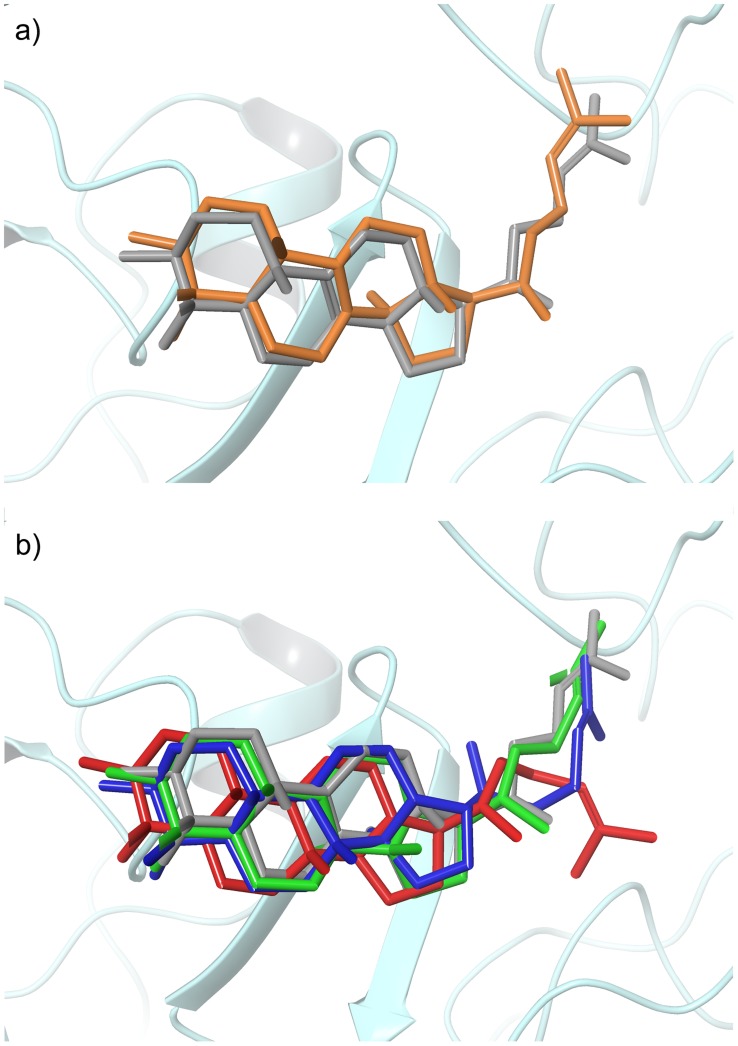
a) Superimposed view of the product lanosterol in the 1W6K crystal structure (grey) and the docking pose of C-I6 (the product precursor carbocation, c.f. **Figure 6b**; in orange); b) The docking poses of the second representative intermediates: A-I2 (blue), B-I2 (red) and C-I2 (lime), as well as lanosterol in the 1W6K crystal structure (grey, c.f. **Figure 6b**).

Figure 6b shows the carbocation intermediate docking results for the lanosterol synthase crystal structure (1W6K). The sequence identity between 1SQC and 1W6K is only 25%, and most of the active site residues are different. In this case, the intermediates I1 and I2 for reaction channel C receive the most favorable docking scores (I1 and I2 in Figure 6b). We also find that the product precursor C-I6 is the best binder among the intermediates along channel C (from C-I1 to C-I9; Figure 6b). Figure 7a shows the docking pose of the product precursor intermediate C-I6 (orange), which is in good agreement with the product lanosterol in the crystal structure (grey; RMSD 0.23 Å). Figure 7b shows the docking poses of the intermediates A-I2, B-I2, and C-I2. The pose of the correct intermediate C-I2 (lime; RMSD 0.42 Å) is more similar to that of lanosterol in the crystal structure (grey) than the poses of A-I2 and B-I2 (RMSD 0.63 Å and 0.86 Å), which differ from the crystal structure in the orientation of the 6,6,6,5-tetracyclic core (Figure 7b). Interestingly, C-I8, which can form the product cycloartenol (EC 5.4.99.8), is a non-binder, suggesting that the reaction will terminate at C-I6 or C-I7, both of which are precursors of lanosterol (C-I7 can also form other products such as parkeol and cycloartenol). These results suggest that the intermediates after C-I8 (e.g. C-I9, which is the product precursor of cucurbitadienol; EC 5.4.99.33) will be unlikely to occur. Hence, the docking results for 1W6K suggest that the carbocation docking approach could make qualitative, but meaningful, predictions concerning the end point of a reaction channel in some favorable cases. That is, the inability of a given binding site to significantly stabilize certain intermediates can, at a minimum, rule out downstream products. We explore this concept further below, using homology models to create a much larger test set.

### Docking against homology models

We further tested our approach by docking carbocationic intermediates against homology models of 54 triterpenoid synthases with annotations in Swiss-Prot (human-curated annotations). We exclude from consideration one triterpenoid synthase-like enzyme with a reported preference for a C_35_ substrate, both because it is not a triterpenoid synthase, and because it cannot be modeled reliably (only 25% sequence identity to 1SQC).

Guided by the results from docking carbocationic intermediates against the two available crystal structures, we use the docking scores for intermediates I1 and I2 to predict the reaction channel (see Methods for details). The overall success rate for reaction channel prediction of these sequences is 80% (Table 1). Details for each test case, including sequence alignments and docking scores, can be found in Table S1, S2 and S3. Three of the test cases are close homologs of 1W6K (88% sequence identity), and unsurprisingly, these are correctly predicted to follow Channel C, as does 1W6K. The remaining test cases have sequence identity to either 1SQC or 1W6K ranging between 33–49%, and thus are much more challenging.

**Table 1:** Statistics for the predictions using homology models.

Cluster^a^	Seq. Identity Range^b^	Number of models	Correct channel prediction	Success Rate
1SQC	>38%	4	4	100%
1W6K	>33%	50	39	78%
Total	-	54	43	80%

a c.f. Figure 4.

b calculated from the sequence alignment for homology modeling.

All 4 of the test cases in the 1SQC cluster were correctly predicted. Of these, 3 of 4 are squalene-hopene cyclases, i.e., the same function as 1SQC, upon which the homology models are based. However, the remaining case is correctly predicted to follow channel B (dammara-20,24-diene synthase). Note that sequence identity alone does not distinguish these cases; the dammara-20,24-diene synthase actually has slightly higher sequence identity to 1SQC than the hopene synthases.

Fifty of the test cases were in the 1W6K cluster, and thus their homology models were based on this structure (lanosterol synthase, channel C). The products of these enzymes correspond to a mix of channel B (27 cases) and channel C (23 cases). The overall accuracy of channel prediction is 78%; nine of the 11 incorrect predictions are based on homology models with 40% or lower sequence identity to 1W6K.

**Figure 8:**
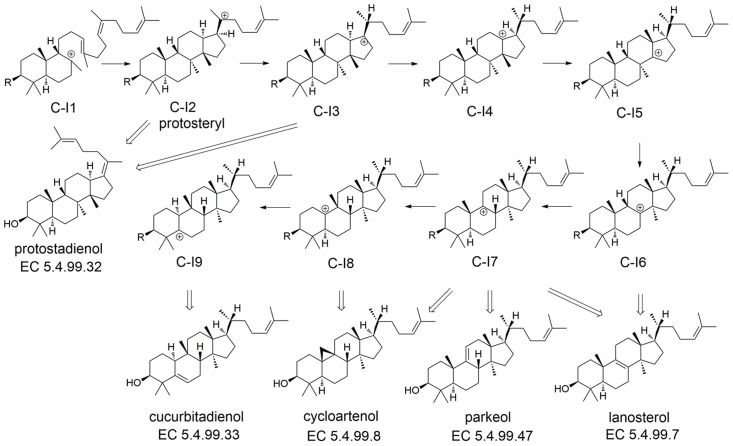
Intermediates and products of Channel C.

**Figure 9:**
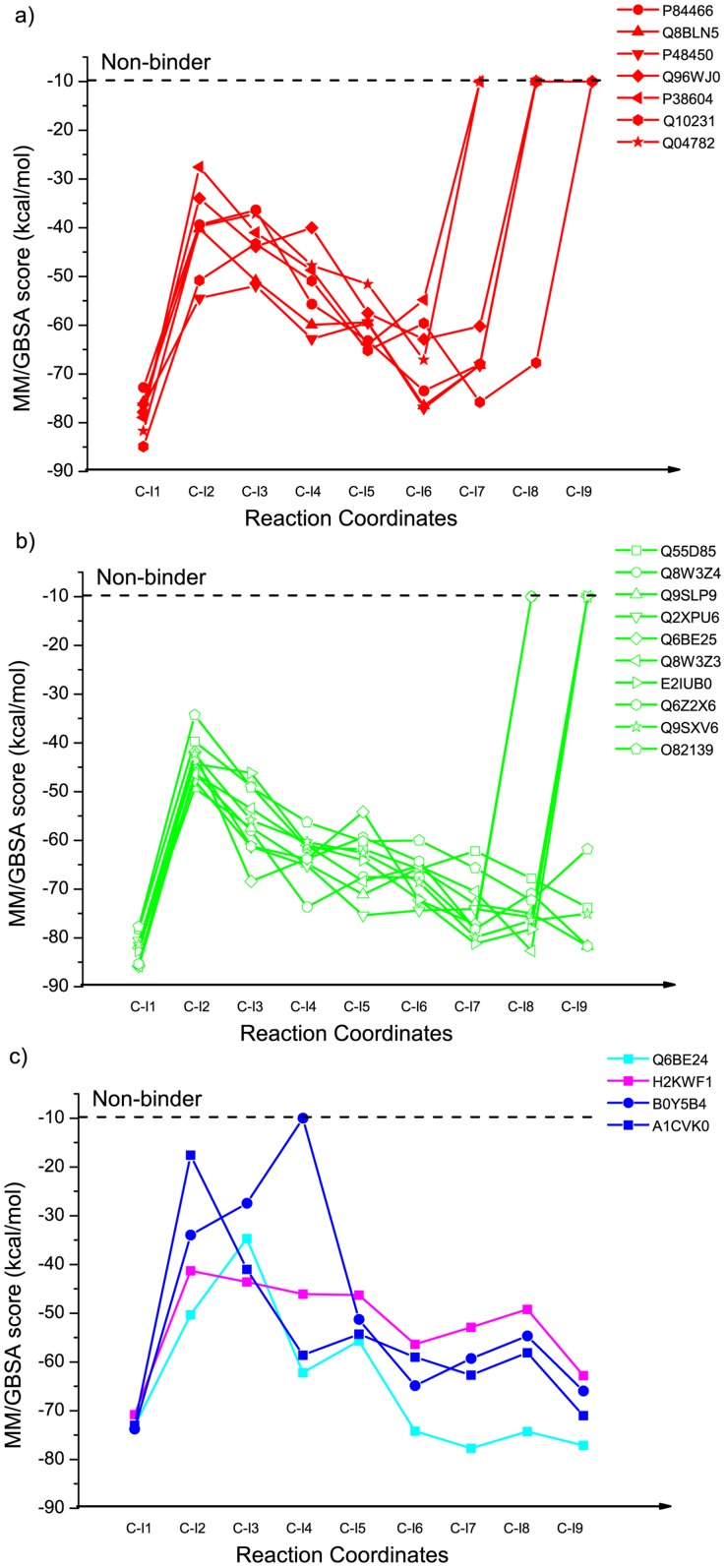
Docking score (MM/GBSA) of 9 carbocationic intermediates for 22 triterpenoid synthase homology models that follow channel C. Compounds that could not be successfully docked at all are arbitrarily assigned a docking score of −10 kcal/mol. Figure legend shows the UniProtKB IDs for the triterpenoid synthases. Panel a shows the docking scores against 8 lanosterol synthases (in red); panel b shows the docking scores against 10 cycloartenol synthases (in lime green); and panel c shows the docking scores against a cucurbitadienol synthase (in cyan), a parkeol synthase (in magenta) and 2 protostadienol synthases (in blue). Details c.f. Table S2.

Reaction channel prediction for 21 out of 23 triterpenoid synthases in the 1W6K cluster that follow Channel C are successful (Table S1d). For these 21 triterpenoid synthases, we further docked the downstream intermediates (Table S2, Figure 8 and Figure 9). The binding energy profiles, on average, follow a characteristic pattern where the docking scores are highly favorable for I1 in all cases, and much less so for I2, followed by gradually more favorable scores, on average, from I3 to I9. It should be kept in mind that these scores do not, at present, take into account the intrinsic (gas phase) relative energies of the carbocations (I2 being more stable than I1, for example). Nonetheless, the profiles for enzymes that generate different products show qualitative differences that correlate well in most cases with the product specificity.

For the triterpenoid synthases that produce lanosterol, the most favorable docking score (other than for I1) in 6 of 7 cases is either C-I6 or C-I7, both of which are product precursors for lanosterol (Figure 8 and Figure 9a). Moreover, in all cases, one or more of the intermediates subsequent to the intermediate with the most favorable docking score cannot be docked successfully into the binding site. Similarly, for the triterpenoid synthases that produce cycloartenol, 7 out of 10 models predict precursors C-I7 or C-I8 to have the most favorable docking scores (Figure 8 and Figure 9b). However, in 3 cases, C-I9 is predicted to have the most favorable docking score, and in 2 of these cases, there is no energy increase at C-I8. Thus, even in our very simple qualitative interpretation of these results, we consider these cases to be failures. The remaining 4 cases—enzymes that produce cucurbitadienol, parkeol, and protostadienol—are more ambiguous. One of the two protostadienol cases shows a strikingly different profile that is broadly consistent with being unable to proceed beyond C-I2 or C-I3, while the other case does not (Figure 9c). Overall, we conclude that carbocationic intermediate docking against homology models may be useful to make qualitative predictions concerning product specificity, but further improvements to the methodology are likely needed to provide robust predictions.

### Beyond enzyme function prediction: Guiding mutagenesis and studying enzyme mechanisms

Beyond enzyme function prediction, the current approach may have two other potential applications: 1) guiding mutagenesis experiments to alter the product specificity of an enzyme; and 2) exploring the catalytic mechanisms of enzymes. Although high-level quantum mechanical calculations are no doubt needed to make quantitative predictions, we illustrate here how the much simpler qualitative predictions from carbocation docking can nonetheless provide useful insights.

Specifically, we examine 3 mutants of 1SQC. The experimental data for these mutants were obtained from an earlier study [72], and our docking results are summarized in Table 2. The Y609C, Y609L and Y609S mutants generate aborted product A-P1 as the major product, and minor amounts of A-P2 and A-P4 (Table 2). The much lower yield of product A-P4 for the Y609X mutants suggests that the reaction channel leading to A-I4 is affected by Y609X mutations. We thus compared the MM/GBSA scores of intermediates of the Y609X mutants to those of wild type. As with all of the docking results, the scores should be interpreted qualitatively. In this case, the scores of A-I1, A-I2 and A-I4 do not vary significantly between wild-type and the mutants, while A-I3 becomes a much weaker binder for all three Y609X mutants. A comparison of the docking poses of A-I3 in the wild-type and the Y609C mutant Figure S6 also suggest that the Y609X mutants affect the binding of A-I3.

**Table 2:** Intermediate docking against the 1SQC mutants.

	Experimental Data^a^			Relative MM/GBSA Score^b^			
Enzyme	A-P1	A-P2^c^	A-P4	A-I1	A-I2	A-I3	A-I4
1SQC-wild	-	-	100%	0.0	0.0	0.0	0.0
1SQC-Y609C	72.3%	-	27.7%	+3.7	+2.0	+32.1	+1.7
1SQC-Y609L	42.9%	25.3%	30.2%	−1.0	−2.9	n.p.^d^	+0.5
1SQC-Y609S	70.1%	8.4%	21.6%	−3.1	+3.0	n.p.	+0.9
1SQC-L607K	80%^e^	-	-	+13.9	n.p.	n.p.	n.p.

a product percentage yield, c.f. ref [72].

b in kcal/mol, relative to WT docking scores.

c the total yield of all products from A-I2.

d n.p. means no pose can be obtained by docking.

e Product of this mutant is gamma-polypodatetraene.

**Figure 10:**
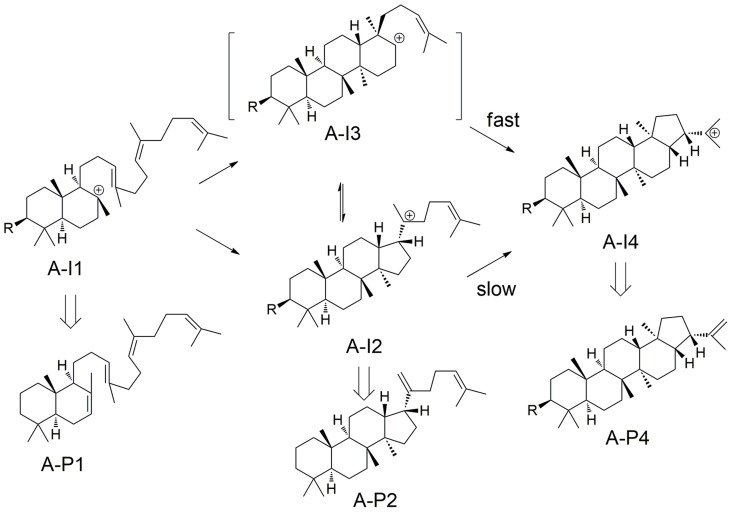
Key intermediates involved in the reaction channel leading to the hopanyl cation (A-I4), and products derived from these.

We interpret these results as follows (Figure 10). In a previous QM/MM study [39], the barrier height from A-I2 to A-I4 was computed to be 27.8 kcal/mol, while for the A-I3 like transition state that directly links A-I1 and A-I4, the barrier height was only 9.1 kcal/mol. Thus, for wild type, most A-I4 is likely generated through A-I3. In the mutants, binding of A-I3 is greatly destabilized, and we speculate that formation of A-I4 proceeds, much more slowly, through A-I2, and product formation from A-I1 and A-I2 competes with conversion to A-I4. Hence, our mechanistic findings from docking calculations are qualitatively consistent with the QM/MM results that the direct conversion from A-I1 to A-I4 is the major productive channel for 1SQC. The docking results are not accurate enough, however, to make any quantitative predictions concerning product distributions.

We also considered the L607K mutation of 1SQC, which generates gamma-polypodatetraene as the major product, presumably from A-I1. Consistent with this observation, only the A-I1 intermediate could be docked successfully. This appears to result from the strong repulsion between the positive charge on K607 and the carbocation on A-I2, A-I3 and A-I4.

## Discussion

Although the results obtained with the current methodology are more qualitative when compared to more rigorous methods such as QM/MM, the major advantage of docking carbocationic intermediates is its computational efficiency, which enables its application to large numbers of protein structures or models (over 50 in this proof-of-concept study). In the foreseeable future, these calculations will not replace experiments in providing reliable assignments of function, but as with other computational prediction methods, they can motivate experiments, or help to interpret the results. As in our prior work on enzyme function prediction, we anticipate that one of the most important uses will be identifying cases that are interesting or unusual, and thus high priorities for time- and resource-intensive *in vitro* or *in vivo* experiments (e.g., cyclases predicted to have novel specificity, or cases of convergent evolution).

Docking studies with carbocationic intermediates may also complement more accurate, but computationally intensive, QM/MM methods. For example, in cases where the reaction mechanism is poorly understood, the docking results may suggest plausible pathways that can be further explored by quantum mechanical methods (or perhaps more importantly, reject implausible pathways). Similarly, docking of carbocationic intermediates can be used to evaluate large numbers of possible mutations to identify ones more likely to modify product specificity in a desired manner.

We are aware of limitations of the current approach: 1) our carbocation library currently only considers the naturally occurring reaction channels, which cannot cover the complete chemical space of possible carbocationic rearrangements; 2) as our calculations are based on classical molecular mechanics and docking, the common limitations of MM and docking exist in all our calculations, e.g. the atomic charges are not polarizable (although we have used the QM-derived atomic charges); 3) other limitations such as neglecting the dynamics of the enzymes and the role of waters bound in the active site, which may also affect the final results; 4) the final deprotonation or hydration steps are not modeled. For the first limitation, we are developing an algorithm that can automatically generate all possible reaction channels, which will be published in due course. However, from our preliminary results, such efforts will dramatically increase the computational cost, due to the much larger size of the carbocation library.

## Methods

### Protein sequence similarity network

The sequence set of triterpenoid synthases were downloaded (October 2013) from Structure-Function Linkage Database [73] through the link http://sfld.rbvi.ucsf.edu/django/subgroup/1016/. The procedure for generating sequence similarity networks for these sequences follows our previous work [50]. Briefly, all pairwise BLAST *E*-values [69] were computed, and the sequence similarity networks were then generated by using Pythoscape [74]. A “quartile plot” is used to relate the average sequence similarity to the BLAST *E*-values (Figure S2). Cytoscape [75] is used for the visualization of the sequence similarity networks. In this visual representation, nodes represent sequences, and edges correspond to BLAST *E*-values that are smaller than a specified cutoff.

### Protein structure preparation and homology modeling

Crystal structures of triterpenoid synthases (PDB codes 1SQC [9], [10] and 1W6K [15]) were downloaded from the RCSB Protein Data Bank and processed using Schrödinger Protein Preparation Wizard [76], followed by restrained energy minimizations (RMSD tolerance 0.35 Å, in the presence of the co-crystallized ligand). All crystal water molecules were removed after the minimizations. Homology modeling procedures are similar to our previous work on the polyprenyl transferases [50]. Query sequences were aligned to the templates (1SQC or 1W6K, depending on sequence similarity) using PROMALS3D [77], and models were created by Schrödinger Prime [76], [78], [79]. In brief, the homology modeling procedure closes chain breaks associated with gaps in the sequence alignment by iterative application of the PLOP loop prediction algorithm, followed by side chain optimization (for all residues that are not identical between target and template in the sequence alignment), and complete energy minimization on all portions of the protein whose coordinates were either not taken from the template at all, or were modified during the model building procedure. All the homology models are then processed by using constrained minimizations (RMSD tolerance 0.35 Å, in the presence of the co-crystallized ligands) with Schrödinger Protein Preparation Wizard. The quality of the homology models is assessed by using the discrete optimized protein energy score (a statistical potential score for evaluating protein models) in MODELLER (Table S4) [80]. The OPLS 2005 force field [81], [82] was used throughout this study.

### Intermediate docking

**Figure 11:**
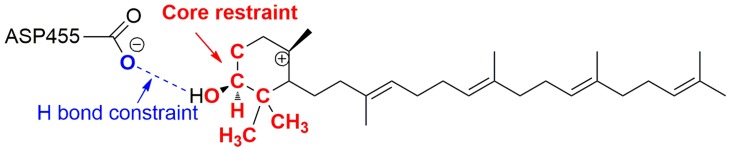
Example of constraints and restraints used during docking (residue numbering is for 1W6K).

The carbocationic intermediates were manually created and atomic charges were assigned using Jaguar [76], [83] quantum mechanical calculations (HF/6-31G*; geometry optimization in gas phase; electrostatic potential fitting). The carbocation library used in the current work is online available through the link www.jacobsonlab.org/carbocation/triterpene_docking_ligands.tar.gz (in ‘mol2’ format). The Schrödinger induced fit docking (IFD) protocol [84], [85] is used for all the docking calculations, with small modifications of default procedures and parameters. The IFD protocol consists of three stages: 1) Schrödinger Glide docking [86]–[89] with a reduced van der Waals scaling factor (0.5 for both receptor and ligand; top 5 poses are retained for the following steps); 2) minimization of the ligand as well as a conserved set of active site residues within 5 Å of the ligands defined by crystal structures (using the ‘RESIDUES_TO_ADD’ option of IFD; Table 3); 3) computation of MM/GBSA [78], [79] docking scores. To ensure the ligands are docked into the correct position, we applied constraints and core restraints during the initial Glide docking stage, which are essential for maintaining consistent poses of the carbocationic intermediates along the same reaction channel. For example, in the 1W6K crystal structure, we add a hydrogen bond constraint between the ligand and the key aspartate that protonates the oxido-squalene (D455 for 1W6K; c.f. Figure 11). In addition, we use a Glide core restraint (Figure 11 in red, 13 atoms, defined by ‘SMARTS’ pattern, i.e. “[#1][C-0X4]([#1])([#1])[C-0X4]([C-0X4]([#1])([#1])[#1])[C-0X4]([#1])([C-0X4]([#1])[#1])[O-0X2]”; 1.0 Å RMSD tolerance) to ensure that all the docked poses have the same orientation as the lanosterol ligand in the crystal structure (Figure 11). We also changed the Coulomb and van der Waals cutoff parameter during initial docking to a large positive number (‘CV_CUTOFF’ = 999999999.9 vs default 0.0), to retain more poses for the next stage. Both the IFD and MM/GBSA steps use ligand partial charges derived from quantum mechanics, as described above, for all energy calculations and minimizations. MM/GBSA, which is a force field-based scoring function (as opposed to empirical/knowledge-based scoring functions commonly used in docking), is used to accommodate the unusual carbocations studied in this work. That is, empirical or knowledge-based scoring functions will not have been trained on carbocation intermediates.

**Table 3:** Active site side chains minimized during the induced fit docking.^a^

Structure	Side chains (listed by residue number) undergoing energy minimization
1SQC	36, 42, 169, 170, 173, 261, 262, 263, 306, 307, 312, 365, 366, 374, 376, 377, 419, 420, 437, 438, 439, 440, 447, 448, 488, 489, 490, 495, 599, 600, 601, 605, 607, 609, 612
1W6K	98, 101, 103, 192, 230, 232, 233, 236, 237, 335, 336, 337, 338, 380, 381, 387, 444, 453, 455, 456, 502, 503, 518, 521, 524, 532, 533, 581, 587, 695, 696, 697, 702, 704

a These residues were within 5 Å of the co-crystalized product lanosterol of 1W6K after superposition of 1SQC and 1W6K. The “flexible” side chains when docking against homology models are those aligned to the flexible residues of the corresponding templates.

To ensure maximal consistence between the binding modes of I1 and I2, we first dock I2, and then copy the coordinates of I2 to I1, followed by energy minimization. We then check the key dihedral angle Φ_[C16-C17-C18-H18]_ (shown in Figure 5) of all the poses to ensure that the dihedral angles are consistent with those before energy minimization (Φ_[C16-C17-C18-H18]_>0 for A-I1, and Φ_[C16-C17-C18-H18]_<0 for B-I1 and C-I1).

### Hierarchical ranking

**Figure 12:**
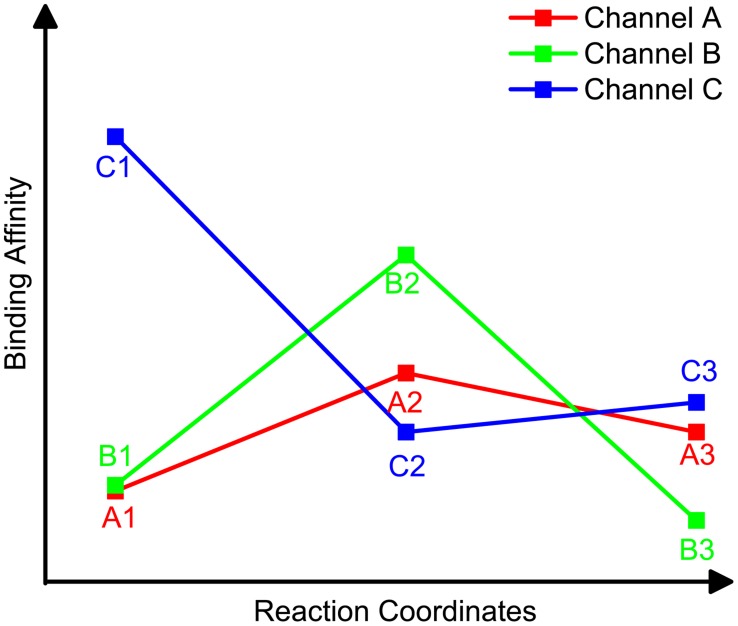
A hypothetical example output of the carbocation docking.

A hierarchical ranking strategy is used to rank different reaction channels and carbocationic intermediates (Figure 12). Figure 12 shows a hypothetical relative binding affinity (MM/GBSA score) profile obtained from carbocation docking along three different reaction channels. In Figure 12, the x-axis is a reaction coordinate (e.g. the conversion SubstrateA→A1→A2→A3→ProductA in Channel A), and the y-axis is the docking score. A1, B1, C1, A2, B2 and C2 are the first and second representative intermediates of reaction channels A, B and C, respectively. In this hypothetical example, the binding affinities of A1 and B1 are similar (<1 kcal/mol), and both are higher than that of C1; thus, the channel ranking in the first round is A = B>C. As for second representative intermediates, the docking score of A2 is more favorable than that of B2, and thus the final channel ranking is A>B>C. After the second representative intermediates, we are able to select the best reaction channel. All the intermediates along the best channel are then ranked by MM/GBSA (without considering further branching points).

## Supporting Information

Figure S1 Protein sequence similarity networks colored by EC number. Each node represents a protein sequence, and nodes are connected when the Blast *E*-value between the sequences is more significant than 10^−60^ (panel a) or 10^−220^/10^−300^ (panel b). Enzymes lacking SwissProt annotations are colored grey. Note that certain enzymes producing multiple products have been annotated by multiple EC numbers.
(TIF)

Figure S2 Quartile plots resulting from the all-by-all Blast of sequences in the triterpenoid synthase subgroup (in SFLD, it is called ‘Prenyltransferase Like 2’ subgroup, under the ‘IS-II superfamily’; available at http://sfld.rbvi.ucsf.edu/django/subgroup/1016/). Panel a shows the alignment length for different E values; Panel b shows the sequence identity for different E values; and Panel c shows the number of edges for different E values. More information about quartile plots can be found at http://efi.igb.illinois.edu/efi-est/tutorial_analysis.php
(TIF)

Figure S3 A comparison of the docking poses of A-I3 in the wild-type squalene-hopene cyclase (in blue) and its Y609C mutant (in red).
(TIF)

Figure S4 Chemical structures of the carbocationic intermediates of Channel B.
(TIF)

Table S1 MM/GBSA docking scores of I1 and I2 intermediates docked to crystal structures and homology models.
(DOCX)

Table S2 MM/GBSA docking scores of intermediates in channel C.
(DOCX)

Table S3 Sequence alignments used to generate homology models.
(DOCX)

Table S4 Quality assessment of homology models by using discrete optimized protein energy (DOPE) score.
(DOCX)

Table S5 RMSD for the active site residues of crystal structures and those in the IFD.
(DOCX)
