# Supplementary figures and images for: Predicting the Functions and Specificity of Triterpenoid Synthases: A Mechanism-Based Multi-intermediate Docking Approach
Source: PLoS Comput Biol. 2014 Oct 9;10(10):e1003874. doi: 10.1371/journal.pcbi.1003874 (PMC4191879; doi:10.1371/journal.pcbi.1003874)

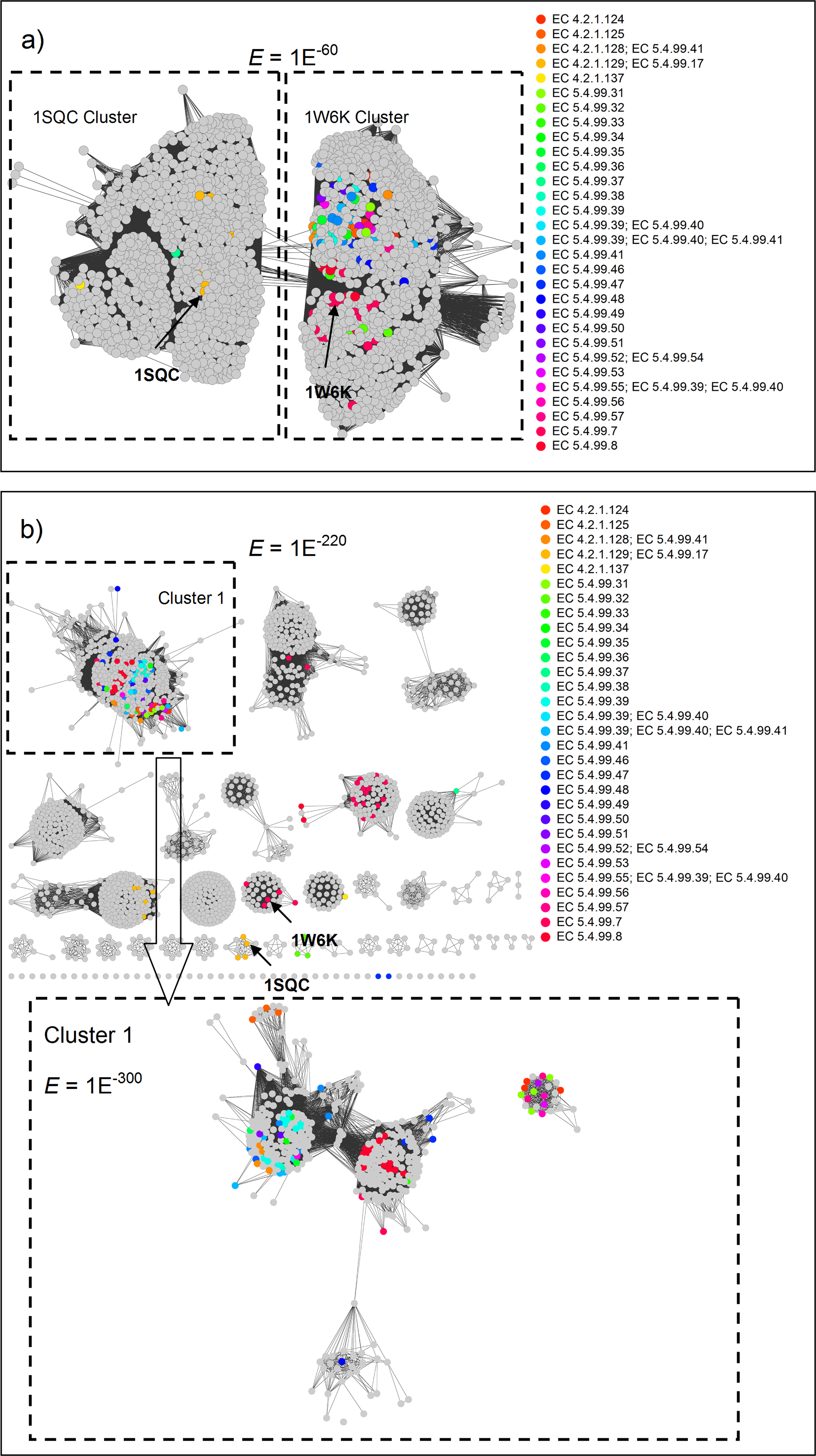

Supplement: Figure S1 — Protein sequence similarity networks colored by EC number. Each node represents a protein sequence, and nodes are connected when the Blast E-value between the sequences is more significant than 10−60 (panel a) or 10−220/10−300 (panel b). Enzymes lacking SwissProt annotations are colored grey. Note that certain enzymes producing multiple products have been annotated by multiple EC numbers. (TIF) [file pcbi.1003874.s001.tif]

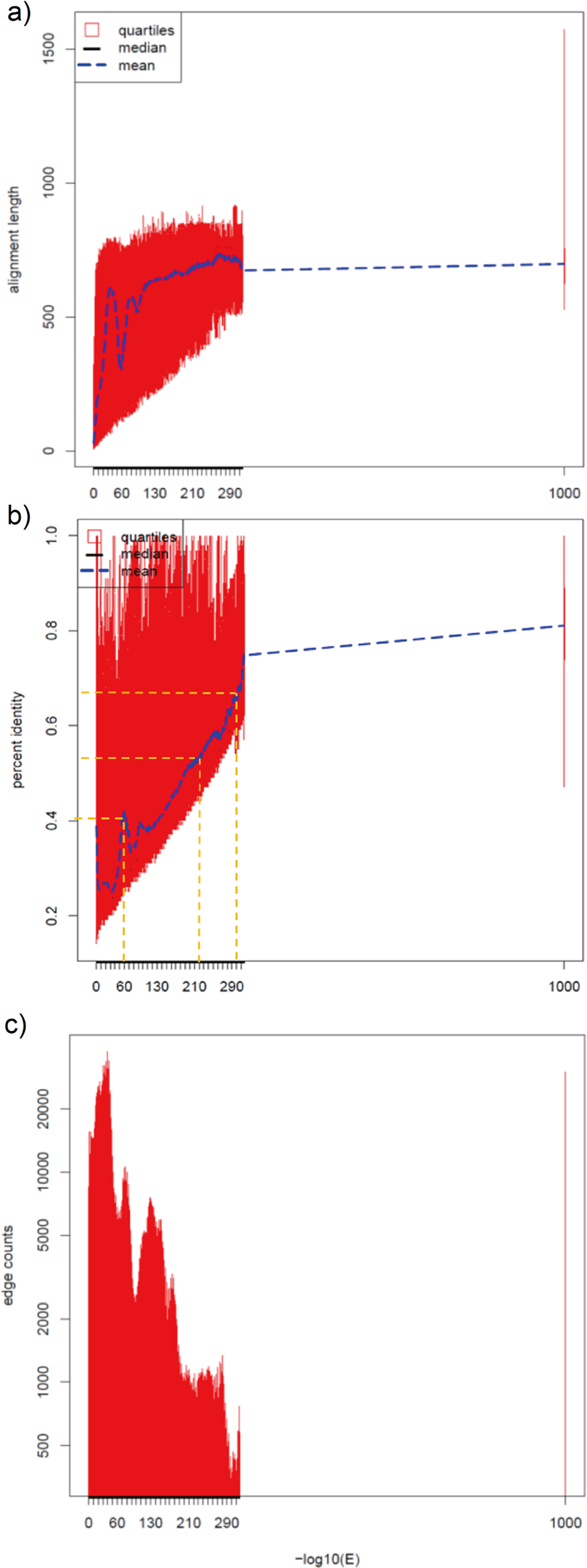

Supplement: Figure S2 — Quartile plots resulting from the all-by-all Blast of sequences in the triterpenoid synthase subgroup (in SFLD, it is called ‘Prenyltransferase Like 2’ subgroup, under the ‘IS-II superfamily’; available at http://sfld.rbvi.ucsf.edu/django/subgroup/1016/). Panel a shows the alignment length for different E values; Panel b shows the sequence identity for different E values; and Panel c shows the number of edges for different E values. More information about quartile plots can be found at http://efi.igb.illinois.edu/efi-est/tutorial_analysis.php (TIF) [file pcbi.1003874.s002.tif]

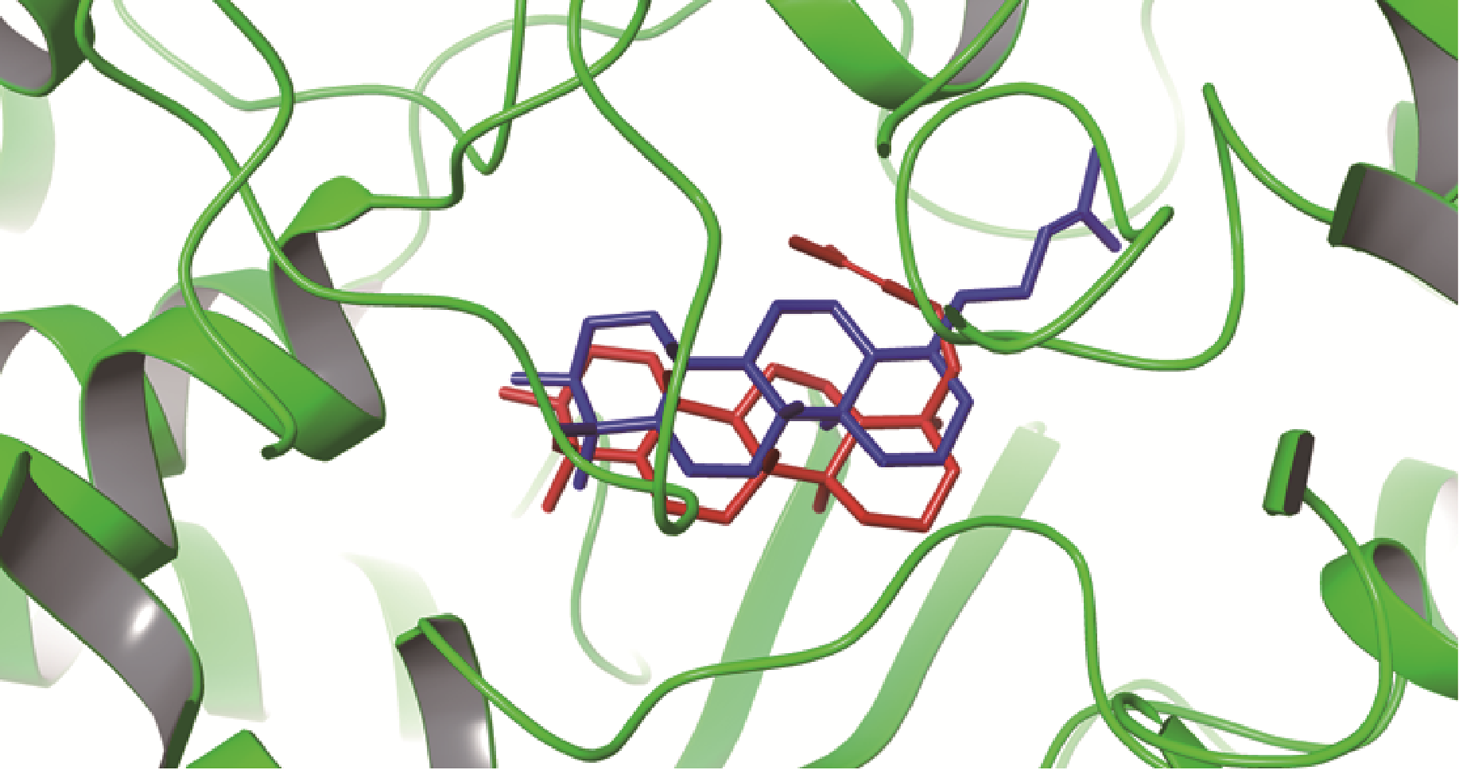

Supplement: Figure S3 — A comparison of the docking poses of A-I3 in the wild-type squalene-hopene cyclase (in blue) and its Y609C mutant (in red). (TIF) [file pcbi.1003874.s003.tif]

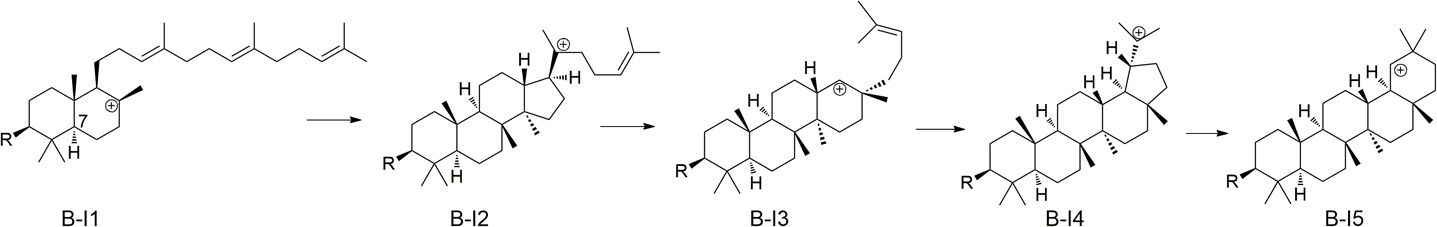

Supplement: Figure S4 — Chemical structures of the carbocationic intermediates of Channel B. (TIF) [file pcbi.1003874.s004.tif]
