# Supplementary material for: Predicting the Functions and Specificity of Triterpenoid Synthases: A Mechanism-Based Multi-intermediate Docking Approach
Source: PLoS Comput Biol. 2014 Oct 9;10(10):e1003874. doi: 10.1371/journal.pcbi.1003874 (PMC4191879; doi:10.1371/journal.pcbi.1003874)
Supplement: Table S1 — MM/GBSA docking scores of I1 and I2 intermediates docked to crystal structures and homology models. (DOCX) [file pcbi.1003874.s005.docx]

Table S1. MM/GBSA docking scores of intermediates I1 and I2 in reaction channels A, B, and C.

Table S1a. Induced fit docking of intermediates against crystal structures (using QM charges^a^ for the ligands)

| PDB | A-I1 | B-I1 | C-I1 | D-I1 | A-I2 | B-I2 | C-I2 | D-I2 |
| --- | --- | --- | --- | --- | --- | --- | --- | --- |
| 1SQC | **-57.8^b^** | n.p.^c^ | -41.9 | n.p. | -33.2 | n.p. | -6.0 | n.p. |
| 1W6K | -62.7 | -62.3 | **-73.5** | -59.1 | -35.0 | -32.8 | -43.3 | -46.3 |

^a^QM charges are electrostatic potential atomic charges derived at the HF/6-31G* level.

^b^bolded numbers are the 'decision-making' intermediates

^c^n.p. means no pose can be generated

Table S1b. Induced fit docking of intermediates against crystal structures (using MM charges^a^ for the ligands)

| PDB | A-I1 | B-I1 | C-I1 | D-I1 | A-I2 | B-I2 | C-I2 | D-I2 |
| --- | --- | --- | --- | --- | --- | --- | --- | --- |
| 1SQC | **-52.2** | n.p. | -47.6 | n.p. | -38.3 | n.p. | -26.8 | n.p. |
| 1W6K | -68.2 | -69.6 | **-72.8** | -66.3 | -38.1 | -46.7 | -44.1 | -54.8 |

^a^MM charges are OPLS2005 charges, generated by Schrödinger Ligprep

^b^bolded numbers are the 'decision-making' intermediates

^c^n.p. means no pose can be generated

Table S1c. Reaction channel predictions for triterpenoid synthases in the 1SQC cluster (yellow color indicates incorrect predictions; bolded numbers indicate the intermediates used to predict the reaction channel, using the procedure described in Methods)

| GI | Uniprot | SwissProt | EC number | common name | Sequence  identity to 1SQC | SwissProt /Predicted  Channel | A-I1 | B-I1 | C-I1 | D-I1 | A-I2 | B-I2 | C-I2 | D-I2 |
| --- | --- | --- | --- | --- | --- | --- | --- | --- | --- | --- | --- | --- | --- | --- |
| 357580429 | B3Y522 | DCD_DRYCA | 5.4.99.37 | dammara-20,24-diene | 42% | B/B | n.p. | **-42.0** | -27.9 | -31.6 | n.p.^a^ | 1.9 | 4.3 | 18.2 |
| 6466213 | P33990 | SQHC_ZYMMO | 4.2.1.129; 5.4.99.17 | hopanol; hop-22(29)-ene | 39% | A/A | **-40.0** | n.p. | -31.0 | -34.0 | -18.4 | n.p. | -19.2 | -24.2 |
| 16519641 | P55348 | SQHC_RHISN | 4.2.1.129; 5.4.99.17 | hopanol; hop-22(29)-ene | 38% | A/A | **-41.4** | -38.4 | -33.4 | n.p. | -25.1 | -18.2 | 2.6 | n.p. |
| 2113823 | P54924 | SQHC_BRAJA | 4.2.1.129; 5.4.99.17 | hopanol; hop-22(29)-ene | 38% | A/A | **-48.6** | -40.1 | -40.1 | -37.6 | -20.9 | -25.2 | -39.4 | -13.8 |

^a^n.p. means no pose can be generated

Table S1d. Reaction channel predictions for triterpenoid synthases in the 1W6K cluster (yellow color indicates incorrect predictions; bolded numbers indicate the intermediates used to predict the reaction channel, using the procedure described in Methods)

| GI | Uniprot | SwissProt | EC number | common name | Sequence identity to 1W6K | SwissProt /Predicted Channel | A-I1 | B-I1 | C-I1 | D-I1 | A-I2 | B-I2 | C-I2 | D-I2 |
| --- | --- | --- | --- | --- | --- | --- | --- | --- | --- | --- | --- | --- | --- | --- |
| 114053041 | P84466 | ERG7_BOVIN | 5.4.99.7 | lanosterol | 86% | C/C | -67.8 | -61.3 | **-72.8** | -57.4 | -41.0 | -38.5 | -39.4 | -50.7 |
| 26346907 | Q8BLN5 | ERG7_MOUSE | 5.4.99.7 | lanosterol | 86% | C/C | n.p. | -74.7 | **-75.8** | -55.4 | n.p. | -37.6 | -40.2 | -41.5 |
| 13591981 | P48450 | ERG7_RAT | 5.4.99.7 | lanosterol | 85% | C/C | n.p. | -56.4 | **-76.6** | -66.4 | n.p. | -41.1 | -54.5 | -48.4 |
| 15076955 | Q96WJ0 | ERG7_PNECA | 5.4.99.7 | lanosterol | 46% | C/C | -55.4 | -73.4 | **-77.8** | -66.0 | -16.8 | -38.5 | -34.0 | -46.7 |
| 167295241 | P38604 | ERG7_YEAST | 5.4.99.7 | lanosterol | 41% | C/C | n.p. | -72.2 | **-78.9** | -66.5 | n.p. | -26.4 | -27.6 | -30.2 |
| 63054562 | Q10231 | ERG7_SCHPO | 5.4.99.7 | lanosterol | 42% | C/C | 28.4 | -77.1 | **-84.9** | -58.8 | -48.2 | -43.6 | -50.8 | -38.8 |
| 68466833 | Q04782 | ERG7_CANAL | 5.4.99.7 | lanosterol | 40% | C/C | -68.4 | n.p. | **-81.7** | -60.3 | -25.3 | n.p. | -39.7 | -41.3 |
| 66825783 | Q55D85 | CAS1_DICDI | 5.4.99.8 | cycloartenol | 49% | C/C | -59.9 | -74.1 | **-78.2** | -61.2 | -46.4 | -47.3 | -39.7 | -30.5 |
| 300591899 | O82139 | CAS1_PANGI | 5.4.99.8 | cycloartenol | 45% | C/C | -61.5 | n.p. | **-77.7** | -41.6 | -26.8 | n.p. | -34.3 | -23.9 |
| 300592019 | Q9SXV6 | CAS1_GLYGL | 5.4.99.8 | cycloartenol | 45% | C/C | -73.0 | -79.5 | **-81.4** | -53.7 | -43.4 | -46.3 | -42.3 | -45.8 |
| 300591983 | Q8W3Z4 | CAS1_BETPL | 5.4.99.8 | cycloartenol | 45% | C/C | -59.6 | n.p. | **-85.3** | -58.6 | -41.9 | n.p. | -46.2 | -38.2 |
| 300592007 | Q9SLP9 | CAS1_LUFCY | 5.4.99.8 | cycloartenol | 45% | C/C | n.p. | -80.2 | **-81.5** | -65.8 | n.p. | -47.5 | -49.3 | -56.6 |
| 82468805 | Q2XPU6 | CAS1_RICCO | 5.4.99.8 | cycloartenol | 44% | C/C | n.p. | n.p. | **-80.6** | -56.6 | n.p. | n.p. | -47.7 | -48.8 |
| 300591913 | Q6BE25 | CAS1_CUCPE | 5.4.99.8 | cycloartenol | 44% | C/C | -70.3 | n.p. | **-85.8** | -67.0 | -47.4 | n.p. | -41.9 | -43.9 |
| 300591981 | Q8W3Z3 | CAS2_BETPL | 5.4.99.8 | cycloartenol | 45% | C/C | n.p. | n.p. | **-85.8** | -60.9 | n.p. | n.p. | -46.4 | -34.3 |
| 6090879 | Q6Z2X6 | CAS_ORYSJ | 5.4.99.8 | cycloartenol | 43% | C/C | -74.6 | n.p. | **-85.3** | -49.0 | -45.5 | n.p. | -43.4 | -41.3 |
| 119499584 | A1CVK0 | PDSA_NEOFI | 5.4.99.32 | (17Z)-protosta-17(20),24-dien-3beta-ol | 40% | C/C | -69.1 | n.p. | **-73.0** | -66.8 | -41.4 | n.p. | -17.6 | -35.5 |
| 70993016 | B0Y5B4 | PDSA_ASPFC | 5.4.99.32 | (17Z)-protosta-17(20),24-dien-3beta-ol | 40% | C/C | -67.7 | n.p. | **-73.8** | -58.7 | -23.7 | n.p. | -34.0 | -44.2 |
| 300591911 | Q6BE24 | CUCS_CUCPE | 5.4.99.33 | cucurbitadienol | 43% | C/C | n.p. | -68.6 | **-73.2** | -46.0 | n.p. | -45.3 | -50.3 | -33.5 |
| 108864084 | H2KWF1 | PAKSY_ORYSJ | 5.4.99.47 | parkeol | 39% | C/C | -48.9 | n.p. | **-70.8** | -48.4 | -34.0 | n.p. | -41.3 | -34.9 |
| 300807982 | E2IUB0 | CASS_KALDA | 5.4.99.8 | cycloartenol | 44% | C/C | -77.9 | n.p. | **-82.7** | -57.3 | -51.0 | n.p. | -44.3 | 20.6 |
| 30699377 | Q8RWT0 | LUP2_ARATH | 5.4.99.39; 5.4.99.40; 5.4.99.41 | beta-amyrin; alpha-amyrin; lupeol | 39% | B/B | -27.2 | **-58.5** | -51.0 | -26.1 | -22.9 | -30.2 | -42.3 | -30.0 |
| 353678016 | A8CDT2 | BAS_BRUGY | 5.4.99.39 | beta-amyrin | 41% | B/B | -44.7 | **-51.0** | n.p. | -38.8 | -40.3 | -38.5 | n.p. | -33.5 |
| 118901781 | O82146 | BAMS2_PANGI | 5.4.99.39 | beta-amyrin | 40% | B/B | -49.0 | **-54.7** | -51.8 | n.p. | -45.1 | -47.0 | -51.4 | n.p. |
| 350538403 | E7DN64 | DAMS_SOLLC | 5.4.99.55 | delta-amyrin | 39% | B/B | -35.9 | **-74.4** | -66.8 | -59.2 | -38.0 | -37.8 | -54.2 | -44.9 |
| 300807980 | E2IUA9 | LUPS_KALDA | 5.4.99.41 | lupeol | 39% | B/B | -40.6 | **-64.6** | n.p. | 46.6 | -50.4 | -51.8 | n.p. | -39.2 |
| 82468803 | Q2XPU7 | LUPS_RICCO | 5.4.99.41 | lupeol | 37% | B/B | -46.1 | **-71.5** | -64.0 | -25.0 | -39.9 | -40.3 | -41.9 | -46.7 |
| 353678133 | A8C981 | TARS_RHISY | 5.4.99.35 | taraxerol | 37% | B/B | -58.6 | **-63.8** | n.p. | -43.1 | -33.0 | -30.2 | n.p. | -34.9 |
| 240256372 | Q9LVY2 | PEN3_ARATH | 5.4.99.56 | tirucalla-7,24-dien-3beta-ol | 34% | B/B | -46.9 | -59.5 | -58.8 | -46.8 | -35.4 | **-43.0** | -39.3 | -13.9 |
| 270303608 | Q08IT1 | DADIS_PANGI | 4.2.1.125 | dammarenediol II | 37% | B/B | -54.8 | **-58.6** | -45.6 | -50.5 | -30.3 | -23.9 | -20.4 | -8.8 |
| 300807978 | E2IUA8 | FRIES_KALDA | 5.4.99.50 | friedelin | 40% | B/B | -36.3 | **-54.5** | n.p. | -36.7 | -34.4 | -33.7 | n.p. | -39.5 |
| 403377906 | B9X0J1 | STBOS_STERE | 5.4.99.51 | baccharis oxide | 37% | B/B | -41.1 | **-47.6** | -20.0 | -37.2 | -36.2 | -29.7 | -20.8 | 0.9 |
| 350538549 | E7DN63 | BAMS_SOLLC | 5.4.99.39 | beta-amyrin | 40% | B/B | -40.7 | **-47.0** | -30.1 | -38.0 | -37.5 | -36.6 | -60.0 | -31.9 |
| 300591997 | Q9LRH7 | ABAMS_PEA | 5.4.99.39; 5.4.99.40 | beta-amyrin; alpha-amyrin | 38% | B/B | -44.2 | **-54.5** | -46.5 | -39.9 | -32.3 | -47.4 | -38.6 | -42.4 |
| 300807976 | E2IUA7 | GLUTS_KALDA | 5.4.99.49 | glutinol | 39% | B/B | -46.0 | **-54.2** | -50.9 | -47.0 | -33.8 | -37.2 | -49.9 | -46.6 |
| 300591999 | Q9LRH8 | BAMS_PEA | 5.4.99.39 | beta-amyrin | 40% | B/B | -42.7 | **-49.8** | -45.7 | -29.0 | -11.1 | -29.0 | -42.5 | -26.3 |
| 211926830 | B6EXY6 | LUP4_ARATH | 5.4.99.39 | beta-amyrin | 39% | B/B | -38.5 | **-66.4** | -55.4 | -29.1 | -39.5 | -56.0 | -23.7 | -36.7 |
| 353558864 | A8C980 | GERS_RHISY | 5.4.99.34 | germanicol | 41% | B/B | -42.1 | **-52.8** | n.p. | -50.2 | -49.2 | -44.5 | n.p. | -48.4 |
| 15233798 | O23390 | BARS1_ARATH | 5.4.99.57 | baruol | 33% | B/B | -35.0 | -42.7 | -43.0 | -27.7 | -14.9 | **-36.3** | -32.9 | -24.1 |
| 15218390 | Q9SYN1 | PEN6_ARATH | 5.4.99.52; 5.4.99.54 | alpha-seco-amyrin; beta-seco-amyrin | 35% | B/A | **-55.8** | -53.5 | -54.5 | -46.4 | -37.7 | -34.2 | -21.9 | -29.4 |
| 300592003 | Q9MB42 | BAMS_GLYGL | 5.4.99.39 | beta-amyrin | 40% | B/C | -39.9 | n.p. | **-51.7** | -43.2 | -41.8 | n.p. | -48.0 | -35.0 |
| 300591979 | Q8W3Z2 | LUPS_BETPL | 5.4.99.41 | lupeol | 39% | B/C | -59.3 | -47.0 | **-61.1** | -43.6 | -17.1 | -31.9 | -14.5 | -18.1 |
| 300807974 | E2IUA6 | TARS_KALDA | 5.4.99.35 | taraxerol | 41% | B/C | -2.8 | -45.6 | **-58.7** | -39.3 | -20.2 | -17.2 | -23.3 | -24.3 |
| 30699380 | Q9C5M3 | LUP1_ARATH | 4.2.1.128; 5.4.99.41 | lupan-3beta,20-diol; lupeol | 39% | B/C | -49.7 | -56.3 | **-62.9** | -39.0 | -43.6 | -45.5 | -41.4 | -28.5 |
| 257623103 | Q764T8 | LUPS_GLYGL | 5.4.99.41 | lupeol | 39% | B/C | -43.0 | -48.5 | **-56.2** | -34.1 | -31.7 | -26.8 | -35.1 | -22.8 |
| 300591987 | Q948R6 | IMFS_LUFCY | 5.4.99.36 | isomultiflorenol | 38% | B/C | -42.3 | n.p. | **-47.6** | -26.7 | -20.6 | n.p. | -12.2 | -33.6 |
| 353558692 | A8CDT3 | LUPS_BRUGY | 5.4.99.41 | lupeol | 37% | B/A | **-47.2** | n.p. | -46.4 | -40.3 | -36.0 | n.p. | -38.8 | -39.6 |
| 300591977 | Q8W3Z1 | BAMS_BETPL | 5.4.99.39 | beta-amyrin | 40% | B/C | -34.0 | n.p. | **-53.0** | -23.4 | -38.6 | n.p. | -39.9 | -41.8 |
| 260037884 | Q1G1A4 | LAS1_ARATH | 5.4.99.7 | lanosterol | 40% | C/B | -59.6 | -59.5 | -31.3 | -45.7 | -32.4 | **-33.7** | -28.0 | -3.9 |
| 452446 | P38605 | CAS1_ARATH | 5.4.99.8 | cycloartenol | 44% | C/A | **-85.4** | -78.6 | -81.5 | -71.7 | -44.4 | -42.7 | -49.0 | -56.5 |

Table S1e. Glide XP scores for the key intermediates of the crystal structures. The poses are the same as those in Table S3a.

| PDB | A-I1 | B-I1 | C-I1 | D-I1 | A-I2 | B-I2 | C-I2 | D-I2 |
| --- | --- | --- | --- | --- | --- | --- | --- | --- |
| 1SQC | **-9.509** | n.p. | -5.236 | n.p. | -4.612 | n.p. | -3.077 | n.p. |
| 1W6K | -9.079 | -9.134 | **-10.382** | -7.125 | -4.574 | -8.284 | -7.431 | -5.965 |
