# Supplementary material for: Predicting the Functions and Specificity of Triterpenoid Synthases: A Mechanism-Based Multi-intermediate Docking Approach
Source: PLoS Comput Biol. 2014 Oct 9;10(10):e1003874. doi: 10.1371/journal.pcbi.1003874 (PMC4191879; doi:10.1371/journal.pcbi.1003874)
Supplement: Table S2 — MM/GBSA docking scores of intermediates in channel C. (DOCX) [file pcbi.1003874.s006.docx]

Table S2. MM/GBSA docking scores of intermediates from channel C for homology models: yellow cells represent the product precursor intermediates





| GI | Uniprot | SwissProt | EC number | common name | Sequence identity to 1W6K | SwissProt /Predicted Channel | C-I1 | C-I2 | C-I3 | C-I4 | C-I5 | C-I6 | C-I7 | C-I8 | C-I9 |
| --- | --- | --- | --- | --- | --- | --- | --- | --- | --- | --- | --- | --- | --- | --- | --- |
| 114053041 | P84466 | ERG7_BOVIN | 5.4.99.7 | lanosterol | 86% | C/C | -72.8 | -39.4 | -36.4 | -55.7 | -63.2 | -73.5 | -68.0 | n.p. | -74.0 |
| 26346907 | Q8BLN5 | ERG7_MOUSE | 5.4.99.7 | lanosterol | 86% | C/C | -75.8 | -40.2 | -50.9 | -59.9 | -59.4 | -76.5 | -68.2 | n.p. | -78.6 |
| 13591981 | P48450 | ERG7_RAT | 5.4.99.7 | lanosterol | 85% | C/C | -76.6 | -54.5 | -51.9 | -62.8 | -59.5 | -77.0 | -68.2 | n.p. | -71.3 |
| 15076955 | Q96WJ0 | ERG7_PNECA | 5.4.99.7 | lanosterol | 46% | C/C | -77.8 | -34.0 | -43.9 | -40.0 | -57.5 | -62.9 | -60.2 | n.p. | n.p. |
| 167295241 | P38604 | ERG7_YEAST | 5.4.99.7 | lanosterol | 41% | C/C | -78.9 | -27.6 | -41.0 | -48.7 | -64.0 | -54.8 | n.p. | -57.2 | -55.5 |
| 63054562 | Q10231 | ERG7_SCHPO | 5.4.99.7 | lanosterol | 42% | C/C | -84.9 | -50.8 | -43.3 | -50.9 | -65.2 | -59.6 | -75.8 | -67.7 | n.p. |
| 68466833 | Q04782 | ERG7_CANAL | 5.4.99.7 | lanosterol | 40% | C/C | -81.7 | -39.7 | -37.2 | -47.7 | -51.6 | -67.1 | n.p. | -66.6 | n.p. |
| 66825783 | Q55D85 | CAS1_DICDI | 5.4.99.8 | cycloartenol | 49% | C/C | -78.2 | -39.7 | -48.9 | -61.5 | -61.8 | -65.9 | -62.2 | -67.8 | -73.8 |
| 300591899 | O82139 | CAS1_PANGI | 5.4.99.8 | cycloartenol | 45% | C/C | -77.7 | -34.3 | -49.1 | -56.3 | -60.2 | -60.0 | -65.7 | -72.3 | -61.8 |
| 300592019 | Q9SXV6 | CAS1_GLYGL | 5.4.99.8 | cycloartenol | 45% | C/C | -81.4 | -42.3 | -55.9 | -60.3 | -62.6 | -68.6 | -79.9 | -76.4 | -75.1 |
| 300591983 | Q8W3Z4 | CAS1_BETPL | 5.4.99.8 | cycloartenol | 45% | C/C | -85.3 | -46.2 | -58.0 | -73.7 | -67.5 | -67.6 | -78.0 | n.p. | n.p. |
| 300592007 | Q9SLP9 | CAS1_LUFCY | 5.4.99.8 | cycloartenol | 45% | C/C | -81.5 | -49.3 | -57.4 | -64.8 | -71.1 | -65.7 | -73.1 | -75.0 | -81.7 |
| 82468805 | Q2XPU6 | CAS1_RICCO | 5.4.99.8 | cycloartenol | 44% | C/C | -80.6 | -47.7 | -61.3 | -65.2 | -75.4 | -74.4 | -74.1 | -75.8 | n.p. |
| 300591913 | Q6BE25 | CAS1_CUCPE | 5.4.99.8 | cycloartenol | 44% | C/C | -85.8 | -41.9 | -68.4 | -63.8 | -54.2 | -72.3 | -77.2 | n.p. | -88.4 |
| 300591981 | Q8W3Z3 | CAS2_BETPL | 5.4.99.8 | cycloartenol | 45% | C/C | -85.8 | -46.4 | -53.5 | -61.0 | -68.4 | -65.3 | -70.5 | -82.7 | n.p. |
| 300807982 | E2IUB0 | CASS_KALDA | 5.4.99.8 | cycloartenol | 44% | C/C | -82.7 | -44.3 | -46.2 | -60.9 | -64.1 | -72.2 | -81.2 | -78.2 | n.p. |
| 6090879 | Q6Z2X6 | CAS_ORYSJ | 5.4.99.8 | cycloartenol | 43% | C/C | -85.3 | -43.4 | -61.2 | -63.8 | -59.4 | -64.3 | -78.2 | -70.9 | -81.7 |
| 119499584 | A1CVK0 | PDSA_NEOFI | 5.4.99.32 | (17Z)-protosta-17(20),24-dien-3beta-ol | 40% | C/C | -73.0 | -17.6 | -41.0 | -58.6 | -54.3 | -59.0 | -62.7 | -58.1 | -71.0 |
| 70993016 | B0Y5B4 | PDSA_ASPFC | 5.4.99.32 | (17Z)-protosta-17(20),24-dien-3beta-ol | 40% | C/C | -73.8 | -34.0 | -27.5 | n.p. | -51.3 | -64.9 | -59.3 | -54.7 | -66.0 |
| 300591911 | Q6BE24 | CUCS_CUCPE | 5.4.99.33 | cucurbitadienol | 43% | C/C | -73.2 | -50.3 | -34.7 | -62.2 | -55.7 | -74.2 | -77.7 | -74.3 | -77.1 |
| 108864084 | H2KWF1 | PAKSY_ORYSJ | 5.4.99.47 | parkeol | 39% | C/C | -70.8 | -41.3 | -43.6 | -46.1 | -46.3 | -56.4 | -52.9 | -49.2 | -62.8 |
