# Supplementary material for: Predicting the Functions and Specificity of Triterpenoid Synthases: A Mechanism-Based Multi-intermediate Docking Approach
Source: PLoS Comput Biol. 2014 Oct 9;10(10):e1003874. doi: 10.1371/journal.pcbi.1003874 (PMC4191879; doi:10.1371/journal.pcbi.1003874)
Supplement: Table S3 — Sequence alignments used to generate homology models. (DOCX) [file pcbi.1003874.s007.docx]

Table S3. Sequence alignment used to create homology models

Table S3a. Enzymes in the 1SQC cluster

| GI | Alignment |
| --- | --- |
| 357580429 | Targ: LPYNQDFYNEDEALKDDHCEGAGNVSNPPTLDEAIKRSQDFLLSQQYPEGYWWAELEGNPTITSHTVILY  1SQC: ---------------------AEQLVEAPAYARTLDRAVEYLLSCQKDEGYWWGPLLSNVTMEAEYVLLC  Targ: KILGIEDEYPMDKMEKYLRRMQCIHGGWELFYGDGGQLSVTIESYVALRLLNVPPTDPALKKALKFIIDK  1SQC: HILDRVDRDRMEKIRRYLLHEQREDGTWALYPGGPPDLDTTIEAYVALKYIGMSRDEEPMQKALRFIQSQ  Targ: GGVXKSRMFTKICLALLGCFDWRGIPSLPPWVMLLPGWFLSSIYETACWARGCVVPLIVVFDKKPVFKVS  1SQC: GGIESSRVFTRMWLALVGEYPWEKVPMVPPEIMFLGKRMPLNIYEFGSWARATVVALSIVMSRQPVFPLP  Targ: PEVSFDELYAEGREHACKTLPFCGDWTSHFFIAVDRVFKMMERLGVVPFQQWGIREAEKWLLERQEDTGD  1SQC: ERARVPELYETDVPPRRRGAKGGGGW---IFDALDRALHGYQKLSVHPFRRAAEIRALDWLLERQAGDGS  Targ: FLGVYPPMFYSVVCMKTLGYEVTDPVVRRALLSFKKFSIERAD-ECSVQSSLSPVWDTALVVRSLVESGL  1SQC: WGGIQPPWFYALIALKILDMT-QHPAFIKGWEGLELYGVELDYGGWMFQASISPVWDTGLAVLALRAAGL  Targ: PPDHPALQRAGEWLLQKQITKHGDWSFKNQSGVAGGWAFQFFNRWYPDLDDSAVVVMALDCLKLPNEDVK  1SQC: PADHDRLVKAGEWLLDRQITVPGDWAVKRPNLKPGGFAFQFDNVYYPDVDDTAVVVWALNTLRLPDERRR  Targ: NGAITRCLKWISSMQCKGGGWAAFDKDNHQHWINSTPFSDLKAMVDPSTTDISARVLEMVGRLKLHGTSF  1SQC: RDAMTKGFRWIVGMQSSNGGWGAYDVDNTSDLPNHIPFCDFGEVTDPPSEDVTAHVLECFGSFGYDDAW-  Targ: DEAHFLPPESIARGLVYLRREQENEGCWFGRWGVNYIYGTCGALVALSLVAPMTHEEEIARGARWLVQVQ  1SQC: --------KVIRRAVEYLKREQKPDGSWFGRWGVNYLYGTGAVVSALKAVGIDTREPYIQKALDWVEQHQ  Targ: NMHGKKINGPQDGGWGETCFSYNDPALKGQGDVSTASQTAWALQGLLAAGDALGKYEVESIGHGVQYLLS  1SQC: N---------PDGGWGEDCRSYEDPAYAGKG-ASTPSQTAWALMALIAGGRA----ESEAARRGVQYLVE  Targ: TQRKDGSWHESQFTGGGFPIHFYLRYHFYAQHFTLSSLARYRTRLQASKIKPPIP  1SQC: TQRPDGGWDEPYYTGTGFPGDFYLGYTMYRHVFPTLALGRYKQAIERR------- |
| 6466213 | Targ: TVSTSSAFHHSPLSDDVEPIIQKATRALLEKQQQDGHWVFELEADATIPAEYILLKHYLGEPEDLEIEAK  1SQC: -------AEQLVEAPAYARTLDRAVEYLLSCQKDEGYWWGPLLSNVTMEAEYVLLCHILDRVDRDRME-K  Targ: IGRYLRRIQGEHGGWSLFYGGDLDLSATVKAYFALKMIGDSPDAPHMLRARNEILARGGAMRANVFTRIQ  1SQC: IRRYLLHEQREDGTWALYPGGPPDLDTTIEAYVALKYIGMSRDEEPMQKALRFIQSQGGIESSRVFTRMW  Targ: LALFGAMSWEHVPQMPVELMLMPEWFPVHINKMAYWARTVLVPLLVLQALKPVARNRRGILVDELFVPDV  1SQC: LALVGEYPWEKVPMVPPEIMFLGKRMPLNIYEFGSWARATVVALSIVMSRQPVFPLPERARVPELYETD-  Targ: LPTLQESGDPIWRRFFSALDKVLHKVEPYWPKNMRAKAIHSCVHFVTERLNGEDGLGAIYPAIANSVMMY  1SQC: VPPRRRGAKGGGGWIFDALDRALHGYQKLSVHPFRRAAEIRALDWLLERQAGDGSWGGIQPPWFYALIAL  Targ: DALGYPENHPERAIARRAVEKLMVLDGTEDQGDKEVYCQPCLSPIWDTALVAHAMLEVGGDEAEKSAISA  1SQC: KILDMT-QHP---AFIKGWEG-LELY-GVELDYGGWMFQASISPVWDTGLAVLALRAAGLPADHDRLVKA  Targ: LSWLKPQQILDVKGDWAWRRPDLRPGGWAFQYRNDYYPDVDDTAVVTMAMDRAAKLSDLHDDFEESKARA  1SQC: GEWLLDRQIT-VPGDWAVKRPNLKPGGFAFQFDNVYYPDVDDTAVVVWALNTLRL--PDERRRRDAMTKG  Targ: MEWTIGMQSDNGGWGAFDANNSYTYLNNIPFADHGALLDPPTVDVSARCVSMMAQAGISITDPKMKAAVD  1SQC: FRWIVGMQSSNGGWGAYDVDNTSDLPNHIPFCDFGEVTDPPSEDVTAHVLECFGSFGYDDAWKVIRRAVE  Targ: YLLKEQEEDGSWFGRWGVNYIYGTWSALCALNVAALPHDHLAVQKAVAWLKTIQNEDGGWGENCDSYALD  1SQC: YLKREQKPDGSWFGRWGVNYLYGTGAVVSALKAVGIDTREPYIQKALDWVEQHQNPDGGWGEDCRSYE-D  Targ: YSGYEPMDSTASQTAWALLGLMAVGEANSEAVTKGINWLAQNQDEEGLWKEDYYSGGGFPRVFYLRYHGY  1SQC: PAYAGKGASTPSQTAWALMALIAGGRAESEAARRGVQYLVETQRPDGGWDEPYYTGTGFPGDFYLGYTMY  Targ: SKYFPLWALARYRNLKKANQPIVHYGM  1SQC: RHVFPTLALGRYKQAIERR-------- |
| 16519641 | Targ: NKHSGNRTAIDPAALEMSIASATEALLAYRHADGHWAFELEADSTIPSEYILLRHYLAEPIDVVLEAKIG  1SQC: -----AEQLVEAPAYARTLDRAVEYLLSCQKDEGYWWGPLLSNVTMEAEYVLLCHILDRV-DRDRMEKIR  Targ: NYLRRTQGAHGGWPLVHDGPFDMSASVKSYFALKMIGDSVDAAHMVKAREAIRARGGAANSNVLTRFLLA  1SQC: RYLLHEQREDGTWALYPGGPPDLDTTIEAYVALKYIGMSRDEEPMQKALRFIQSQGGIESSRVFTRMWLA  Targ: LYGVVSWRAVPVLPIEIVLLPIWSPFHLYKISYWARTTIVPLMVLAVLKPRAKNPKGVGIEELFLQDTKS  1SQC: LVGEYPWEKVPMVPPEIMFLGKRMPLNIYEFGSWARATVVALSIVMSRQPVFPLPERARVPELYETDVPP  Targ: VGMNPKAPHQSWGWFLLFRGIDGILRVIEPHLPKKLRERAIASALAFTEERLNGEDGMGAIYPSMANIVM  1SQC: R-----RRGAKGGGGWIFDALDRALHGYQKLSVHPFRRAAEIRALDWLLERQAGDGSWGGIQPPWFYALI  Targ: MYDALGKDDHFPPR-AIARRAIDKLLVIGEEEAYCQPC-LSPVWDTALTCHALQEVGGANAVAKAKQGLD  1SQC: ALKILDMT-QHPAFIKGWEG-LELYGVELDYGGWMFQASISPVWDTGLAVLALRAAGLPADHDRLVKAGE  Targ: WLKPRQVLDVKGDWAVKAPNIRPGGWPFQYNNAHYPDLDDTAVVVMAMDRAQRHAGSKEYATAIARGREW  1SQC: WLLDRQIT-VPGDWAVKRPNLKPGGFAFQFDNVYYPDVDDTAVVVWALNTL-RLPDERRRRDAMTKGFRW  Targ: IEGMQSRDGGWAAFDVNNLEYYLNNLPFADHGALLDPPTEDVTARCVSMLAQVGEFTQRSKAVAEGIAYL  1SQC: IVGMQSSNGGWGAYDVDNTSDLPNHIPFCDFGEVTDPPSEDVTAHVLECFGSFG-YDDAWKVIRRAVEYL  Targ: RRTQHAEGSWYGRWGLNYIYGTWSVLCALNAAGIDHQDPMIRKAVEWLVSIQSWDGGWGEDAISYRLDYS  1SQC: KREQKPDGSWFGRWGVNYLYGTGAVVSALKAVGIDTREPYIQKALDWVEQHQNPDGGWGEDCRSYE-DPA  Targ: GYEQAPSTSSQTAWALLGLMAAGEVEHPAVARGVNYLKNAQTENGLWDEQRYTATGFPRVFYLRYHGYSK  1SQC: YAGKGASTPSQTAWALMALIAGGRAESEAARRGVQYLVETQRPDGGWDEPYYTGTGFPGDFYLGYTMYRH  Targ: FFPLWALARYRNLRSTNV  1SQC: VFPTLALGRYKQAIERR- |
| 2113823 | Targ: DSVNATAREAKESKISESEILESSIASATQGVLGFQQSDGHWVFELEADCTIPAEYVLLRHYLAEPVDTV  1SQC: -----------AEQLVEAPAYARTLDRAVEYLLSCQKDEGYWWGPLLSNVTMEAEYVLLCHILDRV-DRD  Targ: LEAKIGNYLRRVQGAHGGWPLVHDGEFDMSASVKAYFALKMIGDSIDAPHMVRAREAIHARGGAIHSNVF  1SQC: RMEKIRRYLLHEQREDGTWALYPGGPPDLDTTIEAYVALKYIGMSRDEEPMQKALRFIQSQGGIESSRVF  Targ: TRFMLAMFGIVTWRAVPVLPIEIMLLPFWSPFHINKISYWARTTMVPLMVIAALKPRAKNPKGVGIDELF  1SQC: TRMWLALVGEYPWEKVPMVPPEIMFLGKRMPLNIYEFGSWARATVVALSIVMSRQPVFPLPERARVPELY  Targ: LQDPRSIGMTAKAPHQSMAWFLLFRSLDAILRVIEPLFPKSLRKRAIDTALAFSEERLNGEDGMGAIYPP  1SQC: -----ETDVPPRRRGAKGGGGWIFDALDRALHGYQKLSVHPFRRAAEIRALDWLLERQAGDGSWGGIQPP  Targ: MANLVMMYDALGKDENYPPRAVTRRGIDKLLVIGDD-EAYCQPCVSPVWDTTLTAHALLEAGGDKAGPAA  1SQC: WFYALIALKILDMT-QHPAFIKGWEGLELYGVELDYGGWMFQASISPVWDTGLAVLALRAAGLPADHDRL  Targ: KHGLDWLIPKQELEVKGDWAVKRPDVRPGGWAFQYNNAYYPDLDDTAVVVMSMDRMRREHGVTGYDSAID  1SQC: VKAGEWLLDRQIT-VPGDWAVKRPNLKPGGFAFQFDNVYYPDVDDTAVVVWALNTLRLPDERRRRD-AMT  Targ: RGREWIEGMQSDDGGWAAFDVNNLEYYLNNIPFSDHGALLDPPTEDVTARCVSMLAQLGETAKTSKHVAD  1SQC: KGFRWIVGMQSSNGGWGAYDVDNTSDLPNHIPFCDFGEVTDPPSEDVTAHVLECFGSFGYDDA-WKVIRR  Targ: GVAYLRKTQHPEGSWYGRWGMNFIYGTWSVLCALNMAGVRHDDPMIRKAADWLASIQNKDGGWGEDTVSY  1SQC: AVEYLKREQKPDGSWFGRWGVNYLYGTGAVVSALKAVGIDTREPYIQKALDWVEQHQNPDGGWGEDCRSY  Targ: RLDYKGWEAAPSTASQTAWALLALMAAGEVDHPAVARGVEYLIATQNEKGLWDEQRYTATGFPRVFYLRY  1SQC: E-DPAYAGKGASTPSQTAWALMALIAGGRAESEAARRGVQYLVETQRPDGGWDEPYYTGTGFPGDFYLGY  Targ: HGYSKFFPLWGLARYRNLRNTNSRVVGVGM  1SQC: TMYRHVFPTLALGRYKQAIERR-------- |

Table S3b. Enzymes in the 1W6K cluster

| GI | Alignment |
| --- | --- |
| 114053041 | Targ: TEGTCLRRRGGPYKTEPATDLSRWRLSNQVGRQTWTYSQEEDPVREQSGLEAHLLGLDTKSFFKDLPKAH  1W6K: TEGTCLRRRGGPYKTEPATDLGRWRLNCERGRQTWTYLQDERAGREQTGLEAYALGLDTKNYFKDLPKAH  Targ: TACRGALNGVTFYAALQTEDGHWAGDYGGPLFLLPGLLITCHVANIPLPAGYREEIIRYLRSVQLPDGGW  1W6K: TAFEGALNGMTFYVGLQAEDGHWTGDYGGPLFLLPGLLITCHVARIPLPAGYREEIVRYLRSVQLPDGGW  Targ: GLHIEDKSTVFGTALNYVSLRILGVGPDDPDLVRARNLLHKKGGAVFIPSWGKFWLAVLNVYSWEGLNTL  1W6K: GLHIEDKSTVFGTALNYVSLRILGVGPDDPDLVRARNILHKKGGAVAIPSWGKFWLAVLNVYSWEGLNTL  Targ: FPEMWLFPDWMPAHPSTIWCHCRQVYLPMAYCYSTRLSAEEGPLVQSLRQELYLEDYSCIDWAAHRNSVA  1W6K: FPEMWLFPDWAPAHPSTLWCHCRQVYLPMSYCYAVRLSAAEDPLVQSLRQELYVEDFASIDWLAQRNNVA  Targ: PDDLYTPHSWLLHVVYAILNLYERHHSTSLRQWATQKLYEHIAADDRFTKCISIGPISKTINMLVRWHVD  1W6K: PDELYTPHSWLLRVVYALLNLYEHHHSAHLRQRAVQKLYEHIVADDRFTKSISIGPISKTINMLVRWYVD  Targ: GPASAVFQEHVSRIPDYLWLGLDGMKMQGTNGSQIWDTAFAIQALLEARAQHRPEFWSCLRKAHEYLRIS  1W6K: GPASTAFQEHVSRIPDYLWMGLDGMKMQGTNGSQIWDTAFAIQALLEAGGHHRPEFSSCLQKAHEFLRLS  Targ: QVPDNFPDYQKYYRHMSKGGFSFSTLDCGWIVADCTAEALKSILLLQEKCPFVSNHVPRERLFDTVAVLL  1W6K: QVPDNPPDYQKYYRQMRKGGFSFSTLDCGWIVSDCTAEALKAVLLLQEKCPHVTEHIPRERLCDAVAVLL  Targ: SLRNPDGGFATYETKRGGHLLELLNPSEVFGDIMIDYTYVECTSAVMQALKTFHKQFPDHRAGEIRETLE  1W6K: NMRNPDGGFATYETKRGGHLLELLNPSEVFGDIMIDYTYVECTSAVMQALKYFHKRFPEHRAAEIRETLT  Targ: QGLQFCRQKQRPDGSWEGSWGVCFTYGAWFGLEAFACMGHTYHNGVACAEISRACDFLLSRQMADGGWGE  1W6K: QGLEFCRRQQRADGSWEGSWGVCFTYGTWFGLEAFACMGQTYRDGTACAEVSRACDFLLSRQMADGGWGE  Targ: DFESCKQRRYVQSAQSQIHNTCWALMGLMAVRHPDVAALERGVSYLLEKQLPNGDWPQENISGVFNKSCA  1W6K: DFESCEERRYVQSAQSQIHNTCWAMMGLMAVRHPDIEAQERGVRCLLEKQLPNGDWPQENIAGVFNKSCA  Targ: ISYTSYRNVFPIWTLGRFSRLHPDPALAGHP  1W6K: ISYTSYRNIFPIWALGRFSQLYPERALAGHP |
| 26346907 | Targ: TEGTCLRRRGGPYKTEPATDLTRWRLQNELGRQRWTYYQAEDDPGREQTGLEAHSLGLDTRSYFTDLPKA  1W6K: TEGTCLRRRGGPYKTEPATDLGRWRLNCERGRQTWTYLQ-DERAGREQTGLEAYALGLDTKNYFKDLPKA  Targ: QTAHEGALNGVTFYAKLQAEDGHWAGDYGGPLFLLPGLLITCHISHISLPAGYREEMVRYLRSVQLPDGG  1W6K: HTAFEGALNGMTFYVGLQAEDGHWTGDYGGPLFLLPGLLITCHVARIPLPAGYREEIVRYLRSVQLPDGG  Targ: WGLHIEDKSTVFGTALNYVALRILGIGPDDPDLVRARNVLHKKGGAVAIPSWGKFWLAVLNVYSWEGLNT  1W6K: WGLHIEDKSTVFGTALNYVSLRILGVGPDDPDLVRARNILHKKGGAVAIPSWGKFWLAVLNVYSWEGLNT  Targ: LFPEMWLFPEWVPAHPSTLWCHCRQVYLPMSYCYATRLSASEDPLVQSLRQELYVQDYASIDWPAQRNNV  1W6K: LFPEMWLFPDWAPAHPSTLWCHCRQVYLPMSYCYAVRLSAAEDPLVQSLRQELYVEDFASIDWLAQRNNV  Targ: SPDEMYTPHSWLLHVAYGLLNLYERFHSTSLRKWAVQMLYEHIAADDCFTKCISIGPISKTINMLVRWSV  1W6K: APDELYTPHSWLLRVVYALLNLYEHHHSAHLRQRAVQKLYEHIVADDRFTKSISIGPISKTINMLVRWYV  Targ: DGPSSPAFQEHVSRIKDYLWLGLDGMKMQGTNGSQIWDTSFAIQALLEAGAHHRPEFLPCLQKAHEFLRL  1W6K: DGPASTAFQEHVSRIPDYLWMGLDGMKMQGTNGSQIWDTAFAIQALLEAGGHHRPEFSSCLQKAHEFLRL  Targ: SQVPENCPDYQKYYRHMRKGGFSFSTLDCGWIVADCTAEGLKAVLLLQNQCPSITEHIPRERLCDAVDVL  1W6K: SQVPDNPPDYQKYYRQMRKGGFSFSTLDCGWIVSDCTAEALKAVLLLQEKCPHVTEHIPRERLCDAVAVL  Targ: LSLRNADGGFATYEKKRGGYLLELLNPSEVFGDIMIDYTYVECTSAVMQALKHFHEHFPDYRAAEVRETL  1W6K: LNMRNPDGGFATYETKRGGHLLELLNPSEVFGDIMIDYTYVECTSAVMQALKYFHKRFPEHRAAEIRETL  Targ: NQGLDFCRRKQRADGSWEGSWGVCFTYGTWFGLEAFACMGHTYQDGAACAEVAQACNFLLSQQMADGGWG  1W6K: TQGLEFCRRQQRADGSWEGSWGVCFTYGTWFGLEAFACMGQTYRDGTACAEVSRACDFLLSRQMADGGWG  Targ: EDFESCEQRRYVQSARSQVHSTCWALMGLMAVRHPDITAQERGIRCLLGKQLPNGDWPQENISGVFNKSC  1W6K: EDFESCEERRYVQSAQSQIHNTCWAMMGLMAVRHPDIEAQERGVRCLLEKQLPNGDWPQENIAGVFNKSC  Targ: AISYTSYRNIFPIWALGRFSNLYPDNTLAGHI  1W6K: AISYTSYRNIFPIWALGRFSQLYPERALAGHP |
| 13591981 | Targ: TEGTCLRRRGGPYKTEPATDLTRWRLHNELGRQRWTYYQAEEDPGREQTGLEAHSLGLDTTSYFKNLPKA  1W6K: TEGTCLRRRGGPYKTEPATDLGRWRLNCERGRQTWTYLQ-DERAGREQTGLEAYALGLDTKNYFKDLPKA  Targ: QTAHEGALNGVTFYAKLQAEDGHWAGDYGGPLFLLPGLLITCHIAHIPLPAGYREEMVRYLRSVQLPNGG  1W6K: HTAFEGALNGMTFYVGLQAEDGHWTGDYGGPLFLLPGLLITCHVARIPLPAGYREEIVRYLRSVQLPDGG  Targ: WGLHIEDKSTVFGTALSYVSLRILGIGPDDPDLVRARNILHKKGGAVAIPSWGKFWLAVLNVYSWEGINT  1W6K: WGLHIEDKSTVFGTALNYVSLRILGVGPDDPDLVRARNILHKKGGAVAIPSWGKFWLAVLNVYSWEGLNT  Targ: LFPEMWLLPEWFPAHPSTLWCHCRQVYLPMSYCYATRLSASEDPLVQSLRQELYVEDYASIDWPAQKNNV  1W6K: LFPEMWLFPDWAPAHPSTLWCHCRQVYLPMSYCYAVRLSAAEDPLVQSLRQELYVEDFASIDWLAQRNNV  Targ: CPDDMYTPHSWLLHVVYGLLNLYERFHSTSLRKWAIQLLYEHVAADDRFTKCISIGPISKTVNMLIRWSV  1W6K: APDELYTPHSWLLRVVYALLNLYEHHHSAHLRQRAVQKLYEHIVADDRFTKSISIGPISKTINMLVRWYV  Targ: DGPSSPAFQEHVSRIKDYLWLGLDGMKMQGTNGSQTWDTSFAVQALLEAGAHRRPEFLPCLQKAHEFLRL  1W6K: DGPASTAFQEHVSRIPDYLWMGLDGMKMQGTNGSQIWDTAFAIQALLEAGGHHRPEFSSCLQKAHEFLRL  Targ: SQVPDNNPDYQKYYRHMHKGGFPFSTLDCGWIVADCTAEALKAVLLLQERCPSITEHVPRERLYDAVAVL  1W6K: SQVPDNPPDYQKYYRQMRKGGFSFSTLDCGWIVSDCTAEALKAVLLLQEKCPHVTEHIPRERLCDAVAVL  Targ: LSMRNSDGGFATYETKRGGYLLELLNPSEVFGDIMIDYTYVECTSAVMQALRHFREYFPDHRATEIRETL  1W6K: LNMRNPDGGFATYETKRGGHLLELLNPSEVFGDIMIDYTYVECTSAVMQALKYFHKRFPEHRAAEIRETL  Targ: NQGLDFCRKKQRADGSWEGSWGVCFTYGTWFGLEAFACMGHIYQNRTACAEVAQACHFLLSRQMADGGWG  1W6K: TQGLEFCRRQQRADGSWEGSWGVCFTYGTWFGLEAFACMGQTYRDGTACAEVSRACDFLLSRQMADGGWG  Targ: EDFESCEQRRYVQSAGSQVHSTCWALLGLMAVRHPDISAQERGIRCLLGKQLPNGDWPQENISGVFNKSC  1W6K: EDFESCEERRYVQSAQSQIHNTCWAMMGLMAVRHPDIEAQERGVRCLLEKQLPNGDWPQENIAGVFNKSC  Targ: AISYTNYRNIFPIWALGRFSSLYPDNTLAGHI  1W6K: AISYTSYRNIFPIWALGRFSQLYPERALAGHP |
| 15076955 | Targ: -------IYGYTEKELEKTDPDGWRLIVEDTGRQRWKYLKTEEERRERPQTYMEKYFLGKNMD--LPEQP  1W6K: TEGTCLRRRGGPYKTEPATDLGRWRLNC-ERGRQTWTYLQDERAG--REQTGLEAYALGLDTKNYFKDLP  Targ: AAKTPIESARKGFSFYKHLQTSDGNWACEYGGVMFLLPGLIIAMYISKIEFPDEMRIEVIRYLVNHANPE  1W6K: KAHTAFEGALNGMTFYVGLQAEDGHWTGDYGGPLFLLPGLLITCHVARIPLPAGYREEIVRYLRSVQLP-  Targ: DGGWGIHIEGKSTVFGTALNYVVLRILGLGPDHPVTMKARIRLNELGGAIGCPQWGKFWLAVLNCYGWEG  1W6K: DGGWGLHIEDKSTVFGTALNYVSLRILGVGPDDPDLVRARNILHKKGGAVAIPSWGKFWLAVLNVYSWEG  Targ: INPILPEFWMLPEWLPIHPSRWWVHTRAVYLPMGYIYGEKFTAPVDPLIESLREELYTQPYSSINFSKHR  1W6K: LNTLFPEMWLFPDWAPAHPSTLWCHCRQVYLPMSYCYAVRLSAAEDPLVQSLRQELYVEDFASIDWLAQR  Targ: NTTSPVDVYVPHTRFLRVINSILTFYHTIFRFSWIKDMASKYAYKLIEYENKNTDFLCIGPVNFSIHILA  1W6K: NNVAPDELYTPHSWLLRVVYALLNLYEHHHSAH-LRQRAVQKLYEHIVADDRFTKSISIGPISKTINMLV  Targ: VYWKEGPDSYAFKSHKERMADFLWISKKGMMMNGTNGVQLWDTSFAVQALVESGLAEDPEFKDHMIKALD  1W6K: RWYVDGPASTAFQEHVSRIPDYLWMGLDGMKMQGTNGSQIWDTAFAIQALLEAGGHHRPEFSSCLQKAHE  Targ: FLDKCQIQKNCDDQQKCYRHRRKGAWPFSTRQQGYTVSDCTAEALKAVLLLQ-NLKSFPKRVSYDRLKDS  1W6K: FLRLSQVPDNPPDYQKYYRQMRKGGFSFSTLDCGWIVSDCTAEALKAVLLLQEKCPHVTEHIPRERLCDA  Targ: VDVILSLQNKDGGFASYELIRGPSWLEFINPAEVFGDIMIEHSYPECTTAAVTALCYFRSLCSHYRGPEI  1W6K: VAVLLNMRNPDGGFATYETKRGGHLLELLNPSEVFGDIMIDYTYVECTSAVMQALKYFHKRFPEHRAAEI  Targ: NKSVKNAIQFIKESQRPDGSWYESWAICFTYATMFALESLSCVKDFYEN---SFHSRRACDFLVNKQEED  1W6K: RETLTQGLEFCRRQQRADGSWEGSWGVCFTYGTWFGLEAFACMGQTYRDGTACAEVSRACDFLLSRQMAD  Targ: GGWSEGYQSCTDGIWTRHPTGSQVVQTAWACIGLMYANYPDETPIKRGINLIMSRQQPNGEWKQEAIEGV  1W6K: GGWGEDFESCEERRYVQS-AQSQIHNTCWAMMGLMAVRHPDIEAQERGVRCLLEKQLPNGDWPQENIAGV  Targ: FNKNCMISYPNYKFNFTIKALGMYSKRYGNI------  1W6K: FNKSCAISYTSYRNIFPIWALGRFSQLYPERALAGHP |
| 167295241 | Targ: ------------------GLPKTDPRLWRLRTDELGRESWEYLTPQQAANDPPSTFTQWLLQDPKFPQPH  1W6K: ----TEGTCLRRRGGPYKTEPATDLGRWRLN-CERGRQTWTYLQDERAG-REQTGLEAYALGLDT----K  Targ: PERNKHSPDFSAFDACHNGASFFKLLQEPDSGIFPCQYKGPMFMTIGYVAVNYIAGIEIPEHERIELIRY  1W6K: NYFKDLPKAHTAFEGALNGMTFYVGLQAE-DGHWTGDYGGPLFLLPGLLITCHVARIPLPAGYREEIVRY  Targ: IVNTAHPVDGGWGLHSVDKSTVFGTVLNYVILRLLGLPKDHPVCAKARSTLLRLGGAIGSPHWGKIWLSA  1W6K: LRSVQLP-DGGWGLHIEDKSTVFGTALNYVSLRILGVGPDDPDLVRARNILHKKGGAVAIPSWGKFWLAV  Targ: LNLYKWEGVNPAPPETWLLPYSLPMHPGRWWVHTRGVYIPVSYLSLVKFSCPMTPLLEELRNEIYTKPFD  1W6K: LNVYSWEGLNTLFPEMWLFPDWAPAHPSTLWCHCRQVYLPMSYCYAVRLSAAEDPLVQSLRQELYVEDFA  Targ: KINFSKNRNTVCGVDLYYPHSTTLNIANSLVVFYEKYLRNRFIYSLSKKKVYDLIKTELQNTDSLCIAPV  1W6K: SIDWLAQRNNVAPDELYTPHSWLLRVVYALLNLYEHH-HSAHLRQRAVQKLYEHIVADDRFTKSISIGPI  Targ: NQAFCALVTLIEEGVDSEAFQRLQYRFKDALFHGPQGMTIMGTNGVQTWDCAFAIQYFFVAGLAERPEFY  1W6K: SKTINMLVRWYVDGPASTAFQEHVSRIPDYLWMGLDGMKMQGTNGSQIWDTAFAIQALLEAGGHHRPEFS  Targ: NTIVSAYKFLCHAQFDTECVP--GSYRDKRKGAWGFSTKTQGYTVADCTAEAIKAIIMVKNSPVFSEVHH  1W6K: SCLQKAHEFLRLSQVPDNPPDYQKYYRQMRKGGFSFSTLDCGWIVSDCTAEALKAVLLLQEKCPH--VTE  Targ: MISSERLFEGIDVLLNLQNIGSFEYGSFATYEKIKAPLAMETLNPAEVFGDIMVEYPYVECTDSSVLGLT  1W6K: HIPRERLCDAVAVLLNMRNP----DGGFATYETKRGGHLLELLNPSEVFGDIMIDYTYVECTSAVMQALK  Targ: YFHKY-FDYRKEEIRTRIRIAIEFIKKSQLPDGSWYGSWGICFTYAGMFALEALHTVGETYEN---SSTV  1W6K: YFHKRFPEHRAAEIRETLTQGLEFCRRQQRADGSWEGSWGVCFTYGTWFGLEAFACMGQTYRDGTACAEV  Targ: RKGCDFLVSKQMKDGGWGESMKSSELHSYVDSEKSLVVQTAWALIALLFAEYPNKEVIDRGIDLLKNRQE  1W6K: SRACDFLLSRQMADGGWGEDFESCEERRYVQSAQSQIHNTCWAMMGLMAVRHPDIEAQERGVRCLLEKQL  Targ: ESGEWKFESVEGVFNHSCAIEYPSYRFLFPIKALGMYSRAYETHTL----  1W6K: PNGDWPQENIAGVFNKSCAISYTSYRNIFPIWALGRFSQLYPERALAGHP |
| 63054562 | Targ: ---EACRVRPELSKTVEQIDKSLWRLNIDSAGGETWEYVTKEEAEKRPLTIAEKYFLGFDLD--LPKRPP  1W6K: TEGTCLRRRGGPYKTEPATDLGRWRLNC-ERGRQTWTYLQDERAG-REQTGLEAYALGLDTKNYFKDLPK  Targ: AKTPLESAEYGYEFFRRLQLPDGHWASPYEGPMFLICGAVFAFYISQTPFPKGWAPEIIQYLINHTNDDG  1W6K: AHTAFEGALNGMTFYVGLQAEDGHWTGDYGGPLFLLPGLLITCHVARIPLPAGYREEIVRYLRSVQLPDG  Targ: GWGIHTEGVSTVFGTSMNYVVLRILGMDAGHPVATRARNRLHELGGAIGCPHWGKFWLATLNCYDWDGVN  1W6K: GWGLHIEDKSTVFGTALNYVSLRILGVGPDDPDLVRARNILHKKGGAVAIPSWGKFWLAVLNVYSWEGLN  Targ: PIPPELWLLPDWIPFHPGKWWCHVRLVYLPMGYMYGERLKCPKDSLIMQLRKELYVENYDSINFADHRNT  1W6K: TLFPEMWLFPDWAPAHPSTLWCHCRQVYLPMSYCYAVRLSAAEDPLVQSLRQELYVEDFASIDWLAQRNN  Targ: ISDVDLYFPHTQILDRLNWILEKYFTYLRPSWLKKLGTRRAYELIKIEDQNTDYSCIGPVNAAMNTVCVY  1W6K: VAPDELYTPHSWLLRVVYALLNLY-EHHHSAHLRQRAVQKLYEHIVADDRFTKSISIGPISKTINMLVRW  Targ: FHEGPSSKAFQKHIQRLHDFMWVQPEGMLMRGTNGLQVWETSFTLQALVESGLYEKEAFKPDIAKALEFL  1W6K: YVDGPASTAFQEHVSRIPDYLWMGLDGMKMQGTNGSQIWDTAFAIQALLEAGGHHRPEFSSCLQKAHEFL  Targ: DRQQIRTQYEG--SGYRYNSLGAWPFSNITQGYTVSDTTSEALRAVLLVQ-SLPDFEKLVDIPRLRLSVD  1W6K: RLSQVPDNPPDYQKYYRQMRKGGFSFSTLDCGWIVSDCTAEALKAVLLLQEKCPHVTEHIPRERLCDAVA  Targ: VILGMQNENLGFASYEPARTGEWMELLNPAEVFGNIMVEYSYPECTTSVILALRAFTKYDPGYRRDEIEN  1W6K: VLLNMRNPDGGFATYETKRGGHLLELLNPSEVFGDIMIDYTYVECTSAVMQALKYFHKRFPEHRAAEIRE  Targ: TIENALEYVVKMQRPDGSWYGSWAICFTYAAMFATGSLASAGRYYEN---CPVQKKACEFLLSKQRPDGG  1W6K: TLTQGLEFCRRQQRADGSWEGSWGVCFTYGTWFGLEAFACMGQTYRDGTACAEVSRACDFLLSRQMADGG  Targ: WSESYMACVTGVYTETESSLVTQTGWALDALINAKYPDRKPIEKGIKFLMASQKSDGSWQQKSMEGIFNK  1W6K: WGEDFESCEERRYVQSAQSQIHNTCWAMMGLMAVRHPDIEAQERGVRCLLEKQLPNGDWPQENIAGVFNK  Targ: NVAIAYPNYKLYFSIYTLGKFAKQYGNYLTI---  1W6K: SCAISYTSYRNIFPIWALGRFSQLYPERALAGHP |
| 68466833 | Targ: ----------YYSEEIGLPKTDISRWRLRSDALGRETWHYLSQSECESEPQSTFVQWLLE-SPDFPSPPS  1W6K: --TEGTCLRRRGGPYKTEPATDLGRWRLNC-ERGRQTWTYLQDERAGRE-QTGLEAYALGLDTKNYFKDL  Targ: SDIHTSGEAARKGADFLKLLQLDNGIFPCQYKGPMFMTIGYVTANYYSKTEIPEPYRVEMIRYIVNTAHP  1W6K: PKAHTAFEGALNGMTFYVGLQAEDGHWTGDYGGPLFLLPGLLITCHVARIPLPAGYREEIVRYLRSVQLP  Targ: VDGGWGLHSVDKSTCFGTTMNYVCLRLLGMEKDHPVLVKARKTLHRLGGAIKNPHWGKAWLSILNLYEWE  1W6K: -DGGWGLHIEDKSTVFGTALNYVSLRILGVGPDDPDLVRARNILHKKGGAVAIPSWGKFWLAVLNVYSWE  Targ: GVNPAPPELWRLPYWLPIHPAKWWVHTRAIYLPLGYTSANRVQCELDPLLKEIRNEIYVPSQLPYESIKF  1W6K: GLNTLFPEMWLFPDWAPAHPSTLWCHCRQVYLPMSYCYAVRLSAAEDPLVQSLRQELYVED---FASIDW  Targ: GNQRNNVCGVDLYYPHTKILDFANSILSKWEAVRPKWLLNWVNKKVYDLIVKEYQNTEYLCIAPVSFAFN  1W6K: LAQRNNVAPDELYTPHSWLLRVVYALLNLYEHHHSAHLRQRAVQKLYEHIVADDRFTKSISIGPISKTIN  Targ: MVVTCHYEGSESENFKKLQNRMNDVLFHGPQGMTVMGTNGVQVWDAAFMVQYFFMTGLVDDPKYHDMIRK  1W6K: MLVRWYVDGPASTAFQEHVSRIPDYLWMGLDGMKMQGTNGSQIWDTAFAIQALLEAGGHHRPEFSSCLQK  Targ: SYLFLVRSQFTENCVDGS--FRDRRKGAWPFSTKEQGYTVSDCTAEAMKAIIMVRNHASFADIRDEIKDE  1W6K: AHEFLRLSQVPDNPPDYQKYYRQMRKGGFSFSTLDCGWIVSDCTAEALKAVLLLQEKCP--HVTEHIPRE  Targ: NLFDAVEVLLQIQNVGEWEYGSFSTYEGIKAPLLLEKLNPAEVFNNIMVEYPYVECTDSSVLGLTYFAKY  1W6K: RLCDAVAVLLNMRNP----DGGFATYETKRGGHLLELLNPSEVFGDIMIDYTYVECTSAVMQALKYFHKR  Targ: YPDYKPELIQKTISSAIQYILDSQDNIDGSWYGCWGICYTYASMFALEALHTVGLDYES---SSAVKKGC  1W6K: FPEHRAAEIRETLTQGLEFCRR-QQRADGSWEGSWGVCFTYGTWFGLEAFACMGQTYRDGTACAEVSRAC  Targ: DFLISKQLPDGGWSESMKGCETHSYVNGENSLVVQSAWALIGLILGNYPDEEPIKRGIQFLMKRQLPTGE  1W6K: DFLLSRQMADGGWGEDFESCEERRYVQSAQSQIHNTCWAMMGLMAVRHPDIEAQERGVRCLLEKQLPNGD  Targ: WKYEDIEGVFNHSCAIEYPSYRFLFPIKALGLYKNKYGDKVLV---  1W6K: WPQENIAGVFNKSCAISYTSYRNIFPIWALGRFSQLYPERALAGHP |
| 260037884 | Targ: ----------------------------------------------------------------------  1W6K: ------TEGTCLRRRGGPYKTEPATDLGRWRLNCERGRQTWTYLQDERAGREQTGLEAYALGLDTKNYFK  Targ: ---------------------------RLINEEVVNVTLRRSLRFYSILQSQDGFWPGDYGGPLFLLPAL  1W6K: ---------------------------DLPKAHTAFEGALNGMTFYVGLQAEDGHWTGDYGGPLFLLPGL  Targ: VIGLYVTEVLDGTLTAQHQIEIRRYLYNHQNKDGGWGLHVEGNSTMFCTVLSYVALRLMGEELDGGDGAM  1W6K: LITC---HVARIPLPAGYREEIVRYLRSVQLPDGGWGLHIEDKSTVFGTALNYVSLRILGVGPD--DPDL  Targ: ESARSWIHHHGGATFIPSWGKFWLSVLGAYEWSGNNPLPPELWLLPYSLPFHPGRMWCHCRMVYLPMSYL  1W6K: VRARNILHKKGGAVAIPSWGKFWLAVLNVYSWEGLNTLFPEMWLFPDWAPAHPSTLWCHCRQVYLPMSYC  Targ: YGRRFVCRTNGTILSLRRELYTIPYHHIDWDTARNQCAKEDLYYPHPKIQDVLWSCLNKFGEPLLERWPL  1W6K: YAVRLSAAEDPLVQSLRQELYVEDFASIDWLAQRNNVAPDELYTPHSWLLRVVYALLN-----LYEHHHS  Targ: NNLRNHALQTVMQHIHYEDQNSHYICIGPVNKVLNMLCCW-VESSNSEAFKSHLSRIKDYLWVAEDGMKM  1W6K: AHLRQRAVQKLYEHIVADDRFTKSISIGPISKTINMLVRWYVDGPASTAFQEHVSRIPDYLWMGLDGMKM  Targ: QGYNGSQLWDVTLAVQAILATNLV--DDYGLMLKKAHNYIKNTQIRKDTSGDPGLWYRHPCKGGWGFSTG  1W6K: QGTNGSQIWDTAFAIQALLEAGGHHRPEFSSCLQKAHEFLRLSQVPDNPP-DYQKYYRQMRKGGFSFSTL  Targ: DNPWPVSDCTAEALKAALLLSQMPVNLVGEPMPEEHLV--------DANKNGGFASYELTRSYPELEVIN  1W6K: DCGWIVSDCTAEALKAVLLLQEKCPH-VTEHIPRERLCDAVAVLLNMRNPDGGFATYETKRGGHLLELLN  Targ: PSETFGDIIIDYQYVECTSAAIQGLVLFTTLNSSYKRKEIVGSINKAVEFIEKTQLPDGSWYGSWGVCFT  1W6K: PSEVFGDIMIDYTYVECTSAVMQALKYFHKRFPEHRAAEIRETLTQGLEFCRRQQRADGSWEGSWGVCFT  Targ: YATWFGIKGMLASGKTYES---SLCIRKACGFLLSKQLCCGGWGESYLSCQNKVYTNLPGNKSHIVNTSW  1W6K: YGTWFGLEAFACMGQTYRDGTACAEVSRACDFLLSRQMADGGWGEDFESCEERRYVQ--SAQSQIHNTCW  Targ: ALLALIEAGQASRDPMPLHRGAKSLINSQMEDGDYPQQ-VSKMF-------YIHRQVLFSLTSLIIFASY  1W6K: AMMGLMAVRHP--DIEAQERGVRCLLEKQLPNGDWPQENIAGVFNKSCAISYTSYRNIFPIWALGRFSQL  Targ: FLFRRY-------  1W6K: Y---PERALAGHP |
| 66825783 | Targ: -------------------TTTNWSLKVDRGRQTWEYSQEK---KEATDVDIHLLRLKEPGTHCPEGCDL  1W6K: TEGTCLRRRGGPYKTEPATDLGRWRLNCERGRQTWTYLQDERAGREQTGLEAYALGLDTKNYFK----DL  Targ: NRAKTPQQAIKKAFQYFSKVQTEDGHWAGDYGGPMFLLPGLVITCYVTGYQLPESTQREIIRYLFNRQNP  1W6K: PKAHTAFEGALNGMTFYVGLQAEDGHWTGDYGGPLFLLPGLLITCHVARIPLPAGYREEIVRYLRSVQLP  Targ: VDGGWGLHIEAHSDIFGTTLQYVSLRLLGVPADHPSVVKARTFLLQNGGATGIPSWGKFWLATLNAYDWN  1W6K: -DGGWGLHIEDKSTVFGTALNYVSLRILGVGPDDPDLVRARNILHKKGGAVAIPSWGKFWLAVLNVYSWE  Targ: GLNPIPIEFWLLPYNLPIAPGRWWCHCRMVYLPMSYIYAKKTTGPLTDLVKDLRREIYCQEYEKINWSEQ  1W6K: GLNTLFPEMWLFPDWAPAHPSTLWCHCRQVYLPMSYCYAVRLSAAEDPLVQSLRQELYVEDFASIDWLAQ  Targ: RNNISKLDMYYEHTSLLNVINGSLNAYEKVHSKWLRDKAIDYTFDHIRYEDEQTKYIDIGPVNKTVNMLC  1W6K: RNNVAPDELYTPHSWLLRVVYALLNLYEHHHSAHLRQRAVQKLYEHIVADDRFTKSISIGPISKTINMLV  Targ: VWDREGK-SPAFYKHADRLKDYLWLSFDGMKMQGYNGSQLWDTAFTIQAFMESG--IANQFQDCMKLAGH  1W6K: RWYVDGPASTAFQEHVSRIPDYLWMGLDGMKMQGTNGSQIWDTAFAIQALLEAGGHHRPEFSSCLQKAHE  Targ: YLDISQVPEDARDMKHYHRHYSKGAWPFSTVDHGWPISDCTAEGIKSALALRS--LPFIEPISLDRIADG  1W6K: FLRLSQVPDNPPDYQKYYRQMRKGGFSFSTLDCGWIVSDCTAEALKAVLLLQEKCPHVTEHIPRERLCDA  Targ: INVLLTLQNGDGGWASYENTRGPKWLEKFNPSEVFQNIMIDYSYVECSAACIQAMSAFRKHAPNHPRIKE  1W6K: VAVLLNMRNPDGGFATYETKRGGHLLELLNPSEVFGDIMIDYTYVECTSAVMQALKYFHKRFPEH-RAAE  Targ: INRSIARGVKFIKSIQRQDGSWLGSWGICFTYGTWFGIEGLVASGEPLT----SPSIVKACKFLASKQRA  1W6K: IRETLTQGLEFCRRQQRADGSWEGSWGVCFTYGTWFGLEAFACMGQTYRDGTACAEVSRACDFLLSRQMA  Targ: DGGWGESFKSNVTKEYVQHETSQVVNTGWALLSLMSAKYPDRECIERGIKFLIQRQYPNGDFPQESIIGV  1W6K: DGGWGEDFESCEERRYVQSAQSQIHNTCWAMMGLMAVRHPDIEAQERGVRCLLEKQLPNGDWPQENIAGV  Targ: FNFNCMISYSNYKNIFPLWALSRYNQLYLKSKI----  1W6K: FNKSCAISYTSYRNIFPIWALGRFSQLYPERALAGHP |
| 300591899 | Targ: ----------------------------------------------------------------------  1W6K: ------TEGTCLRRRGGPYKTEPATDLGRWRLNCERGRQTWTYLQDERAGREQTGLEAYALGLDTKNYFK  Targ: --------------------------EDISEDKVTVTLKRAMSFYSTLQAHDGHWPGDYGGPMFLMPGLV  1W6K: --------------------------DLPKAHTAFEGALNGMTFYVGLQAEDGHWTGDYGGPLFLLPGLL  Targ: ITLSITGVLNVVLSKEHKREICRYLYNHQNRDGGWGLHIEGPSTMFGTVLNYVTLRLLGEGANDGQGAME  1W6K: ITCHV---ARIPLPAGYREEIVRYLRSVQLPDGGWGLHIEDKSTVFGTALNYVSLRILGVGPDDPD--LV  Targ: KGRQWILDHGSATAITSWGKMWLSVLGVFEWSGNNPLPPETWLLPYILPIHPGRMWCHRRMVYLPMSYLY  1W6K: RARNILHKKGGAVAIPSWGKFWLAVLNVYSWEGLNTLFPEMWLFPDWAPAHPSTLWCHCRQVYLPMSYCY  Targ: GKRFVGPITPTVLSLRKEVFSVPYHEIDWNQARNLCAKEDLYYPHPLIQDILWASLDKVWEPIFMHWPAK  1W6K: AVRLSAAEDPLVQSLRQELYVEDFASIDWLAQRNNVAPDELYTPHSWLLRVVYALLN-----LYEHHHSA  Targ: KLREKSLRTVMEHIHYEDENTRYICIGPVNKVLNMLCCW-VEDPNSEAFKLHLPRLHDFLWLAEDGMKMQ  1W6K: HLRQRAVQKLYEHIVADDRFTKSISIGPISKTINMLVRWYVDGPASTAFQEHVSRIPDYLWMGLDGMKMQ  Targ: GYNGSQLWDTAFAVQAIISTNLA--EEYGPTLRKAHTFMKNSQVLDDCPGDLDAWYRHVSKGAWPFSTAD  1W6K: GTNGSQIWDTAFAIQALLEAGGHHRPEFSSCLQKAHEFLRLSQVPDNPP-DYQKYYRQMRKGGFSFSTLD  Targ: HGWPISDCTAEGFKAVLQLSKLPSELVGEPLDAKRLYDAVNVILSLQNSDGGYATYELTRSYSWLELVNP  1W6K: CGWIVSDCTAEALKAVLLLQEKCPH-VTEHIPRERLCDAVAVLLNMRNPDGGFATYETKRGGHLLELLNP  Targ: AETFGDIVIDYPYVECTSAAIQALTAFKKLFPGHRREEIQHSIEKAALFIEKIQSSDGSWYGSWGVCFTY  1W6K: SEVFGDIMIDYTYVECTSAVMQALKYFHKRFPEHRAAEIRETLTQGLEFCRRQQRADGSWEGSWGVCFTY  Targ: GTWFGIKGLVTAGRTFSS---CASIRKACDFLLSKQVASGGWGESYLSCQNKVYTNLEGNRSHVVNTGWA  1W6K: GTWFGLEAFACMGQTYRDGTACAEVSRACDFLLSRQMADGGWGEDFESCEERRYV--QSAQSQIHNTCWA  Targ: MLALIDAGQAERDATPLHRAAKLLINSQMENGDFPQEEIMGVFDKNCMITYAAYRNIFPIWALGEYRCRV  1W6K: MMGLMAVRHPDIEAQ--ERGVRCLLEKQLPNGDWPQENIAGVFNKSCAISYTSYRNIFPIWALGRFSQLY  Targ: LQGPS----  1W6K: PERALAGHP |
| 300592019 | Targ: ----------------------------------------------------------------------  1W6K: -------TEGTCLRRRGGPYKTEPATDLGRWRLNCERGRQTWTYLQDERAGREQTGLEAYALGLDTKNYF  Targ: -------------------------IEDVTEETVKTTLRRAINFHSTLQSHDGHWPGDYGGPMFLMPGLV  1W6K: -------------------------KDLPKAHTAFEGALNGMTFYVGLQAEDGHWTGDYGGPLFLLPGLL  Targ: ITLSITGALNAVLTEEHRKEICRYLYNHQNKDGGWGLHIEGPSTMFGSVLNYVALRLLGEGPNDRQGEME  1W6K: ITCHVAR---IPLPAGYREEIVRYLRSVQLPDGGWGLHIEDKSTVFGTALNYVSLRILGVGPDDPD--LV  Targ: KGRDWILGHGGATFITSWGKMWLSVLGVYEWSGNNPLPPEIWLLPYVLPIHPGRMWCHCRMVYLPMSYLY  1W6K: RARNILHKKGGAVAIPSWGKFWLAVLNVYSWEGLNTLFPEMWLFPDWAPAHPSTLWCHCRQVYLPMSYCY  Targ: GKRFVGPITPTILSLRKELYTIPYHDIDWNQARNLCAKEDLYYPHPLVQDILWASLHKFLEPILMHWPGK  1W6K: AVRLSAAEDPLVQSLRQELYVEDFASIDWLAQRNNVAPDELYTPHSWLLRVVYALLN-----LYEHHHSA  Targ: KLREMAIKTAIEHIHYEDDNTRYLCIGPVNKVLNMLCCW-VEDPNSEAFKLHLPRIYDYLWIAEDGMKMQ  1W6K: HLRQRAVQKLYEHIVADDRFTKSISIGPISKTINMLVRWYVDGPASTAFQEHVSRIPDYLWMGLDGMKMQ  Targ: GYNGSQLWDTAFTAQAIISSN--LIEEYGPTLRKAHTYIKNSQVLEDCPGDLSKWYRHISKGAWPFSTAD  1W6K: GTNGSQIWDTAFAIQALLEAGGHHRPEFSSCLQKAHEFLRLSQVPDNPP-DYQKYYRQMRKGGFSFSTLD  Targ: HGWPISDCTAEGLKAVLLLSKIAPEIVGEPLDAKRLYDAVNVILSLQNEDGGFATYELTRSYTWLELINP  1W6K: CGWIVSDCTAEALKAVLLLQEKCPH-VTEHIPRERLCDAVAVLLNMRNPDGGFATYETKRGGHLLELLNP  Targ: AETFGDIVIDYPYVECTSAAIQALTSFKKLYPGHRREEIQCCIEKAASFIEKTQASDGSWYGSWGVCFTY  1W6K: SEVFGDIMIDYTYVECTSAVMQALKYFHKRFPEHRAAEIRETLTQGLEFCRRQQRADGSWEGSWGVCFTY  Targ: GTWFGVKGLIAAGKSFNN---CSSIRKACEFLLSKQLPSGGWGESYLSCQNKVYSNVESNRSHVVNTGWA  1W6K: GTWFGLEAFACMGQTYRDGTACAEVSRACDFLLSRQMADGGWGEDFESCEERRYV--QSAQSQIHNTCWA  Targ: MLALIDAEQAKRDPTPLHRAAVYLINSQMENGDFPQQEIMGVFNKNCMITYAAYRNVFPIWALGEYRHRV  1W6K: MMGLMAVRHP--DIEAQERGVRCLLEKQLPNGDWPQENIAGVFNKSCAISYTSYRNIFPIWALGRFSQLY  Targ: LQSQ-----  1W6K: PERALAGHP |
| 300591983 | Targ: ----------------------------------------------------------------------  1W6K: --------------TEGTCLRRRGGPYKTEPATDLGRWRLNCERGRQTWTYLQDERAGREQTGLEAYALG  Targ: --------------------------------------EVREEAVGMTLRRAINFYSTIQADDGHWPGDY  1W6K: -----------------------------LDTKNYFKDLPKAHTAFEGALNGMTFYVGLQAEDGHWTGDY  Targ: GGPMFLIPGLVITLSITGTLNAFLSKEHQCEICRYLYNHQNEDGGWGLHIEGPSTMFGTALNYITLRLLG  1W6K: GGPLFLLPGLLITCHVARIP---LPAGYREEIVRYLRSVQLPDGGWGLHIEDKSTVFGTALNYVSLRILG  Targ: EPEDGMGAVEKARKWILDHGGATAITSWGKMWLSVLGVYEWSGNNPLPPEVWLCPYLLPCHPGRMWCHCR  1W6K: VGPDDPD-LVRARNILHKKGGAVAIPSWGKFWLAVLNVYSWEGLNTLFPEMWLFPDWAPAHPSTLWCHCR  Targ: MVYLPMSYLYGKRFVGPITSTIQSLRKELYTVPYHEIDWNKARNDCAKEDLYYPHPLVQDILWASLYYAY  1W6K: QVYLPMSYCYAVRLSAAEDPLVQSLRQELYVEDFASIDWLAQRNNVAPDELYTPHSWLLRVVYALL----  Targ: EPIFMYWPAKRLREKALDTVMQHIHYEDENTRYICIGPVNKVLNMLCCWA-EDPNSEAFKLHLPRILDYL  1W6K: -NLYEHHHSAHLRQRAVQKLYEHIVADDRFTKSISIGPISKTINMLVRWYVDGPASTAFQEHVSRIPDYL  Targ: WIAEDGMKMQGYNGSQLWDTTFAVQAIISTNIA--EEYGQTLRKAHEYIKDSQVLEDCPGDLNFWYRHIS  1W6K: WMGLDGMKMQGTNGSQIWDTAFAIQALLEAGGHHRPEFSSCLQKAHEFLRLSQVPDNPP-DYQKYYRQMR  Targ: KGAWPFSTADHGWPISDCTAEGLKAVILLSQFPSETVGKSVDVKRLYDAVHVILSLQNTDGGFATYELTR  1W6K: KGGFSFSTLDCGWIVSDCTAEALKAVLLLQEKCPH-VTEHIPRERLCDAVAVLLNMRNPDGGFATYETKR  Targ: SYHWLELINPAETFGDIVIDYPYVECTSAAIQALTLFKKLHPGHRREEIENCIAKAAEFIENIQASDGSW  1W6K: GGHLLELLNPSEVFGDIMIDYTYVECTSAVMQALKYFHKRFPEHRAAEIRETLTQGLEFCRRQQRADGSW  Targ: YGSWGVCFTYAGWFGIKGLVAAGRTYKN---CSSIHKACDYLLSKELASGGWGESYLSCQDKVYTNLKDN  1W6K: EGSWGVCFTYGTWFGLEAFACMGQTYRDGTACAEVSRACDFLLSRQMADGGWGEDFESCEERRYV--QSA  Targ: RPHIVNTGWAMLALIDAGQAERDPTPLHRAARILINSQMENGDFPQEEIMGVFNKNCMISYSAYRNIFPI  1W6K: QSQIHNTCWAMMGLMAVRHP--DIEAQERGVRCLLEKQLPNGDWPQENIAGVFNKSCAISYTSYRNIFPI  Targ: WALGEYRCRVLKAL-----  1W6K: WALGRFSQLYPERALAGHP |
| 300592007 | Targ: ----------------------------------------------------------------------  1W6K: TEGTCLRRRGGPYKTEPATDLGRWRLNCERGRQTWTYLQDERAGREQTGLEAYALGLDTKNYFK------  Targ: ------------------------EDVTEEAVSRTLRRAINFYSTIQGDDGHWPGDYGGPMFLIPGLVIT  1W6K: ------------------------DLPKAHTAFEGALNGMTFYVGLQAEDGHWTGDYGGPLFLLPGLLIT  Targ: LSITGALNAVLSTEHQREICRYLYNHQNKDGGWGLHIEGPSTMFGSVLNYVSLRLLGEEAEDGQGAVDKA  1W6K: CHVA---RIPLPAGYREEIVRYLRSVQLPDGGWGLHIEDKSTVFGTALNYVSLRILGVGPDDPD--LVRA  Targ: RKWILDHGGASAITSWGKMWLSVLGVYEWAGNNPLPPELWLLPYLLPFHPGRMWCHCRMVYLPMCYLYGK  1W6K: RNILHKKGGAVAIPSWGKFWLAVLNVYSWEGLNTLFPEMWLFPDWAPAHPSTLWCHCRQVYLPMSYCYAV  Targ: RFVGPITPIIRSLRKELYLVPYHEVDWNKARNECAKEDLYYPHPLVQDIVWASLHHVYEPLFMRWPAKRL  1W6K: RLSAAEDPLVQSLRQELYVEDFASIDWLAQRNNVAPDELYTPHSWLLRVVYALLN-----LYEHHHSAHL  Targ: REKALQCVMQHIHYEDENTRYICIGPVNKVLNMLCCWV-EDPHSEAFKLHIPRIFDYLWIAEDGMKMQGY  1W6K: RQRAVQKLYEHIVADDRFTKSISIGPISKTINMLVRWYVDGPASTAFQEHVSRIPDYLWMGLDGMKMQGT  Targ: NGSQLWDTAFAVQAIMSTKLA--EEYGTTLRKAHKYIKDSQVLEDCPGDLQSWYRHISKGAWPFSTADHG  1W6K: NGSQIWDTAFAIQALLEAGGHHRPEFSSCLQKAHEFLRLSQVPDNPP-DYQKYYRQMRKGGFSFSTLDCG  Targ: WPISDCTAEGLKAVLLLSKLPSEIVGKSIDEEQIYDAVNVILSLQNTDGGFATYELTRSYPWLELMNPAE  1W6K: WIVSDCTAEALKAVLLLQEKCPH-VTEHIPRERLCDAVAVLLNMRNPDGGFATYETKRGGHLLELLNPSE  Targ: TFGDIVIDYTYVECTSAAIQALVAFKKLYPGHRRDEIDNCVAKAADFIESIQATDGSWYGSWGVCFTYGG  1W6K: VFGDIMIDYTYVECTSAVMQALKYFHKRFPEHRAAEIRETLTQGLEFCRRQQRADGSWEGSWGVCFTYGT  Targ: WFGIRGLVAAGRRYDN---CSSLRKACDFLLSKELASGGWGESYLSGQNKVYTNIKDDRPHIVNTGWAML  1W6K: WFGLEAFACMGQTYRDGTACAEVSRACDFLLSRQMADGGWGEDFESCEERRYV--QSAQSQIHNTCWAMM  Targ: SLIDAGQSERDPTPLHRAARILINSQMDDGDFPQEEIMGIFNKNCMISYAAYRNIFPIWALGEYRCRVLQ  1W6K: GLMAVRHP--DIEAQERGVRCLLEKQLPNGDWPQENIAGVFNKSCAISYTSYRNIFPIWALGRFSQLYPE  Targ: AP-----  1W6K: RALAGHP |
| 82468805 | Targ: ----------------------------------------------------------------------  1W6K: ----TEGTCLRRRGGPYKTEPATDLGRWRLNCERGRQTWTYLQDE-RAGREQTGLEAYALGLDTKNYFKD  Targ: -----------------------------QVTEEKVKITLRRALNYYSSIQADDGHWPGDYGGPMFLMPG  1W6K: -----------------------------LPKAHTAFEGALNGMTFYVGLQAEDGHWTGDYGGPLFLLPG  Targ: LIIALSITGALNAILSEEHKREMCRYLYNHQNRDGGWGLHIEGPSTMFGSVLCYVSLRLLGEGPNEGEGA  1W6K: LLITCHV---ARIPLPAGYREEIVRYLRSVQLPDGGWGLHIEDKSTVFGTALNYVSLRILGVGPDDPD--  Targ: VERGRNWILKHGGATAITSWGKMWLSVLGAYEWSGNNPLPPEMWLLPYILPVHPGRMWCHCRMVYLPMSY  1W6K: LVRARNILHKKGGAVAIPSWGKFWLAVLNVYSWEGLNTLFPEMWLFPDWAPAHPSTLWCHCRQVYLPMSY  Targ: LYGKRFVGPITPTVLSLRKELYTVPYHEIDWNQARNQCAKEDLYYPHPMLQDVLWATLHKFVEPILMHWP  1W6K: CYAVRLSAAEDPLVQSLRQELYVEDFASIDWLAQRNNVAPDELYTPHSWLLRVVYALLN-----LYEHHH  Targ: GKRLREKAIQTAIEHIHYEDENTRYICIGPVNKVLNMLCCW-VEDPNSEAFKLHLPRLYDYLWLAEDGMK  1W6K: SAHLRQRAVQKLYEHIVADDRFTKSISIGPISKTINMLVRWYVDGPASTAFQEHVSRIPDYLWMGLDGMK  Targ: MQGYNGSQLWDTAFAVQAIVSTNL--IEEYGPTLKKAHSFIKKMQVLENCPGDLNFWYRHISKGAWPFST  1W6K: MQGTNGSQIWDTAFAIQALLEAGGHHRPEFSSCLQKAHEFLRLSQVPDNPP-DYQKYYRQMRKGGFSFST  Targ: ADHGWPISDCTAEGIKALMLLSKIPSEIVGEGLNANRLYDAVNVVLSLQNGDGGFPTYELSRSYSWLEFI  1W6K: LDCGWIVSDCTAEALKAVLLLQEKCPH-VTEHIPRERLCDAVAVLLNMRNPDGGFATYETKRGGHLLELL  Targ: NPAETFGDIVIDYPYVECTSAAIQALTSFRKSYPEHQREEIECCIKKAAKFMEKIQISDGSWYGSWGVCF  1W6K: NPSEVFGDIMIDYTYVECTSAVMQALKYFHKRFPEHRAAEIRETLTQGLEFCRRQQRADGSWEGSWGVCF  Targ: TYGTWFGIKGLVAAGKSFGN---CSSIRKACDFLLSKQCPSGGWGESYLSCQKKVYSNLEGDRSHVVNTA  1W6K: TYGTWFGLEAFACMGQTYRDGTACAEVSRACDFLLSRQMADGGWGEDFESCEERRYV--QSAQSQIHNTC  Targ: WAMLSLIDAGQAERDPTPLHRAARYLINAQMENGDFPQQEIMGVFNRNCMITYAAYRDIFPIWALGEYRC  1W6K: WAMMGLMAVRHP--DIEAQERGVRCLLEKQLPNGDWPQENIAGVFNKSCAISYTSYRNIFPIWALGRFSQ  Targ: RVLKAS-----  1W6K: LYPERALAGHP |
| 452446 | Targ: ----------------------------------------------------------------------  1W6K: --------TEGTCLRRRGGPYKTEPATDLGRWRLNCERGRQTWTYLQDERAGREQTGLEAYALGLDTKNY  Targ: ---------------------------------VETTLKRGLDFYSTIQAHDGHWPGDYGGPMFLLPGLI  1W6K: ------------------------FKDLPKAHTAFEGALNGMTFYVGLQAEDGHWTGDYGGPLFLLPGLL  Targ: ITLSITGALNTVLSEQHKQEMRRYLYNHQNEDGGWGLHIEGPSTMFGSVLNYVTLRLLGEGPNDGDGDME  1W6K: ITCHVA---RIPLPAGYREEIVRYLRSVQLPDGGWGLHIEDKSTVFGTALNYVSLRILGVGPDDPD--LV  Targ: KGRDWILNHGGATNITSWGKMWLSVLGAFEWSGNNPLPPEIWLLPYFLPIHPGRMWCHCRMVYLPMSYLY  1W6K: RARNILHKKGGAVAIPSWGKFWLAVLNVYSWEGLNTLFPEMWLFPDWAPAHPSTLWCHCRQVYLPMSYCY  Targ: GKRFVGPITSTVLSLRKELFTVPYHEVNWNEARNLCAKEDLYYPHPLVQDILWASLHKIVEPVLMRWPGA  1W6K: AVRLSAAEDPLVQSLRQELYVEDFASIDWLAQRNNVAPDELYTPHSWLLRVVYALLN-----LYEHHHSA  Targ: NLREKAIRTAIEHIHYEDENTRYICIGPVNKVLNMLCCW-VEDPNSEAFKLHLPRIHDFLWLAEDGMKMQ  1W6K: HLRQRAVQKLYEHIVADDRFTKSISIGPISKTINMLVRWYVDGPASTAFQEHVSRIPDYLWMGLDGMKMQ  Targ: GYNGSQLWDTGFAIQAILATN--LVEEYGPVLEKAHSFVKNSQVLEDCPGDLNYWYRHISKGAWPFSTAD  1W6K: GTNGSQIWDTAFAIQALLEAGGHHRPEFSSCLQKAHEFLRLSQVPDNPP-DYQKYYRQMRKGGFSFSTLD  Targ: HGWPISDCTAEGLKAALLLSKVPKEIVGEPIDAKRLYEAVNVIISLQNADGGLATYELTRSYPWLELINP  1W6K: CGWIVSDCTAEALKAVLLLQEKCPH-VTEHIPRERLCDAVAVLLNMRNPDGGFATYETKRGGHLLELLNP  Targ: AETFGDIVIDYPYVECTSAAIQALISFRKLYPGHRKKEVDECIEKAVKFIESIQAADGSWYGSWAVCFTY  1W6K: SEVFGDIMIDYTYVECTSAVMQALKYFHKRFPEHRAAEIRETLTQGLEFCRRQQRADGSWEGSWGVCFTY  Targ: GTWFGVKGLVAVGKTLKN---SPHVAKACEFLLSKQQPSGGWGESYLSCQDKVYSNLDGNRSHVVNTAWA  1W6K: GTWFGLEAFACMGQTYRDGTACAEVSRACDFLLSRQMADGGWGEDFESCEERRYV--QSAQSQIHNTCWA  Targ: MLALIGAGQAEVDRKPLHRAARYLINAQMENGDFPQQEIMGVFNRNCMITYAAYRNIFPIWALGEYRCQV  1W6K: MMGLMAVRHP--DIEAQERGVRCLLEKQLPNGDWPQENIAGVFNKSCAISYTSYRNIFPIWALGRFSQLY  Targ: LLQQGE---  1W6K: PERALAGHP |
| 300591913 | Targ: ----------------------------------------------------------------------  1W6K: ------TEGTCLRRRGGPYKTEPATDLGRWRLNCERGRQTWTYLQDERAGREQTGLEAYALGLDTKNYFK  Targ: -----------------------------EDVTEEAVTRTLRRAINFYSTIQADDGHWPGDYGGPMFLIP  1W6K: -----------------------------DLPKAHTAFEGALNGMTFYVGLQAEDGHWTGDYGGPLFLLP  Targ: GLVITLSITGALNAVLSTEHQREICRYLYNHQNKDGGWGLHIEGPSTMFGSVLNYVTLRLLGEEAEDGQG  1W6K: GLLITCHV---ARIPLPAGYREEIVRYLRSVQLPDGGWGLHIEDKSTVFGTALNYVSLRILGVGPDDPD-  Targ: AVDKARKWILDHGGAAAITSWGKMWLSVLGVYEWAGNNPLPPELWLLPYLLPCHPGRMWCHCRMVYLPMC  1W6K: -LVRARNILHKKGGAVAIPSWGKFWLAVLNVYSWEGLNTLFPEMWLFPDWAPAHPSTLWCHCRQVYLPMS  Targ: YLYGKRFVGPITPIIRSLRKELYLVPYHEVDWNKARNQCAKEDLYYPHPLVQDILWATLHHVYEPLFMHW  1W6K: YCYAVRLSAAEDPLVQSLRQELYVEDFASIDWLAQRNNVAPDELYTPHSWLLRVVYALLN-----LYEHH  Targ: PAKRLREKALQSVMQHIHYEDENTRYICIGPVNKVLNMLCCWAED-PHSEAFKLHIPRIYDYLWIAEDGM  1W6K: HSAHLRQRAVQKLYEHIVADDRFTKSISIGPISKTINMLVRWYVDGPASTAFQEHVSRIPDYLWMGLDGM  Targ: KMQGYNGSQLWDTAFAVQAIISTE--LAEEYETTLRKAHKYIKDSQVLEDCPGDLQSWYRHISKGAWPFS  1W6K: KMQGTNGSQIWDTAFAIQALLEAGGHHRPEFSSCLQKAHEFLRLSQVPDNPP-DYQKYYRQMRKGGFSFS  Targ: TADHGWPISDCTAEGLKAVLLLSKLPSEIVGKSIDEQQLYNAVNVILSLQNTDGGFATYELTRSYRWLEL  1W6K: TLDCGWIVSDCTAEALKAVLLLQEKCPH-VTEHIPRERLCDAVAVLLNMRNPDGGFATYETKRGGHLLEL  Targ: MNPAETFGDIVIDYPYVECSSAAIQALAAFKKLYPGHRRDEIDNCIAEAADFIESIQATDGSWYGSWGVC  1W6K: LNPSEVFGDIMIDYTYVECTSAVMQALKYFHKRFPEHRAAEIRETLTQGLEFCRRQQRADGSWEGSWGVC  Targ: FTYGGWFGIRGLVAAGRRYNN---CSSLRKACDFLLSKELAAGGWGESYLSCQNKVYTNIKDDRPHIVNT  1W6K: FTYGTWFGLEAFACMGQTYRDGTACAEVSRACDFLLSRQMADGGWGEDFESCEERRYV--QSAQSQIHNT  Targ: GWAMLSLIDAGQSERDPTPLHRAARVLINSQMEDGDFPQEEIMGVFNKNCMISYSAYRNIFPIWALGEYR  1W6K: CWAMMGLMAVRHP--DIEAQERGVRCLLEKQLPNGDWPQENIAGVFNKSCAISYTSYRNIFPIWALGRFS  Targ: SRVLKPLK----  1W6K: QLYPERALAGHP |
| 300807982 | Targ: ----------------------------------------------------------------------  1W6K: -------TEGTCLRRRGGPYKTEPATDLGRWRLNCERGRQTWTYLQDERAGREQTGLEAYALGLDTKNYF  Targ: --------------------------IEDITEDAVTNTLRRAINFHSTTQAHDGHWPGDYGGPLFLMPGL  1W6K: --------------------------KDLPKAHTAFEGALNGMTFYVGLQAEDGHWTGDYGGPLFLLPGL  Targ: VITLSITGALNAVLSKEHKKEMCRYLYNHQNEDGGWGLHIEGPSTMFGSVLNYVTLRLLGEDVNGGDGEI  1W6K: LITCHV---ARIPLPAGYREEIVRYLRSVQLPDGGWGLHIEDKSTVFGTALNYVSLRILGVGPD--DPDL  Targ: ERARKWILDHGGATAITSWGKMWLSVLGVFEWCGNNPLPPEMWLFPYYLPVHPGRMWCHCRMVYLPMSYL  1W6K: VRARNILHKKGGAVAIPSWGKFWLAVLNVYSWEGLNTLFPEMWLFPDWAPAHPSTLWCHCRQVYLPMSYC  Targ: YGKRFVGPITPTVLSLRKELFTVPYHEIDWNEARSLCAKEDLYYPHPVVQDILWATLHKVVEPVLLNWPG  1W6K: YAVRLSAAEDPLVQSLRQELYVEDFASIDWLAQRNNVAPDELYTPHSWLLRVVYALLN-----LYEHHHS  Targ: KKLREKALCSAIEHIHYEDENTRYICIGPVNKVLNMLCCW-VEDPNSEAFKLHIPRLYDYLWIAEDGMKM  1W6K: AHLRQRAVQKLYEHIVADDRFTKSISIGPISKTINMLVRWYVDGPASTAFQEHVSRIPDYLWMGLDGMKM  Targ: QGYNGSQLWDTAFSVQAIVATK--LVEEFSSTISKAHEFMKNSQVLEDYPGDLSYWYRHISKGAWPFSTA  1W6K: QGTNGSQIWDTAFAIQALLEAGGHHRPEFSSCLQKAHEFLRLSQVPDNPP-DYQKYYRQMRKGGFSFSTL  Targ: DHGWPISDCTAEGLKVVLKLSQFPAELVGAPLSAKLVYNAVNVILSLQNIDGGFATYELTRSYSWMELLN  1W6K: DCGWIVSDCTAEALKAVLLLQEKCPH-VTEHIPRERLCDAVAVLLNMRNPDGGFATYETKRGGHLLELLN  Targ: PAETFGDIVIDYPYVECTSAALQSLVLFKKLHPEHRKEEVELCIKKAAAFIEKIQESDGSWYGSWAVCFT  1W6K: PSEVFGDIMIDYTYVECTSAVMQALKYFHKRFPEHRAAEIRETLTQGLEFCRRQQRADGSWEGSWGVCFT  Targ: YGTWFGVLGLVAAGRNYKN---SPSIRKACDFLLSKQLASGGWGESYLSCQNKVYTNIPGGRSHVVNTGW  1W6K: YGTWFGLEAFACMGQTYRDGTACAEVSRACDFLLSRQMADGGWGEDFESCEERRYVQ--SAQSQIHNTCW  Targ: AMLALIGAGQAERDPVPLHRAAKFLIESQLENGDFPQQEIMGVFNKNCMISYAAYRNIFPIWALGEYRCK  1W6K: AMMGLMAVRHP--DIEAQERGVRCLLEKQLPNGDWPQENIAGVFNKSCAISYTSYRNIFPIWALGRFSQL  Targ: VLNASRGQMKT  1W6K: YPERALAGHP- |
| 300591981 | Targ: ----------------------------------------------------------------------  1W6K: ------TEGTCLRRRGGPYKTEPATDLGRWRLNCERGRQTWTYLQDERAGREQTGLEAYALGLDTKNYFK  Targ: --------------------------EDVTEEMVTRMLRRAISFHSTLQAHDGHWAGDYGGPMFLMPGLV  1W6K: --------------------------DLPKAHTAFEGALNGMTFYVGLQAEDGHWTGDYGGPLFLLPGLL  Targ: ITLSITGALNTVLSEEHKKEMCRYLYNHQNKDGGWGLHIEGPSTMFGTVLSYVTLRLLGEGANDGQGAIE  1W6K: ITCHVA---RIPLPAGYREEIVRYLRSVQLPDGGWGLHIEDKSTVFGTALNYVSLRILGVGPDDPD--LV  Targ: RGRKWILDHGSATAIISWGKMWLSVLGAFEWSGNNPLPPEIWLLPYMLPVHPGRMWCHCRMVYLPMSYLY  1W6K: RARNILHKKGGAVAIPSWGKFWLAVLNVYSWEGLNTLFPEMWLFPDWAPAHPSTLWCHCRQVYLPMSYCY  Targ: GKRFVGPITPTVMSLRKELYSVPYHEIDWNQARNLCAKEXLYYPHPLVQDILWASLHKLVEPVLMRWPGK  1W6K: AVRLSAAEDPLVQSLRQELYVEDFASIDWLAQRNNVAPDELYTPHSWLLRVVYALLN-----LYEHHHSA  Targ: RLREKALRTVLEHIHYEDENTRYICIGPVNKVLNMLCCW-VEDPNSEAFKLHLPRINDYLWIAEDGMKMQ  1W6K: HLRQRAVQKLYEHIVADDRFTKSISIGPISKTINMLVRWYVDGPASTAFQEHVSRIPDYLWMGLDGMKMQ  Targ: GYNGSQLWDTAFAVQAIISTNLF--EEYGPTLEKAHMYIKKSQVREDCPGDLDFWYRHISKGAWPFSTAD  1W6K: GTNGSQIWDTAFAIQALLEAGGHHRPEFSSCLQKAHEFLRLSQVPDNPP-DYQKYYRQMRKGGFSFSTLD  Targ: HGWPISDCTAEGLKAALLLSKIPPDVVGEPLVEERLYDAVNVILSLQNADGGFATYELTRSYPWLELINP  1W6K: CGWIVSDCTAEALKAVLLLQEKCPH-VTEHIPRERLCDAVAVLLNMRNPDGGFATYETKRGGHLLELLNP  Targ: AETFGDIVIDYNYVECTSAAIQALTSFKKSYPKHREEEVDVCIKRAAMFTEKIQASDGSWYGSWGVCFTY  1W6K: SEVFGDIMIDYTYVECTSAVMQALKYFHKRFPEHRAAEIRETLTQGLEFCRRQQRADGSWEGSWGVCFTY  Targ: GTWFGVKGLVAAGKNFND---CFGIRKACDFLLSKQLPSGGWGESYLSCQNKVYSHVEGNRSHVVNTGWA  1W6K: GTWFGLEAFACMGQTYRDGTACAEVSRACDFLLSRQMADGGWGEDFESCEERRYV--QSAQSQIHNTCWA  Targ: MLALIEAGQAERDPTPLHRAARVLINSQMENGDFPQEEIMGVFNRNCMITYAAYRNIFPIWALGEYRCRV  1W6K: MMGLMAVRHP--DIEAQERGVRCLLEKQLPNGDWPQENIAGVFNKSCAISYTSYRNIFPIWALGRFSQLY  Targ: LQA-----P  1W6K: PERALAGHP |
| 6090879 | Targ: ----------------------------------------------------------------------  1W6K: ------------TEGTCLRRRGGPYKTE-PATDLGRWRLNCERGRQTWTYLQDERAGREQTGLEAYALGL  Targ: ------------------------HEAVTGEAVLSSLKRAIARYSTFQAHDGHWPGDYGGPMFLMPGLII  1W6K: ------------------DTKNYFKDLPKAHTAFEGALNGMTFYVGLQAEDGHWTGDYGGPLFLLPGLLI  Targ: TLYVSGALNTALSSEHQKEIRRYLYNHQNEDGGWGLHIEGHSTMFGSALTYVSLRLLGEGPDSGDGAMEK  1W6K: TCHVA---RIPLPAGYREEIVRYLRSVQLPDGGWGLHIEDKSTVFGTALNYVSLRILGVGPDDPD--LVR  Targ: GRKWILDHGGATYITSWGKFWLSVLGVFDWSGNNPVPPEIWLLPYFLPIHPGRMWCHCRMVYLPMCYIYG  1W6K: ARNILHKKGGAVAIPSWGKFWLAVLNVYSWEGLNTLFPEMWLFPDWAPAHPSTLWCHCRQVYLPMSYCYA  Targ: KRFVGPVTPIILELRKELYEVPYNEVDWDKARNLCAKEDLYYPHPFVQDVLWATLHKFVEPAMLRWPGNK  1W6K: VRLSAAEDPLVQSLRQELYVEDFASIDWLAQRNNVAPDELYTPHSWLLRVVYALLNLYE-----HHHSAH  Targ: LREKALDTVMQHIHYEDENTRYICIGPVNKVLNMLACWI-EDPNSEAFKLHIPRVHDYLWIAEDGMKMQG  1W6K: LRQRAVQKLYEHIVADDRFTKSISIGPISKTINMLVRWYVDGPASTAFQEHVSRIPDYLWMGLDGMKMQG  Targ: YNGSQLWDTAFTVQAIVATGLI--EEFGPTLKLAHGYIKKTQVIDDCPGDLSQWYRHISKGAWPFSTADH  1W6K: TNGSQIWDTAFAIQALLEAGGHHRPEFSSCLQKAHEFLRLSQVPDNPP-DYQKYYRQMRKGGFSFSTLDC  Targ: GWPISDCTAEGLKAALLLSKISPDIVGEAVEVNRLYDSVNCLMSYMNDNGGFATYELTRSYAWLELINPA  1W6K: GWIVSDCTAEALKAVLLLQEKCPH-VTEHIPRERLCDAVAVLLNMRNPDGGFATYETKRGGHLLELLNPS  Targ: ETFGDIVIDYPYVECTSAAIQALTAFKKLYPGHRKSEIDNCISKAASFIEGIQKSDGSWYGSWAVCFTYG  1W6K: EVFGDIMIDYTYVECTSAVMQALKYFHKRFPEHRAAEIRETLTQGLEFCRRQQRADGSWEGSWGVCFTYG  Targ: TWFGVKGLVAAGRTFKN---SPAIRKACDFLLSKELPSGGWGESYLSSQDQVYTNLEGKRPHAVNTGWAM  1W6K: TWFGLEAFACMGQTYRDGTACAEVSRACDFLLSRQMADGGWGEDFESCEERRYVQS--AQSQIHNTCWAM  Targ: LALIDAGQAERDPIPLHRAAKVLINLQSEDGEFPQQEIIGVFNKNCMISYSEYRNIFPIWALGEYRRRV-  1W6K: MGLMAVRHP--DIEAQERGVRCLLEKQLPNGDWPQENIAGVFNKSCAISYTSYRNIFPIWALGRFSQLYP  Targ: ---LAADK  1W6K: ERALAGHP |
| 119499584 | Targ: TTDSSMPATVIGKAEFSNTKAASEFGTNLSRWRLNVDNGRHMWEYLESEDEARKRPQSILEKYWLGLPYE  1W6K: --------TEGTCLRRRGGPYKTEPATDLGRWRLNCERGRQTWTYLQDERAGREQTGLEAYALGLDTKNY  Targ: LPARPRATRALEAVENGWEFFKRLQTADGHWGCNDDGPLFVTSGMVIARYIVGIPIDSHMKQEMCRYLLN  1W6K: FKDLPKAHTAFEGALNGMTFYVGLQAEDGHWTGDYGGPLFLLPGLLITCHVARIPLPAGYREEIVRYLRS  Targ: VVNEDGGWGLFIQSPSTVFGTVMNYCMLRILGLGPEHPAMARARNTLHRLGSASATPTWGKFWLCVLGVY  1W6K: VQLPDGGWGLHIEDKSTVFGTALNYVSLRILGVGPDDPDLVRARNILHKKGGAVAIPSWGKFWLAVLNVY  Targ: EWEGMIPLPPEPLLVPASLPFNPGKWWVHTRNVYISMSYLYGHRFSMPPNKLVQALRDELYDIPYEQINW  1W6K: SWEGLNTLFPEMWLFPDWAPAHPSTLWCHCRQVYLPMSYCYAVRLSAAEDPLVQSLRQELYVEDFASIDW  Targ: PAQRTNVSAADRLTDPTWIQRSFTSALTTYETFKIPFLRRRALNEALFQIETETRNTHYLCIAPVSFASN  1W6K: LAQRNNVAPDELYTPHSWLLRVVYALLNLYEHHHSAHLRQRAVQKLYEHIVADDRFTKSISIGPISKTIN  Targ: MLALYHAHGRDSHWIRGMRDRFIDPMWLCREGLAASGTNGTSLWDTALTVQATIDAGLAARPENQAILRK  1W6K: MLVRWYVDGPASTAFQEHVSRIPDYLWMGLDGMKMQGTNGSQIWDTAFAIQALLEAGGHHRPEFSSCLQK  Targ: ALEFIDNSQIREDPLGVHHVYRQPTRGAWPFSTRDQSYAVSDTTAEAVKVVVLLQ-QIEGFPSRISDERL  1W6K: AHEFLRLSQVPDNPPDYQKYYRQMRKGGFSFSTLDCGWIVSDCTAEALKAVLLLQEKCPHVTEHIPRERL  Targ: QQAIDLILGMENAGGGFSAYEPVRGPKFLELLNITELYENVMTDNLYPECTSSVIMSLTTFAREYPTYRA  1W6K: CDAVAVLLNMRNPDGGFATYETKRGGHLLELLNPSEVFGDIMIDYTYVECTSAVMQALKYFHKRFPEHRA  Targ: RDIQACVSRSVDYLLRSQYPNGGWFASWGVCFTYATMFALQGLACMGRNESN---CAACQRACSFLLQHQ  1W6K: AEIRETLTQGLEFCRRQQRADGSWEGSWGVCFTYGTWFGLEAFACMGQTYRDGTACAEVSRACDFLLSRQ  Targ: NPDGGWGESLDTVRFKQYLPHPDGSQVTNTAYAVIGLLAARCGNHEAIRRGVAYLMKEQQDTGEWLPGAL  1W6K: MADGGWGEDFESCEERRYV-QSAQSQIHNTCWAMMGLMAVRHPDIEAQERGVRCLLEKQLPNGDWPQENI  Targ: EGVFAPPGGMRYPNYKFHFTLMALGRYAAVHGDECLAAQLV  1W6K: AGVFNKSCAISYTSYRNIFPIWALGRFSQLYPERALAGHP- |
| 70993016 | Targ: ATDSSMPGTVIGKAEFSDTKAASEFGTDLSRWRLNVDNGRHMWEYLESEDEARKRPQSFLEKYWLGLPYE  1W6K: ---TEGTCLRRRG-----GPYKTEPATDLGRWRLNCERGRQTWTYLQDERAG--REQTGLEAYALGLDTK  Targ: --LPARPRATCALEAVENGWEFFKRLQTADGHWGCNDDGPLFVTSGMVIARYIVGIPIDSHMKQEMCRYL  1W6K: NYFKDLPKAHTAFEGALNGMTFYVGLQAEDGHWTGDYGGPLFLLPGLLITCHVARIPLPAGYREEIVRYL  Targ: LNVVNEDGGWGLFIQSPSTVFGTVMNYCMLRILGLGPEHPAMAKARNTLHRLGSARATPTWGKFWLCVLG  1W6K: RSVQLPDGGWGLHIEDKSTVFGTALNYVSLRILGVGPDDPDLVRARNILHKKGGAVAIPSWGKFWLAVLN  Targ: VYEWEGMVPLPPEPLLVPASLPFNPGKWWVHTRNVYISMSYLYGHRFSMPPNKLVQALRDELYDIPYQQI  1W6K: VYSWEGLNTLFPEMWLFPDWAPAHPSTLWCHCRQVYLPMSYCYAVRLSAAEDPLVQSLRQELYVEDFASI  Targ: NWPAQRTNVSAADRLTDPTWIQRSFTSALTMYETFKIPFLRRRALNEALFQIETETRNTHYLCIAPVSFA  1W6K: DWLAQRNNVAPDELYTPHSWLLRVVYALLNLYEHHHSAHLRQRAVQKLYEHIVADDRFTKSISIGPISKT  Targ: SNMLALYHAHGRDSHWIRGMRDRFIDPMWLCREGLAASGTNGTSLWDTALTVQATIDAGLAARPENQAIL  1W6K: INMLVRWYVDGPASTAFQEHVSRIPDYLWMGLDGMKMQGTNGSQIWDTAFAIQALLEAGGHHRPEFSSCL  Targ: RKALEFIDNSQIREDPLGVHHVYRQPTRGAWPFSTRDQSYAVSDTTAEAVKVIVLLQ-RIEGFPSRISDE  1W6K: QKAHEFLRLSQVPDNPPDYQKYYRQMRKGGFSFSTLDCGWIVSDCTAEALKAVLLLQEKCPHVTEHIPRE  Targ: RLQQAIDLILGMENAGGGFSAYEPVRGPKFLELLNITELYENVMTDNLYPECTSSVIMCLTTFAREYPTY  1W6K: RLCDAVAVLLNMRNPDGGFATYETKRGGHLLELLNPSEVFGDIMIDYTYVECTSAVMQALKYFHKRFPEH  Targ: RPRDIQACLSRSIDYLLRSQYPNGGWFASWGVCFTYATMFALQGLACMGWNESN---CAACQRACSFLLQ  1W6K: RAAEIRETLTQGLEFCRRQQRADGSWEGSWGVCFTYGTWFGLEAFACMGQTYRDGTACAEVSRACDFLLS  Targ: HQNPDGGWGESLDTVRFKQYLPHPDGSQVTNTAYAVIGLLAARCGNHEAIRRGVAYLVKEQQDTGEWLPG  1W6K: RQMADGGWGEDFESCEERRYV-QSAQSQIHNTCWAMMGLMAVRHPDIEAQERGVRCLLEKQLPNGDWPQE  Targ: PLEGVFAPPGGMRYPNYKFHFTLMALGRYVAIHGNECLAI--  1W6K: NIAGVFNKSCAISYTSYRNIFPIWALGRFSQLYPERALAGHP |
| 300591911 | Targ: ----------------------------------------------------------------------  1W6K: ---------TEGTCLRRRGGPYKTEPATDLGRWRLNCERGRQTWTYLQDERAGREQTGLEAYALGLDTKN  Targ: -------------------------------------KEAVKSTLERALGFYSAVQTRDGNWASDLGGPL  1W6K: ------------------------------YFKDLPKAHTAFEGALNGMTFYVGLQAEDGHWTGDYGGPL  Targ: FLLPGLVIALHVTGVLNSVLSKHHRVEMCRYLYNHQNEDGGWGLHIEGTSTMFGSALNYVALRLLGEDAD  1W6K: FLLPGLLITCHVARI---PLPAGYREEIVRYLRSVQLPDGGWGLHIEDKSTVFGTALNYVSLRILGVGPD  Targ: GGDGGAMTKARAWILERGGATAITSWGKLWLSVLGVYEWSGNNPLPPEFWLLPYSLPFHPGRMWCHCRMV  1W6K: DPD---LVRARNILHKKGGAVAIPSWGKFWLAVLNVYSWEGLNTLFPEMWLFPDWAPAHPSTLWCHCRQV  Targ: YLPMSYLYGKRFVGPITPKVLSLRQELYTIPYHEIDWNKSRNTCAKEDLYYPHPKMQDILWGSIYHVYEP  1W6K: YLPMSYCYAVRLSAAEDPLVQSLRQELYVEDFASIDWLAQRNNVAPDELYTPHSWLLRVVYALL-----N  Targ: LFTRWPGKRLREKALQAAMKHIHYEDENSRYICLGPVNKVLNMLCCW-VEDPYSDAFKLHLQRVHDYLWV  1W6K: LYEHHHSAHLRQRAVQKLYEHIVADDRFTKSISIGPISKTINMLVRWYVDGPASTAFQEHVSRIPDYLWM  Targ: AEDGMRMQGYNGSQLWDTAFSIQAIVATK--LVDSYAPTLRKAHDFVKDSQIQEDCPGDPNVWFRHIHKG  1W6K: GLDGMKMQGTNGSQIWDTAFAIQALLEAGGHHRPEFSSCLQKAHEFLRLSQVPDNPP-DYQKYYRQMRKG  Targ: AWPLSTRDHGWLISDCTAEGLKASLMLSKLPSTMVGEPLEKNRLCDAVNVLLSLQNDNGGFASYELTRSY  1W6K: GFSFSTLDCGWIVSDCTAEALKAVLLLQEKCP-HVTEHIPRERLCDAVAVLLNMRNPDGGFATYETKRGG  Targ: PWLELINPAETFGDIVIDYPYVECTAATMEALTLFKKLHPGHRTKEIDTAIGKAANFLEKMQRADGSWYG  1W6K: HLLELLNPSEVFGDIMIDYTYVECTSAVMQALKYFHKRFPEHRAAEIRETLTQGLEFCRRQQRADGSWEG  Targ: CWGVCFTYAGWFGIKGLVAAGRTYNS---CLAIRKACEFLLSKELPGGGWGESYLSCQNKVYTNLEGNKP  1W6K: SWGVCFTYGTWFGLEAFACMGQTYRDGTACAEVSRACDFLLSRQMADGGWGEDFESCEERRYVQS--AQS  Targ: HLVNTAWVLMALIEAGQGERDPAPLHRAARLLMNSQLENGDFVQQEIMGVFNKNCMITYAAYRNIFPIWA  1W6K: QIHNTCWAMMGLMAV--RHPDIEAQERGVRCLLEKQLPNGDWPQENIAGVFNKSCAISYTSYRNIFPIWA  Targ: LGEYCHRVLTE------  1W6K: LGRFSQLYPERALAGHP |
| 108864084 | Targ: ----------------------------------------------------------------------  1W6K: -------------TEGTCLRRRGGPYKTEPATDLGRWRLNCERGRQTWTYLQDERAGREQTGLEAYALGL  Targ: --------------------------EDVMGEAVWSSLKRAISRVCNLQAHDGHWPGDYAGLMFFLPGLI  1W6K: -------------------DTKNYFKDLPKAHTAFEGALNGMTFYVGLQAEDGHWTGDYGGPLFLLPGLL  Targ: ITLHVSGVLNTVLSSEHQKEMRRYIYNHQNEDGGWGLHIEGHSTMLGSSLNYVALRLLGEGPNGGDGCIE  1W6K: ITCHVARI---PLPAGYREEIVRYLRSVQLPDGGWGLHIEDKSTVFGTALNYVSLRILGVGPDDPD--LV  Targ: NGRNWILDHGGATFTTSWGKFWLSVLGVFDWSGNNPVPPELLLLPYQLPFHPGRMSSYIRMVFIPMSYIY  1W6K: RARNILHKKGGAVAIPSWGKFWLAVLNVYSWEGLNTLFPEMWLFPDWAPAHPSTLWCHCRQVYLPMSYCY  Targ: GKRFVGPVTPVVLELRSELYNDPYDEIDWNKARTQCAKEDMYYPRSSKLDMFWSFLHKFIEPVLLRWPGR  1W6K: AVRLSAAEDPLVQSLRQELYVEDFASIDWLAQRNNVAPDELYTPHSWLLRVVYALLN-----LYEHHHSA  Targ: KLREKALATSMRNVHYEDECTRYICFGGVPKALNILACW-IEDPSSEAFKCHIARVYDYLWIAEDGMKMQ  1W6K: HLRQRAVQKLYEHIVADDRFTKSISIGPISKTINMLVRWYVDGPASTAFQEHVSRIPDYLWMGLDGMKMQ  Targ: IYDGSQVWDAGLTVEALVATD--LVKELGPTLKRAHSFLKNSQLLDNCPRDFNRWYRHISKGGWTFTTAD  1W6K: GTNGSQIWDTAFAIQALLEAGGHHRPEFSSCLQKAHEFLRLSQVPDNPP-DYQKYYRQMRKGGFSFSTLD  Targ: DGWQVSDCTATALKACLLLSRISPEIVGEPLEIDAQYDAVNCLMSLMNDNGGFSAFELVRSNTWLEHINP  1W6K: CGWIVSDCTAEALKAVLLLQEKCPHVT-EHIPRERLCDAVAVLLNMRNPDGGFATYETKRGGHLLELLNP  Targ: TEAFGRVMIEYPYVECTSSSIQCLALFKKLHPGHRKEEVENCISKGANFIESSQRSDGSWYGSWGICFTY  1W6K: SEVFGDIMIDYTYVECTSAVMQALKYFHKRFPEHRAAEIRETLTQGLEFCRRQQRADGSWEGSWGVCFTY  Targ: ATWFAVTGLVSAGRTLGN---SATVRKACDFLLSKQLPSGGWGESYLSCHDEVYTNLKGNRPHGTHTAWA  1W6K: GTWFGLEAFACMGQTYRDGTACAEVSRACDFLLSRQMADGGWGEDFESCEERRYVQSAQSQIH--NTCWA  Targ: MIALIDAGQAERDPVPLHRAAKALLNLQLEDGEFPQQEIVGVFLQTAMISYSQYRNIFPIMALTGYRRRV  1W6K: MMGLMAVRHP--DIEAQERGVRCLLEKQLPNGDWPQENIAGVFNKSCAISYTSYRNIFPIWALGRFSQLY  Targ: LLAGNI---  1W6K: PERALAGHP |
| 30699377 | Targ: ----------------------------------------------------------------------  1W6K: ------TEGTCLRRRGGPYKTEPATDLGRWRLNCERGRQTWTYLQDERAGREQTGLEAYALGLDTKNYFK  Targ: ----------------------------EGITYKNATDALRRAVSFYSALQSSDGHWPAEITGTLFFLPP  1W6K: ----------------------------DLPKAHTAFEGALNGMTFYVGLQAEDGHWTGDYGGPLFLLPG  Targ: LVFCFYITGHLEKI-FDAEHRKEMLRHIYCHQNEDGGWGLHIEGKSVMFCTVLNYICLRMLGEGPNGGRN  1W6K: LL----ITCHVARIPLPAGYREEIVRYLRSVQLPDGGWGLHIEDKSTVFGTALNYVSLRILGVGPD---D  Targ: NACKRARQWILDHGGVTYIPSWGKIWLSILGIYDWSGTNPMPPEIWLLPSFFPIHLGKTLCYTRMVYMPM  1W6K: PDLVRARNILHKKGGAVAIPSWGKFWLAVLNVYSWEGLNTLFPEMWLFPDWAPAHPSTLWCHCRQVYLPM  Targ: SYLYGKRFVGPLTPLIMLLRKELHLQPYEEINWNKARRLCAKEDMIYPHPLVQDLLWDTLHNFVEPILTN  1W6K: SYCYAVRLSAAEDPLVQSLRQELYVEDFASIDWLAQRNNVAPDELYTPHSWLLRVVYALLNLYEHHHSAH  Targ: WPLKKLVREKALRVAMEHIHYEDENSHYITIGCVEKVLCMLACW-IENPNGDHFKKHLARIPDFMWVAED  1W6K: ------LRQRAVQKLYEHIVADDRFTKSISIGPISKTINMLVRWYVDGPASTAFQEHVSRIPDYLWMGLD  Targ: GLKMQ-SFGSQLWDTVFAIQALLACDLS--DETDDVLRKGHSFIKKSQVRENPSGDFKSMYRHISKGAWT  1W6K: GMKMQGTNGSQIWDTAFAIQALLEAGGHHRPEFSSCLQKAHEFLRLSQVPDNPP-DYQKYYRQMRKGGFS  Targ: LSDRDHGWQVSDCTAEALKCCMLLSMMPAEVVGQKIDPEQLYDSVNLLLSLQGEKGGLTAWEPVRAQEWL  1W6K: FSTLDCGWIVSDCTAEALKAVLLLQEKCPH-VTEHIPRERLCDAVAVLLNMRNPDGGFATYETKRGGHLL  Targ: ELLNPTDFFTCVMAEREYVECTSAVIQALVLFKQLYPDHRTKEIIKSIEKGVQFIESKQTPDGSWHGNWG  1W6K: ELLNPSEVFGDIMIDYTYVECTSAVMQALKYFHKRFPEHRAAEIRETLTQGLEFCRRQQRADGSWEGSWG  Targ: ICFIYATWFALSGLAAAGKTYKS---CLAVRKGVDFLLAIQEEDGGWGESHLSCPEQRYIPLEGNRSNLV  1W6K: VCFTYGTWFGLEAFACMGQTYRDGTACAEVSRACDFLLSRQMADGGWGEDFESCEERRYV--QSAQSQIH  Targ: QTAWAMMGLIHAGQAERDPTPLHRAAKLIITSQLENGDFPQQEILGVFMNTCMLHYATYRNIFPLWALAE  1W6K: NTCWAMMGLMAVRHP--DIEAQERGVRCLLEKQLPNGDWPQENIAGVFNKSCAISYTSYRNIFPIWALGR  Targ: YRKAA----FATHQDL  1W6K: FSQLYPERALAGHP-- |
| 211926830 | Targ: ----------------------------------------------------------------------  1W6K: ------TEGTCLRRRGGPYKTEPATDLGRWRLNCERGRQTWTYLQDERAGREQTGLEAYALGLDTKNYFK  Targ: ----------------------------EKVTFETATSALRRGIHFFSALQASDGHWPAENAGPLFFLPP  1W6K: ----------------------------DLPKAHTAFEGALNGMTFYVGLQAEDGHWTGDYGGPLFLLPG  Targ: LVFCLYITGHL-DEVFTSEHRKEILRYIYCHQKEDGGWGLHIEGHSTMFCTTLNYICMRILGESPDGGHD  1W6K: LL----ITCHVARIPLPAGYREEIVRYLRSVQLPDGGWGLHIEDKSTVFGTALNYVSLRILGVGPD---D  Targ: NACGRAREWILSHGGVTYIPSWGKTWLSILGVFDWSGSNPMPPEFWILPSFFPVHPAKMWSYCRMVYLPM  1W6K: PDLVRARNILHKKGGAVAIPSWGKFWLAVLNVYSWEGLNTLFPEMWLFPDWAPAHPSTLWCHCRQVYLPM  Targ: SYLYGKRFVGPITSLILQLRKELYLQPYEEINWMKVRHLCAKEDTYYPRPLVQELVWDSLYIFAELFLAR  1W6K: SYCYAVRLSAAEDPLVQSLRQELYVEDFASIDWLAQRNNVAPDELYTPHSWLLRVVYALLNLYEHHHSAH  Targ: WPFNKLLREKALQLAMKHIHYEDENSRYITIGCVEKVLCMLACW-VEDPNGDYFKKHLSRISDYLWMAED  1W6K: ------LRQRAVQKLYEHIVADDRFTKSISIGPISKTINMLVRWYVDGPASTAFQEHVSRIPDYLWMGLD  Targ: GMKMQSF-GSQLWDTGFAMQALLASN--LSSEISDVLRRGHEFIKNSQVGENPSGDYKSMYRHISKGAWT  1W6K: GMKMQGTNGSQIWDTAFAIQALLEAGGHHRPEFSSCLQKAHEFLRLSQVPDNPP-DYQKYYRQMRKGGFS  Targ: FSDRDHGWQVSDCTAHGLKCCLLFSMLAPDIVGPKQDPERLHDSVNILLSLQSKNGGMTAWEPAGAPKWS  1W6K: FSTLDCGWIVSDCTAEALKAVLLLQEKCPH-VTEHIPRERLCDAVAVLLNMRNPDGGFATYETKRGGHLL  Targ: ELLNPTEMFSDIVIEHEYSECTSSAIQALSLFKQLYPDHRTTEITAFIKKAAEYLENMQTRDGSWYGNWG  1W6K: ELLNPSEVFGDIMIDYTYVECTSAVMQALKYFHKRFPEHRAAEIRETLTQGLEFCRRQQRADGSWEGSWG  Targ: ICFTYGTWFALAGLAAAGKTFND---CEAIRKGVQFLLAAQKDNGGWGESYLSCSKKIYIAQVGEISNVV  1W6K: VCFTYGTWFGLEAFACMGQTYRDGTACAEVSRACDFLLSRQMADGGWGEDFESCEERRYVQS--AQSQIH  Targ: QTAWALMGLIHSGQAERDPIPLHRAAKLIINSQLESGDFPQQQATGVFLKNCTLHYAAYRNIHPLWALAE  1W6K: NTCWAMMGLMAVRHPDIE--AQERGVRCLLEKQLPNGDWPQENIAGVFNKSCAISYTSYRNIFPIWALGR  Targ: YRARVSL------P  1W6K: FSQLYPERALAGHP |
| 300591977 | Targ: ----------------------------------------------------------------------  1W6K: ------TEGTCLRRRGGPYKTEPATDLGRWRLNCERGRQTWTYLQDERAGREQTGLEAYALGLDTKNYFK  Targ: ---------------------------EEITYEKSTAALRRAVHFYSALQASDGHWPAENAGPLFFLPPL  1W6K: ---------------------------DLPKAHTAFEGALNGMTFYVGLQAEDGHWTGDYGGPLFLLPGL  Targ: VMCMYITGHL-NTVFPAEHQKEILRYIYYHQNEDGGWGLHIEGHSTMFCTALSYICMRILGEGPDGGQDN  1W6K: L----ITCHVARIPLPAGYREEIVRYLRSVQLPDGGWGLHIEDKSTVFGTALNYVSLRILGVGPD---DP  Targ: ACARARKWILDHGGVTHMPSWGKTWLSILGIFEWIGSNPMPPEFWILPSFLPMHPAKMWCYCRMVYMPMS  1W6K: DLVRARNILHKKGGAVAIPSWGKFWLAVLNVYSWEGLNTLFPEMWLFPDWAPAHPSTLWCHCRQVYLPMS  Targ: YLYGKRFVGPITPLILQLREELYTQPYHQVNWKKVRHLCAKEDIYYPHPLIQDLLWDSLYIFTEPLLTRW  1W6K: YCYAVRLSAAEDPLVQSLRQELYVEDFASIDWLAQRNNVAPDELYTPHSWLLRVVYALLNLYEHHH----  Targ: PFNKLVREKALQVTMKHIHYEDENSRYITIGCVEKVLCMLACW-VEDPNGDYFKKHIARIPDYIWVAEDG  1W6K: --SAHLRQRAVQKLYEHIVADDRFTKSISIGPISKTINMLVRWYVDGPASTAFQEHVSRIPDYLWMGLDG  Targ: IKMQSF-GSQEWDTGFAIQALLASN--LTDEIGPTLARGHDFIKKSQVKDNPSGDFESMHRHISKGSWTF  1W6K: MKMQGTNGSQIWDTAFAIQALLEAGGHHRPEFSSCLQKAHEFLRLSQVPDNPP-DYQKYYRQMRKGGFSF  Targ: SDQDHGWQVSDCTAEGLKCCLLFSIMPPEIVGEKMEPEQLYDSVNVLLSLQSKNGGLAAWEPAGAQEWLE  1W6K: STLDCGWIVSDCTAEALKAVLLLQEKCPH-VTEHIPRERLCDAVAVLLNMRNPDGGFATYETKRGGHLLE  Targ: LLNSTEFFADIVIEHEYIECTASAMQTLVLFKKLYPGHRKKEIENFIKNAAQFLQVIQMPDGSWYGNWGV  1W6K: LLNPSEVFGDIMIDYTYVECTSAVMQALKYFHKRFPEHRAAEIRETLTQGLEFCRRQQRADGSWEGSWGV  Targ: CFTYGTWFALGGLAAVGKTYNN---CLAVRRAVDFLLRAQRDNGGWGESYLSCPKKEYVPLEGNKSNLVH  1W6K: CFTYGTWFGLEAFACMGQTYRDGTACAEVSRACDFLLSRQMADGGWGEDFESCEERRYV--QSAQSQIHN  Targ: TAWAMMGLIHAGQAERDPTPLHRAAKLIINSQLEDGDFPQQEITGVFMKNCMLHYAAYKNIYPLWALAEY  1W6K: TCWAMMGLMAVRHP--DIEAQERGVRCLLEKQLPNGDWPQENIAGVFNKSCAISYTSYRNIFPIWALGRF  Targ: RKHVPLPLGKNLNQVVNCIGQSLYKKYK  1W6K: SQLYPERALAGHP--------------- |
| 300592003 | Targ: ----------------------------------------------------------------------  1W6K: ------TEGTCLRRRGGPYKTEPATDLGRWRLNCERGRQTWTYLQDERAGREQTGLEAYALGLDTKNYFK  Targ: ---------------------------EEITYEKATTAVRRAAHHLSALQTSDGHWPAQIAGPLFFLPPL  1W6K: ---------------------------DLPKAHTAFEGALNGMTFYVGLQAEDGHWTGDYGGPLFLLPGL  Targ: VFCMYITGHLDSVFPEEYRKEILRYIYYHQNEDGGWGLHIEGHSTMFCTALNYICMRILGEGPDGGQDNA  1W6K: LITCHVAR---IPLPAGYREEIVRYLRSVQLPDGGWGLHIEDKSTVFGTALNYVSLRILGVGPD---DPD  Targ: CARARKWIHDHGGVTHIPSWGKTWLSILGVFDWCGSNPMPPEFWILPSFLPMHPAKMWCYCRLVYMPMSY  1W6K: LVRARNILHKKGGAVAIPSWGKFWLAVLNVYSWEGLNTLFPEMWLFPDWAPAHPSTLWCHCRQVYLPMSY  Targ: LYGKRFVGPITPLILQLREELFTEPYEKVNWKKARHQCAKEDLYYPHPLLQDLIWDSLYLFTEPLLTRWP  1W6K: CYAVRLSAAEDPLVQSLRQELYVEDFASIDWLAQRNNVAPDELYTPHSWLLRVVYALLNLYEHHH-----  Targ: FNKLVREKALQVTMKHIHYEDETSRYITIGCVEKVLCMLACW-VEDPNGDAFKKHLARVPDYLWVSEDGM  1W6K: -SAHLRQRAVQKLYEHIVADDRFTKSISIGPISKTINMLVRWYVDGPASTAFQEHVSRIPDYLWMGLDGM  Targ: TMQSF-GSQEWDAGFAVQALLATNLV--EEIAPTLAKGHDFIKKSQVRDNPSGDFKSMYRHISKGSWTFS  1W6K: KMQGTNGSQIWDTAFAIQALLEAGGHHRPEFSSCLQKAHEFLRLSQVPDNPP-DYQKYYRQMRKGGFSFS  Targ: DQDHGWQVSDCTAEGLKCCLLLSMLPPEIVGEKMEPERLYDSVNVLLSLQSKKGGLSAWEPAGAQEWLEL  1W6K: TLDCGWIVSDCTAEALKAVLLLQEKCP-HVTEHIPRERLCDAVAVLLNMRNPDGGFATYETKRGGHLLEL  Targ: LNPTEFFADIVVEHEYVECTGSAIQALVLFKKLYPGHRKKEIENFIANAVRFLEDTQTADGSWYGNWGVC  1W6K: LNPSEVFGDIMIDYTYVECTSAVMQALKYFHKRFPEHRAAEIRETLTQGLEFCRRQQRADGSWEGSWGVC  Targ: FTYGSWFALGGLAAAGKTFAN---CAAIRKAVKFLLTTQREDGGWGESYLSSPKKIYVPLEGSRSNVVHT  1W6K: FTYGTWFGLEAFACMGQTYRDGTACAEVSRACDFLLSRQMADGGWGEDFESCEERRYVQ--SAQSQIHNT  Targ: AWALMGLIHAGQAERDPAPLHRAAKLIINSQLEEGDWPQQEITGVFMKNCMLHYPMYRDIYPMWALAEYR  1W6K: CWAMMGLMAVRHPDIE--AQERGVRCLLEKQLPNGDWPQENIAGVFNKSCAISYTSYRNIFPIWALGRFS  Targ: RRVPLPSTPVCLT--  1W6K: QLYPERA---LAGHP |
| 353678016 | Targ: ----------------------------------------------------------------------  1W6K: ------TEGTCLRRRGGPYKTEPATDLGRWRLNCERGRQTWTYLQDERAGREQTGLEAYALGLDTKNYFK  Targ: ---------------------------EGITYEKATRALRRTVQFFSALQASDGHWPAEIAGPLFFLPPL  1W6K: ---------------------------DLPKAHTAFEGALNGMTFYVGLQAEDGHWTGDYGGPLFLLPGL  Targ: VMCVYITGHL-DAVFPAEHRKEILRYIYYHQNEDGGWGLHIEGHSTMFCTALNYICMRIIGEGPNGGQDD  1W6K: L----ITCHVARIPLPAGYREEIVRYLRSVQLPDGGWGLHIEDKSTVFGTALNYVSLRILGVGPD---DP  Targ: ACARARKWIHDHGSVTNIPSWGKTWLSILGVYDWSGSNPMPPEFWMLPSFLPMHPAKMWCYCRMVYMPMS  1W6K: DLVRARNILHKKGGAVAIPSWGKFWLAVLNVYSWEGLNTLFPEMWLFPDWAPAHPSTLWCHCRQVYLPMS  Targ: YLYGKRFVGPITPLIQQLREELFTQPYDQINWKKTRHQCAPEDLYYPHPFVQDLIWDCLYIFTEPLLTRW  1W6K: YCYAVRLSAAEDPLVQSLRQELYVEDFASIDWLAQRNNVAPDELYTPHSWLLRVVYALLNLYEHHHSAH-  Targ: PLNEIIRKKALEVTMKHIHYEDESSRYITIGCVEKVLCMLACW-VEDPNGDYFKKHLARIPDYIWVAEDG  1W6K: -----LRQRAVQKLYEHIVADDRFTKSISIGPISKTINMLVRWYVDGPASTAFQEHVSRIPDYLWMGLDG  Targ: MKMQSF-GSQEWDTGFAIQALLATNLT--DEIGDVLRRGHDFIKKSQVRDNPSGDFKSMYRHISKGSWTF  1W6K: MKMQGTNGSQIWDTAFAIQALLEAGGHHRPEFSSCLQKAHEFLRLSQVPDNPP-DYQKYYRQMRKGGFSF  Targ: SDQDHGWQVSDCTAEGLKCCLLFSMMPPEIVGEHMVPERLYDSVNVLLSLQSKNGGLSAWEPAGAQEWLE  1W6K: STLDCGWIVSDCTAEALKAVLLLQEKCPH-VTEHIPRERLCDAVAVLLNMRNPDGGFATYETKRGGHLLE  Targ: LLNPTEFFADIVIEHEYVECTSSAIHALVLFKKLYPGHRKKEIDNFIVNAVRYLESIQTSDGGWYGNWGV  1W6K: LLNPSEVFGDIMIDYTYVECTSAVMQALKYFHKRFPEHRAAEIRETLTQGLEFCRRQQRADGSWEGSWGV  Targ: CFTYGTWFALGGLAAAGKTYNN---CLAMRKAVDFLLRIQRDNGGWGESYLSCPEKRYVPLEGNRSNLVH  1W6K: CFTYGTWFGLEAFACMGQTYRDGTACAEVSRACDFLLSRQMADGGWGEDFESCEERRYVQ--SAQSQIHN  Targ: TAWALMALIHAGQMDRDPTPLHRAARLMINSQLEDGDFPQQEITGVFMKNCMLHYAAYRNIYPLWALAEY  1W6K: TCWAMMGLMAVRHP--DIEAQERGVRCLLEKQLPNGDWPQENIAGVFNKSCAISYTSYRNIFPIWALGRF  Targ: RRRVPLPS-----  1W6K: SQLYPERALAGHP |
| 118901781 | Targ: ----------------------------------------------------------------------  1W6K: -------TEGTCLRRRGGPYKTEPATDLGRWRLNCERGRQTWTYLQDERAGREQTGLEAYALGLDTKNYF  Targ: --------------------------GEEITYEAATTTLRRAVHYFSALQADDGHWPAENAGPLFFLPPL  1W6K: --------------------------KDLPKAHTAFEGALNGMTFYVGLQAEDGHWTGDYGGPLFLLPGL  Targ: VMCLYITGHLNTVFPAEHRIEILRYIYCHQNDDGGWGLHIEGHSTMFCTALSYICMRILGEGRDGGENNA  1W6K: LITCHVAR---IPLPAGYREEIVRYLRSVQLPDGGWGLHIEDKSTVFGTALNYVSLRILGVGPDDPD---  Targ: CARARKWILDHGSVTAIPSWGKTWLSILGLFDWSGSNPMPPEFWILPPFLPMHPAKMWCYCRMVYMPMSY  1W6K: LVRARNILHKKGGAVAIPSWGKFWLAVLNVYSWEGLNTLFPEMWLFPDWAPAHPSTLWCHCRQVYLPMSY  Targ: LYGKRFVGPITPLILQLREELYAQAYDEINWRKVRHNCAKEDLYYPHPLIQDLMWDSLYIFTEPFLTRWP  1W6K: CYAVRLSAAEDPLVQSLRQELYVEDFASIDWLAQRNNVAPDELYTPHSWLLRVVYALLNLYE-----HHH  Targ: FNKLREKALQTTMKHIHYEDENSRYITIGCVEKVLCMLACW-VEDPNGDYFKQHLARIPDYIWVAEDGMK  1W6K: SAHLRQRAVQKLYEHIVADDRFTKSISIGPISKTINMLVRWYVDGPASTAFQEHVSRIPDYLWMGLDGMK  Targ: MQSF-GSQEWDTGFAIQALLASD--LIDEIRPTLMKGHDFIKKSQVKENPSGDFKSMHRHISKGSWTFSD  1W6K: MQGTNGSQIWDTAFAIQALLEAGGHHRPEFSSCLQKAHEFLRLSQVPDNPP-DYQKYYRQMRKGGFSFST  Targ: QDHGWQVSDCTAEALKCCLLFSRMPTEIVGDKMEDNQLFDAVNMLLSLQSKNGGLAAWEPAGSSEWLELL  1W6K: LDCGWIVSDCTAEALKAVLLLQEKCPH-VTEHIPRERLCDAVAVLLNMRNPDGGFATYETKRGGHLLELL  Targ: NPTEFFEDIVIEHEYVECTSSAIQAMVMFKKLYPGHRKKEIEVSITNAVQYLEDIQMPDGSWYGNWGVCF  1W6K: NPSEVFGDIMIDYTYVECTSAVMQALKYFHKRFPEHRAAEIRETLTQGLEFCRRQQRADGSWEGSWGVCF  Targ: TYGTWFAMGGLTAAGKTYNN---CQTLHKAVDFLIKSQRSDGGWGESYLSCPNKEYTPLEGNRSNLVHTS  1W6K: TYGTWFGLEAFACMGQTYRDGTACAEVSRACDFLLSRQMADGGWGEDFESCEERRYV--QSAQSQIHNTC  Targ: WAMMGLIHSRQAERDPTPLHRAAKLLINSQMESGDFPQQEITGVFMKNCMLHYAASRNIYPLWALAEYRK  1W6K: WAMMGLMAVRHP--DIEAQERGVRCLLEKQLPNGDWPQENIAGVFNKSCAISYTSYRNIFPIWALGRFSQ  Targ: NVRLPSKSV--  1W6K: LYPERALAGHP |
| 350538403 | Targ: ----------------------------------------------------------------------  1W6K: -----TEGTCLRRRGGPYKTEPATDLGRWRLNCERGRQTWTYLQDERAGREQTGLEAYALGLDTKNYFKD  Targ: ----------------------------EISHEIATVALHRAVHFFSALQATDGHWPAESAGPLFFLPPL  1W6K: ----------------------------LPKAHTAFEGALNGMTFYVGLQAEDGHWTGDYGGPLFLLPGL  Targ: VMCMYITGHLNTVFPAEHRKEILRYIYCHQNEDGGWGLHIEGHSTMFCTAMSYICMRILGEGPEGGVNNA  1W6K: LITCHVAR---IPLPAGYREEIVRYLRSVQLPDGGWGLHIEDKSTVFGTALNYVSLRILGVGPD---DPD  Targ: CARARKWILDHGSVIAIPSWGKTWLSILGAFEWIGTNPMPPEFWILPSFLPVHPAKMWCYCRTVYMPMSY  1W6K: LVRARNILHKKGGAVAIPSWGKFWLAVLNVYSWEGLNTLFPEMWLFPDWAPAHPSTLWCHCRQVYLPMSY  Targ: LYGKRFVGPITPLILKLREELYDQTYDEINWKKVRHVCAKEDLYYPHPFVQDLMWDSLYICTEPLLTRWP  1W6K: CYAVRLSAAEDPLVQSLRQELYVEDFASIDWLAQRNNVAPDELYTPHSWLLRVVYALLN-----LYEHHH  Targ: FNKLRNKALEVTMKHIHYEDENSRYITMGCVEKVLSMLACW-VEDPNGDHFKKHLARIPDFLWVAEDGMK  1W6K: SAHLRQRAVQKLYEHIVADDRFTKSISIGPISKTINMLVRWYVDGPASTAFQEHVSRIPDYLWMGLDGMK  Targ: MQGC-GSQSWDASLAIQALLASE--MNDEISDTLKNGHDFIKQSQVKDNPSGDFKVMYRHISKGSWAFAD  1W6K: MQGTNGSQIWDTAFAIQALLEAGGHHRPEFSSCLQKAHEFLRLSQVPDNPP-DYQKYYRQMRKGGFSFST  Targ: QDLGWQVSDCTAEALKCCLLFSTMPPEIVGEAMDPVRLYDSVNVILSLQSKNGGLSAWEPAGAPEYLELL  1W6K: LDCGWIVSDCTAEALKAVLLLQEKCPH-VTEHIPRERLCDAVAVLLNMRNPDGGFATYETKRGGHLLELL  Targ: NPTEFFEDIVIEHEHVECTSSAIQALVRFKKLYPGHRTTEVDNFINNGVKYIEDVQEPDGSWYGNWGVCF  1W6K: NPSEVFGDIMIDYTYVECTSAVMQALKYFHKRFPEHRAAEIRETLTQGLEFCRRQQRADGSWEGSWGVCF  Targ: IYASWFALGGLAAVGLSYSN---CAAVRKSVEFLLRTQRSDGGWGESYRSCPDKVYRELETEHSNLVQTA  1W6K: TYGTWFGLEAFACMGQTYRDGTACAEVSRACDFLLSRQMADGGWGEDFESCEERRYVQ--SAQSQIHNTC  Targ: WALMGLIHSGQVERDPRPLHRAAKLLINFQMEDGDFPQQEITGVFLRNCMMHYALYRNIFPLWGLAEYRR  1W6K: WAMMGLMAVR--HPDIEAQERGVRCLLEKQLPNGDWPQENIAGVFNKSCAISYTSYRNIFPIWALGRFSQ  Targ: NVLVPLKHNYI  1W6K: LYPERALAGHP |
| 300807980 | Targ: ----------------------------------------------------------------------  1W6K: -------TEGTCLRRRGGPYKTEPATDLGRWRLNCERGRQTWTYLQDERAGREQTGLEAYALGLDTKNYF  Targ: --------------------------GEEVSYEAVTAALRRGVHLYSALQASDGHWPAENAGPMFFMPPM  1W6K: --------------------------KDLPKAHTAFEGALNGMTFYVGLQAEDGHWTGDYGGPLFLLPGL  Targ: VMCLYITGHLNAIFTEEHRSETLRYIYYHQNEDGGWGFHIEGHSTMFGTVLNYICMRLLGEGPEGGQDNA  1W6K: LITCHVAR---IPLPAGYREEIVRYLRSVQLPDGGWGLHIEDKSTVFGTALNYVSLRILGVGPD---DPD  Targ: VSRGRKWILDHGGATSIPSWGKTWLSIMGLCDWSGCNPMPPEFWLLPSYLPMHPGKMWCYCRMVYMPMSY  1W6K: LVRARNILHKKGGAVAIPSWGKFWLAVLNVYSWEGLNTLFPEMWLFPDWAPAHPSTLWCHCRQVYLPMSY  Targ: LYGKRFTARITPLILQLREEIHIQPYDQIDWKKVRHVCCKEDMYYPHPLLQDLLWDTLYLTTEPLLTRWP  1W6K: CYAVRLSAAEDPLVQSLRQELYVEDFASIDWLAQRNNVAPDELYTPHSWLLRVVYALLN------LYEHH  Targ: LNKLIRKRALQTTMKHIHYEDENSRYITIGCVEKVLCMLACWV-EDPNGDYFKKHLARIPDYLWIAEDGM  1W6K: HSAHLRQRAVQKLYEHIVADDRFTKSISIGPISKTINMLVRWYVDGPASTAFQEHVSRIPDYLWMGLDGM  Targ: KMQSF-GSQHWDTAFSIQALLASN--MAEEIGITLAKGHDFIKKSQVKDNPSGDFKGMYRHISKGAWTFS  1W6K: KMQGTNGSQIWDTAFAIQALLEAGGHHRPEFSSCLQKAHEFLRLSQVPDNPP-DYQKYYRQMRKGGFSFS  Targ: DQDHGWQVSDCTAEGLKCCLLFSMMQPEVVGESMAPESLYNSVNVLLSLQSQNGGLPAWEPAGAPEWLEL  1W6K: TLDCGWIVSDCTAEALKAVLLLQEKCPH-VTEHIPRERLCDAVAVLLNMRNPDGGFATYETKRGGHLLEL  Targ: LNPTEFFENIVIEHEYVECTSSAVQALVLFKKLYPLHRRKEVERFITNGAKYLEDIQMPDGSWYGNWGVC  1W6K: LNPSEVFGDIMIDYTYVECTSAVMQALKYFHKRFPEHRAAEIRETLTQGLEFCRRQQRADGSWEGSWGVC  Targ: FTYGAWFALEGLSAAGKTYNN---CAAVRKGVDFLLNIQLEDGGWGESYQSCPDKKYVPLEDNRSNLVQT  1W6K: FTYGTWFGLEAFACMGQTYRDGTACAEVSRACDFLLSRQMADGGWGEDFESCEERRYV--QSAQSQIHNT  Targ: SWALMGLIYAGQADRDPTPLHRAAQLLINSQLEDGDFPQQEITGVFQRNCMLHYAAYRNIFPLWALAEYR  1W6K: CWAMMGLMAVRHPDIE--AQERGVRCLLEKQLPNGDWPQENIAGVFNKSCAISYTSYRNIFPIWALGRFS  Targ: RQIQLHSEATKMV  1W6K: QLYPERALAGHP- |
| 353558692 | Targ: ----------------------------------------------------------------------  1W6K: ------TEGTCLRRRGGPYKTEPATDLGRWRLNCERGRQTWTYLQDERAGREQTGLEAYALGLDTKNYFK  Targ: ---------------------------EEITREIATTALRRSVHLVSALQASDGHWCAENSGPMFFVPPM  1W6K: ---------------------------DLPKAHTAFEGALNGMTFYVGLQAEDGHWTGDYGGPLFLLPGL  Targ: VFSLYITGHL-NAVFSAEHCKEILRYIYCHPNEDGGWGLHIEGHSAMFSTVLNYNWLGKLGEGRDGGKDN  1W6K: L----ITCHVARIPLPAGYREEIVRYLRSVQLPDGGWGLHIEDKSTVFGTALNYVSLRILGVGPD---DP  Targ: ACERARRRILDHGSATAISSWGKTWLAILGVYEWDGCNPMPPEFWAFPTFFPIHPARMLCYCRLTYMAMS  1W6K: DLVRARNILHKKGGAVAIPSWGKFWLAVLNVYSWEGLNTLFPEMWLFPDWAPAHPSTLWCHCRQVYLPMS  Targ: YLYGKKFVGPITPLILQLREEIYNEPYDQINWSRMRHLCAKEDNYYAHTLTQIILWDAIYMLGEPLLKRW  1W6K: YCYAVRLSAAEDPLVQSLRQELYVEDFASIDWLAQRNNVAPDELYTPHSWLLRVVYALL-----NLYEHH  Targ: PFNKLREKALKITMDHIHYEDENSQYITIGSVEKPLLMLACW-HEDPNGDAFKKHLARIPDYVWLGEDGI  1W6K: HSAHLRQRAVQKLYEHIVADDRFTKSISIGPISKTINMLVRWYVDGPASTAFQEHVSRIPDYLWMGLDGM  Targ: KIQSF-GSQVWDTSFVLQALIASNLPS--ETGPTLEKGHNFIKNSQVTQNPSGDFRRMFRHISKGSWTFS  1W6K: KMQGTNGSQIWDTAFAIQALLEAGGHHRPEFSSCLQKAHEFLRLSQVPDNPP-DYQKYYRQMRKGGFSFS  Targ: DKDHGWQVSDCTAESLKCCLLFSMMPPELVGEKMGPQRMYDAVNVIISLQSKNGGCSAWEPAGAGSWMEW  1W6K: TLDCGWIVSDCTAEALKAVLLLQEKCPH-VTEHIPRERLCDAVAVLLNMRNPDGGFATYETKRGGHLLEL  Targ: LNPVEFLADLVIEHEYVECTSSSLQALVLFKKLYPEHRRKEIEIFILNAVRFTEEIQQPDGSWYGNWGIC  1W6K: LNPSEVFGDIMIDYTYVECTSAVMQALKYFHKRFPEHRAAEIRETLTQGLEFCRRQQRADGSWEGSWGVC  Targ: FLSGTWFGLKGLAAAGKTYYN---CTAVRKGVEFLLQTQRDDGGWGESYLSCPKKIYVPLEGNRSNLVQT  1W6K: FTYGTWFGLEAFACMGQTYRDGTACAEVSRACDFLLSRQMADGGWGEDFESCEERRYVQ--SAQSQIHNT  Targ: ALAMMGLILGGQGERDPTPLHRAAKLLINSQTELGDFPQQELSGCFMRNCMLHYSEYRDIFPTWALAEYC  1W6K: CWAMMGLMAVRH--PDIEAQERGVRCLLEKQLPNGDWPQENIAGVFNKSCAISYTSYRNIFPIWALGRFS  Targ: KLFPLPSKND--  1W6K: QLYPERALAGHP |
| 82468803 | Targ: ----------------------------------------------------------------------  1W6K: ------TEGTCLRRRGGPYKTEPATDLGRWRLNCERGRQTWTYLQDERAGREQTGLEAYALGLDTKNYFK  Targ: ---------------------------EEITSEIAAAALRRSVHLFSALQASDGHWCAENGGLLFFLPPL  1W6K: ---------------------------DLPKAHTAFEGALNGMTFYVGLQAEDGHWTGDYGGPLFLLPGL  Targ: VFAVYITGHLNTVFSPEHRKEILRYIYCHQNEDGGWGIHIEGHSTMFCTVLNYICMRILGEARDGGIENA  1W6K: LITCHVA---RIPLPAGYREEIVRYLRSVQLPDGGWGLHIEDKSTVFGTALNYVSLRILGVGPD---DPD  Targ: CERGRKWILDHGGATGISSWGKTWLSILGVYEWDGTNPMPPEFWAFPSSFPLHPAKMFCYCRITYMPMSY  1W6K: LVRARNILHKKGGAVAIPSWGKFWLAVLNVYSWEGLNTLFPEMWLFPDWAPAHPSTLWCHCRQVYLPMSY  Targ: LYGKRFVGPITPLILQIREEIYNEPYNKIKWNSVRHLCAKEDNYFPHPTIQKLLWDALYTFSEPLFSRWP  1W6K: CYAVRLSAAEDPLVQSLRQELYVEDFASIDWLAQRNNVAPDELYTPHSWLLRVVYALLN-----LYEHHH  Targ: FNKLREKALKITMDHIHYEDHNSRYITIGCVEKPLCMLACW-IEDPHGEAFKKHLARIADYIWVGEDGIK  1W6K: SAHLRQRAVQKLYEHIVADDRFTKSISIGPISKTINMLVRWYVDGPASTAFQEHVSRIPDYLWMGLDGMK  Targ: MQSF-GSQTWDTSLALQALIASDLS--HEIGPTLKQGHVFTKNSQATENPSGDFRKMFRHISKGAWTFSD  1W6K: MQGTNGSQIWDTAFAIQALLEAGGHHRPEFSSCLQKAHEFLRLSQVPDNPP-DYQKYYRQMRKGGFSFST  Targ: KDQGWQVSDCTAESLKCCLLFSMMPPEIVGEKMEPEKVYDSVNVILSLQSQNGGFTAWEPARAGSWMEWL  1W6K: LDCGWIVSDCTAEALKAVLLLQEKCPH-VTEHIPRERLCDAVAVLLNMRNPDGGFATYETKRGGHLLELL  Targ: NPVEFMEDLVVEHEYVECTSSAIQALVLFKKLYPRHRNKEIENCIINAAQFIENIQEPDGSWYGNWGICF  1W6K: NPSEVFGDIMIDYTYVECTSAVMQALKYFHKRFPEHRAAEIRETLTQGLEFCRRQQRADGSWEGSWGVCF  Targ: SYGTWFALKGLAAAGRTYEN---CSAIRKGVDFLLKSQRDDGGWAESYLSCPKKVYVPFEGNRSNLVQTA  1W6K: TYGTWFGLEAFACMGQTYRDGTACAEVSRACDFLLSRQMADGGWGEDFESCEERRYV--QSAQSQIHNTC  Targ: WAMMGLIYGGQAKRDPMPLHRAAKLLINSQTDLGDFPQQELTGAFMRNCMLHYALFRNTFPIWALAEYRR  1W6K: WAMMGLMAVRHP--DIEAQERGVRCLLEKQLPNGDWPQENIAGVFNKSCAISYTSYRNIFPIWALGRFSQ  Targ: HVLFPSAGFGFGFTNNL  1W6K: LYPERALAGHP------ |
| 353678133 | Targ: ----------------------------------------------------------------------  1W6K: ------TEGTCLRRRGGPYKTEPATDLGRWRLNCERGRQTWTYLQDERAGREQTGLEAYALGLDTKNYFK  Targ: ---------------------------EEINYETVTNAIRRSAHYLSATQSSDGFWPADASAPVFYLAPW  1W6K: ---------------------------DLPKAHTAFEGALNGMTFYVGLQAEDGHWTGDYGGPLFLLPGL  Targ: VIGLYVIGHLNTVFPAEHQKEILRYIYCHQNEDGGWGLHYEDGGTMFGTAFNYVCMRILGEGPGGGRDNA  1W6K: LITCHVAR---IPLPAGYREEIVRYLRSVQLPDGGWGLHIEDKSTVFGTALNYVSLRILGVGPD---DPD  Targ: CERARKGILDHGGVTYIPSGGKTWLAMLGVFDWSGCNPMPPEFWMLPPFFPMHPAQMWCYCRIVYMPMSY  1W6K: LVRARNILHKKGGAVAIPSWGKFWLAVLNVYSWEGLNTLFPEMWLFPDWAPAHPSTLWCHCRQVYLPMSY  Targ: LYGRRFVGPITPLVQQLREELHTQPFHEIEWSKARHLCAKEDLFHRRPWIQELFWDCLHTFAEPLLTRWP  1W6K: CYAVRLSAAEDPLVQSLRQELYVEDFASIDWLAQRNNVAPDELYTPHSWLLRVVYALLNLYEHHH-----  Targ: LNNFIREKALKITMEHVHYDDKASHYINPGSVEKVICMVACWV-EDPSGEPFQRHLARISDYVWIAEDGM  1W6K: -SAHLRQRAVQKLYEHIVADDRFTKSISIGPISKTINMLVRWYVDGPASTAFQEHVSRIPDYLWMGLDGM  Targ: RITGI-GSQTWDAALSIQALIACN--LIEEMGPTLKKGYDFLKNSQAKDNPPGDFKRMYRHFGKGAWAFS  1W6K: KMQGTNGSQIWDTAFAIQALLEAGGHHRPEFSSCLQKAHEFLRLSQVPDNPP-DYQKYYRQMRKGGFSFS  Targ: SQDYGVIALDCTAESLMCCLHFSMMPPEIVGEKLEPEKLYLAVDFILSLQSKNGGLTCWEPARGGKWLEV  1W6K: TLDCGWIVSDCTAEALKAVLLLQEKCPH-VTEHIPRERLCDAVAVLLNMRNPDGGFATYETKRGGHLLEL  Targ: LNPLEFFENIVVEHEYVEVTASAINALVMFKKRYPGYREKEIEHFISKAVHYLIQTQFPNGPWYGVWGIC  1W6K: LNPSEVFGDIMIDYTYVECTSAVMQALKYFHKRFPEHRAAEIRETLTQGLEFCRRQQRADGSWEGSWGVC  Targ: FMYGTYFALKGLAAAGNTYAN---CPAIPKAVDFLLKTQCQDGGWGESYLSGTTKVYTPLEGNRSNLVQT  1W6K: FTYGTWFGLEAFACMGQTYRDGTACAEVSRACDFLLSRQMADGGWGEDFESCEERRYV--QSAQSQIHNT  Targ: AWALMGLIHSGQAERDPTPLHRSAKLLINSQTSDGDFPQQDSTGLLKGSCAMHYAAYRNIFPLWALAAYR  1W6K: CWAMMGLMAVRHP--DIEAQERGVRCLLEKQLPNGDWPQENIAGVFNKSCAISYTSYRNIFPIWALGRFS  Targ: THVLGLTSKAHSSAVME  1W6K: QLYPERALAGHP----- |
| 300591987 | Targ: ----------------------------------------------------------------------  1W6K: ------TEGTCLRRRGGPYKTEPATDLGRWRLNCERGRQTWTYLQDERAGREQTGLEAYALGLDTKNYFK  Targ: ---------------------------EEISYEMALDAMRRGAHFLAAIQASDGHWPSETSGPLFYVCPL  1W6K: ---------------------------DLPKAHTAFEGALNGMTFYVGLQAEDGHWTGDYGGPLFLLPGL  Targ: LICMYIMGFMDKVFSPEHKKEMMRYIYNHQNEDGGWGLHVGGHSNMFCTTFNYISLRLLGEEPDVEAVCK  1W6K: LITCHVAR---IPLPAGYREEIVRYLRSVQLPDGGWGLHIEDKSTVFGTALNYVSLRILGVGPDDPDLVR  Targ: ARNWIHDHDGVTSILSWGKTWLSILNVFDWSASNPMPPEYWMLPTWVPIHPSNMMCYTRITYMPMSYLYG  1W6K: ARNILHKKGGAVAIPSWGKFWLAVLNVYSWEGLNTLFPEMWLFPDWAPAHPSTLWCHCRQVYLPMSYCYA  Targ: KRFQAPLTPLVLQLRDELHTQPYDQINWRKVRHMCATEDLYFPHPFVQDLLWDTLYLLSEPLMTRWPFNK  1W6K: VRLSAAEDPLVQSLRQELYVEDFASIDWLAQRNNVAPDELYTPHSWLLRVVYALLNLYE------HHHSA  Targ: LIRQKALNETMRHIHYEDENSRYITIGCVEKPLCMLACW-VEDPNSEYVKKHLARIPDYLWMAEDGMKMQ  1W6K: HLRQRAVQKLYEHIVADDRFTKSISIGPISKTINMLVRWYVDGPASTAFQEHVSRIPDYLWMGLDGMKMQ  Targ: SF-GSQSWDAALAMQALLSCNITR--EIGSVLNSGHDFIKNSQVRNNPPGDYKSMFRYMSKGSWTFSDCD  1W6K: GTNGSQIWDTAFAIQALLEAGGHHRPEFSSCLQKAHEFLRLSQVPDNPP-DYQKYYRQMRKGGFSFSTLD  Targ: HGWQVSDCTAENLKCCLLLSLLPPDIVGEKMEPERFYDAVNVILNMQSKNGGLPAWEPASSYYWMEWLNP  1W6K: CGWIVSDCTAEALKAVLLLQEKCPH-VTEHIPRERLCDAVAVLLNMRNPDGGFATYETKRGGHLLELLNP  Targ: VEFLEDLIIEHQHVECTSSALQAILLFRKQYPGHRRKEINNFINKAVQFLQDIQLPDGSWYGNWGICYTY  1W6K: SEVFGDIMIDYTYVECTSAVMQALKYFHKRFPEHRAAEIRETLTQGLEFCRRQQRADGSWEGSWGVCFTY  Targ: GTWFALKALSMAGKTYEN---CEAVRKGANFLRKIQNPEGGFGESYLSCPYKRYIPLDGKRSNLVQTAWG  1W6K: GTWFGLEAFACMGQTYRDGTACAEVSRACDFLLSRQMADGGWGEDFESCEERRYV--QSAQSQIHNTCWA  Targ: MMGLICAGQADVDPTPIHRAAKLLINSQTEDGDFPQEEITGEFFKNCTLHFAAFREVFPVMALGEYCNKV  1W6K: MMGLMAVRHPDIE--AQERGVRCLLEKQLPNGDWPQENIAGVFNKSCAISYTSYRNIFPIWALGRFSQLY  Targ: PLPSKKK--  1W6K: PERALAGHP |
| 240256372 | Targ: ----------------------------------------------------------------------  1W6K: ------TEGTCLRRRGGPYKTEPATDLGRWRLNCERGRQTWTYLQDERAGREQTGLEAYALGLDTKNYFK  Targ: ----------------------------KKITFEDAKNTLRRGIHYMAALQSDDGHWPSENAGCIFFNAP  1W6K: ----------------------------DLPKAHTAFEGALNGMTFYVGLQAEDGHWTGDYGGPLFLLPG  Targ: FVICLYITGHLDKVFSEEHRKEMLRYMYNHQNDDGGWGIDVESHSFMFCTVINYICLRIFGVDPDHDGES  1W6K: LLITCHVARI---PLPAGYREEIVRYLRSVQLPDGGWGLHIEDKSTVFGTALNYVSLRILGVGPD---DP  Targ: ACARARKWIIDHGGATYTPLFGKAWLSVLGVYEWSGCKPIPPEFWFFPSYFPINGGTLWIYLRDTFMAMS  1W6K: DLVRARNILHKKGGAVAIPSWGKFWLAVLNVYSWEGLNTLFPEMWLFPDWAPAHPSTLWCHCRQVYLPMS  Targ: YLYGKKFVAKPTPLILQLREELYPQPYAEIVWSQARSRCAKEDLYYPQSLVQDLFWKLVHMFSENILNRW  1W6K: YCYAVRLSAAEDPLVQSLRQELYVEDFASIDWLAQRNNVAPDELYTPHSWLLRVVYALLNLYEHH-----  Targ: PFNKLIREKAIRTAMELIHYHDEATRYITGGAVPKVFHMLACW-VEDPESDYFKKHLARVSHFIWIAEDG  1W6K: -HSAHLRQRAVQKLYEHIVADDRFTKSISIGPISKTINMLVRWYVDGPASTAFQEHVSRIPDYLWMGLDG  Targ: LKIQ-TFGSQIWDTAFVLQVMLAADVDD--EIRPTLIKGYSYLRKSQFTENPPGDYINMFRDISKGGWGY  1W6K: MKMQGTNGSQIWDTAFAIQALLEAGGHHRPEFSSCLQKAHEFLRLSQVPDNPP-DYQKYYRQMRKGGFSF  Targ: SDKDQGWPVSDCISESLECCLIFESMSSEFIGEKMEVERLYDAVNMLLYMQSRNGGISIWEAASGKKWLE  1W6K: STLDCGWIVSDCTAEALKAVLLLQEKCPH-VTEHIPRERLCDAVAVLLNMRNPDGGFATYETKRGGHLLE  Targ: WLSPIEFIEDTILEHEYLECTGSAIVVLARFMKQFPGHRTEEVKKFITKGVKYIESLQIADGSWYGNWGI  1W6K: LLNPSEVFGDIMIDYTYVECTSAVMQALKYFHKRFPEHRAAEIRETLTQGLEFCRRQQRADGSWEGSWGV  Targ: CFIYGTFFAVRGLVAAGNTYDN---CEAIRRAVRFLLDIQNGEGGWGESFLSCPNKNYIPLEGNKTDVVN  1W6K: CFTYGTWFGLEAFACMGQTYRDGTACAEVSRACDFLLSRQMADGGWGEDFESCEERRYV--QSAQSQIHN  Targ: TGQALMVLIMGGQMDRDPLPVHRAAKVLINSQMDNGDFPQQEIRGVYKMNVMLNFPTFRNSFTLWALTHY  1W6K: TCWAMMGLMAVRHP--DIEAQERGVRCLLEKQLPNGDWPQENIAGVFNKSCAISYTSYRNIFPIWALGRF  Targ: TKAIRLLL-----  1W6K: SQLYPERALAGHP |
| 270303608 | Targ: ----------------------------------------------------------------------  1W6K: -----TEGTCLRRRGGPYKTEPATDLGRWRLNCERGRQTWTYLQDERAGREQTGLEAYALGLDTKNYFKD  Targ: ------------------------------QVNYDAVTTAVKKALRLNRAIQAHDGHWPAENAGSLLYTP  1W6K: ------------------------------LPKAHTAFEGALNGMTFYVGLQAEDGHWTGDYGGPLFLLP  Targ: PLIIALYISGTIDTILTKQHKKELIRFVYNHQNEDGGWGSYIEGHSTMIGSVLSYVMLRLLGEGLAESDD  1W6K: GLLITCHVA---RIPLPAGYREEIVRYLRSVQLPDGGWGLHIEDKSTVFGTALNYVSLRILGVGPDDPD-  Targ: GNGAVERGRKWILDHGGAAGIPSWGKTYLAVLGVYEWEGCNPLPPEFWLFPSSFPFHPAKMWIYCRCTYM  1W6K: ----LVRARNILHKKGGAVAIPSWGKFWLAVLNVYSWEGLNTLFPEMWLFPDWAPAHPSTLWCHCRQVYL  Targ: PMSYLYGKRYHGPITDLVLSLRQEIYNIPYEQIKWNQQRHNCCKEDLYYPHTLVQDLVWDGLHYFSEPFL  1W6K: PMSYCYAVRLSAAEDPLVQSLRQELYVEDFASIDWLAQRNNVAPDELYTPHSWLLRVVYALLNLY-----  Targ: KRWPFNKLRKRGLKRVVELMRYGATETRFITTGNGEKALQIMSWW-AEDPNGDEFKHHLARIPDFLWIAE  1W6K: EHHHSAHLRQRAVQKLYEHIVADDRFTKSISIGPISKTINMLVRWYVDGPASTAFQEHVSRIPDYLWMGL  Targ: DGMTVQ-SFGSQLWDCILATQAIIATN--MVEEYGDSLKKAHFFIKESQIKENPRGDFLKMCRQFTKGAW  1W6K: DGMKMQGTNGSQIWDTAFAIQALLEAGGHHRPEFSSCLQKAHEFLRLSQVPDNPP-DYQKYYRQMRKGGF  Targ: TFSDQDHGCVVSDCTAEALKCLLLLSQMPQDIVGEKPEVERLYEAVNVLLYLQSRVSGGFAVWEPPVPKP  1W6K: SFSTLDCGWIVSDCTAEALKAVLLLQEKCPH-VTEHIPRERLCDAVAVLLNMRNP-DGGFATYETKRGGH  Targ: YLEMLNPSEIFADIVVEREHIECTASVIKGLMAFKCLHPGHRQKEIEDSVAKAIRYLERNQMPDGSWYGF  1W6K: LLELLNPSEVFGDIMIDYTYVECTSAVMQALKYFHKRFPEHRAAEIRETLTQGLEFCRRQQRADGSWEGS  Targ: WGICFLYGTFFTLSGFASAGRTYDN---SEAVRKGVKFFLSTQNEEGGWGESLESCPSEKFTPLKGNRTN  1W6K: WGVCFTYGTWFGLEAFACMGQTYRDGTACAEVSRACDFLLSRQMADGGWGEDFESCEERRYV--QSAQSQ  Targ: LVQTSWAMLGLMFGGQAERDPTPLHRAAKLLINAQMDNGDFPQQEITGVYCKNSMLHYAEYRNIFPLWAL  1W6K: IHNTCWAMMGLMAVRHP--DIEAQERGVRCLLEKQLPNGDWPQENIAGVFNKSCAISYTSYRNIFPIWAL  Targ: GEYRKRVWLPKHQQLKI  1W6K: GRFSQLYPERALAGHP- |
| 300807978 | Targ: ----------------------------------------------------------------------  1W6K: ------TEGTCLRRRGGPYKTEPATDLGRWRLNCERGRQTWTYLQDERAGREQTGLEAYALGLDTKNYFK  Targ: ---------------------------EEVSYEAATAALKRGVHFYSALQASDGHWPAENAGPMFFMSPL  1W6K: ---------------------------DLPKAHTAFEGALNGMTFYVGLQAEDGHWTGDYGGPLFLLPGL  Targ: VMCLYITGHLNTIFTEEHRRETLRYIYYHQNEDGGWGFHIEGQSTMFGTVLNYICMRLLGEGPEGGQDNA  1W6K: LITCHVAR---IPLPAGYREEIVRYLRSVQLPDGGWGLHIEDKSTVFGTALNYVSLRILGVGPD---DPD  Targ: VSRGRKWILDHGGATAIPSWGKTWLSIMGLCDWSGCNPMPPEFWLLPSYLPMHPAKMWCYCRMVYMPMSY  1W6K: LVRARNILHKKGGAVAIPSWGKFWLAVLNVYSWEGLNTLFPEMWLFPDWAPAHPSTLWCHCRQVYLPMSY  Targ: LYGKRFTTHITPLILQLREELHTQPYDQINWKKVRHVCCKEDTYYPHPILQDLIWDTLYLTTEPLLTRWP  1W6K: CYAVRLSAAEDPLVQSLRQELYVEDFASIDWLAQRNNVAPDELYTPHSWLLRVVYALLNLYEHHHSAH--  Targ: LNKLIRERALKKTMKHIHYEDENSRYIVIGAVEKVLCMLACW-VEDPNGDYFKKHLARVPDYFWVAEDGM  1W6K: ----LRQRAVQKLYEHIVADDRFTKSISIGPISKTINMLVRWYVDGPASTAFQEHVSRIPDYLWMGLDGM  Targ: KIQSF-GSQHWDTAFFVQALLASD--MTDEIRTTLAKAHDCIKKSQVKDNPSGDFRSMYRHISKGAWTFS  1W6K: KMQGTNGSQIWDTAFAIQALLEAGGHHRPEFSSCLQKAHEFLRLSQVPDNPP-DYQKYYRQMRKGGFSFS  Targ: DQDHGWQLSDCTAEGLKCCLLFSLMQPEVVGEAMPPERLYDSVNVLLYLQSKNGGMPGWEPAGESEWLEL  1W6K: TLDCGWIVSDCTAEALKAVLLLQEKCPH-VTEHIPRERLCDAVAVLLNMRNPDGGFATYETKRGGHLLEL  Targ: LNPTEFFENIVIEHEYVECTSSAVQALVLFKKLYPLHRRKEVERFITNGAKYLEDIQMPDGSWYGNWGVC  1W6K: LNPSEVFGDIMIDYTYVECTSAVMQALKYFHKRFPEHRAAEIRETLTQGLEFCRRQQRADGSWEGSWGVC  Targ: FTYGAWFALEGLSAAGKTYNN---CAAVRKGVDFLLNIQLEDGGWGESYQSCPDKKYVPLEDNRSNLVQT  1W6K: FTYGTWFGLEAFACMGQTYRDGTACAEVSRACDFLLSRQMADGGWGEDFESCEERRYV--QSAQSQIHNT  Targ: SWALMGLIYAGQADRDPTPLHRAAKLLINSQLEDGDFPQQEIAGVFKMNCTLHFAAYRNIFPIWALAVYR  1W6K: CWAMMGLMAVRHPDIE--AQERGVRCLLEKQLPNGDWPQENIAGVFNKSCAISYTSYRNIFPIWALGRFS  Targ: RFCNPNSEAISKPSK  1W6K: QLY-PERALAGHP-- |
| 403377906 | Targ: ----------------------------------------------------------------------  1W6K: ------TEGTCLRRRGGPYKTEPATDLGRWRLNCERGRQTWTYLQDERAGREQTGLEAYALGLDTKNYFK  Targ: ---------------------------EEISYDKVTATLRRSVHLLAALQADDGHWPAENTGPMFFIQPL  1W6K: ---------------------------DLPKAHTAFEGALNGMTFYVGLQAEDGHWTGDYGGPLFLLPGL  Targ: VICLYITGHLDRVFPKEHKKEILRYLYTQQNEDGGWGLHIEGQSIMFGTIMSYVCMRLLGEGPDGGLNGA  1W6K: LITCHVA---RIPLPAGYREEIVRYLRSVQLPDGGWGLHIEDKSTVFGTALNYVSLRILGVGPD---DPD  Targ: CTKARKWILDHGSVLASPSWGKIYLTILGVHEWEGCNPLPPEFWILPSIFPMHPAKMWCYCRLIYMPMSY  1W6K: LVRARNILHKKGGAVAIPSWGKFWLAVLNVYSWEGLNTLFPEMWLFPDWAPAHPSTLWCHCRQVYLPMSY  Targ: LYGRRFVGPITPLVLQLREELYSQSYNDIKWKSTRHLVVKEDLHYPHPWLQDLMWDGLYIFTEPLLTRWP  1W6K: CYAVRLSAAEDPLVQSLRQELYVEDFASIDWLAQRNNVAPDELYTPHSWLLRVVYALLNLYEHHHSAH--  Targ: FSKLREKALKTTINHIHYEDENSRYITIGAVEKSLCMLACW-DEDPDGVCFKKHLARIPDYIWVSEDGLK  1W6K: ---LRQRAVQKLYEHIVADDRFTKSISIGPISKTINMLVRWYVDGPASTAFQEHVSRIPDYLWMGLDGMK  Targ: MQSF-GSQLWDASLAIQALLATDLNH--DIEPILRKGHDFIKASQVKDNPSGDFKSMYRHITKGSWTFSD  1W6K: MQGTNGSQIWDTAFAIQALLEAGGHHRPEFSSCLQKAHEFLRLSQVPDNPP-DYQKYYRQMRKGGFSFST  Targ: QDHGWQTSDCTTEGLKCCLLLSKMSAEIVGEKMQPEQFYDAVNLILSLQCKNGGEAGWEPAGESNWLEFL  1W6K: LDCGWIVSDCTAEALKAVLLLQEKCPH-VTEHIPRERLCDAVAVLLNMRNPDGGFATYETKRGGHLLELL  Targ: NPSELFEDIVLEHDSVECTATGMQALVIFKKLYPKHRREEIEKFLKDACGYLEKVQMQDGSWYGEWGICF  1W6K: NPSEVFGDIMIDYTYVECTSAVMQALKYFHKRFPEHRAAEIRETLTQGLEFCRRQQRADGSWEGSWGVCF  Targ: TYGTCFALGGMEAIGKTYEN---CEAIRRAVNFLLTTQRNDGGWGESYRSSPKKKYVPLEGNRSNLVQTA  1W6K: TYGTWFGLEAFACMGQTYRDGTACAEVSRACDFLLSRQMADGGWGEDFESCEERRYV--QSAQSQIHNTC  Targ: CALMGLIRSKQEERDPTPLHRAAKLLINSQMENGDFPQEETGGVFKKNCLLHYPMYRNIYTLWALGEYRK  1W6K: WAMMGLMAVRHP--DIEAQERGVRCLLEKQLPNGDWPQENIAGVFNKSCAISYTSYRNIFPIWALGRFSQ  Targ: KVLPQPTKV--  1W6K: LYPERALAGHP |
| 350538549 | Targ: ----------------------------------------------------------------------  1W6K: ------TEGTCLRRRGGPYKTEPATDLGRWRLNCERGRQTWTYLQDERAGREQTGLEAYALGLDTKNYFK  Targ: ---------------------------EEISHEVATIALHRAVNFFSALQATDGHWPAENAGPLFFLPPL  1W6K: ---------------------------DLPKAHTAFEGALNGMTFYVGLQAEDGHWTGDYGGPLFLLPGL  Targ: VMCMYITGHL-NTVFPAEHRKEILRYIYCHQNEDGGWGLHIEGHSTMFCTALSYICMRILGEGPDGGVNN  1W6K: L----ITCHVARIPLPAGYREEIVRYLRSVQLPDGGWGLHIEDKSTVFGTALNYVSLRILGVGPD---DP  Targ: ACARARKWILDHGSVTAIPSWGKTWLSILGVFEWIGTNPMPPEFWILPSFLPVHPAKMWCYCRMVYMPMS  1W6K: DLVRARNILHKKGGAVAIPSWGKFWLAVLNVYSWEGLNTLFPEMWLFPDWAPAHPSTLWCHCRQVYLPMS  Targ: YLYGKRFVGPITPLILQLREELYDRPYDEINWKKVRHVCAKEDLYYPHPLVQDLMWDSLYICTEPLLTRW  1W6K: YCYAVRLSAAEDPLVQSLRQELYVEDFASIDWLAQRNNVAPDELYTPHSWLLRVVYALLN-----LYEHH  Targ: PFNKLRNKALEVTMKHIHYEDENSRYITIGCVEKVLCMLACW-VEDPNGDYFKKHLARIPDYLWVAEDGM  1W6K: HSAHLRQRAVQKLYEHIVADDRFTKSISIGPISKTINMLVRWYVDGPASTAFQEHVSRIPDYLWMGLDGM  Targ: KMQSF-GSQEWDTGFAIQALLASE--MNDEIADTLRKGHDFIKQSQVTNNPSGDFKGMYRHISKGSWTFS  1W6K: KMQGTNGSQIWDTAFAIQALLEAGGHHRPEFSSCLQKAHEFLRLSQVPDNPP-DYQKYYRQMRKGGFSFS  Targ: DQDHGWQVSDCTAEALKCCLLLSTMPRELVGQAMEPGRLYDSVNVVLSLQSKNGGLAAWEPAGASEYLEL  1W6K: TLDCGWIVSDCTAEALKAVLLLQEKCP-HVTEHIPRERLCDAVAVLLNMRNPDGGFATYETKRGGHLLEL  Targ: LNPTEFFADIVIEHEYVECTASSIQALVLFKKLYPGHRTKEINIFIDNAVKYLEDVQMPDGSWYGNWGVC  1W6K: LNPSEVFGDIMIDYTYVECTSAVMQALKYFHKRFPEHRAAEIRETLTQGLEFCRRQQRADGSWEGSWGVC  Targ: FTYGSWFALGGLVAAGKSYNN---SAAVRKGVEFLLRTQRSDGGWGESYRSCPDKVYRELETNDSNLVQT  1W6K: FTYGTWFGLEAFACMGQTYRDGTACAEVSRACDFLLSRQMADGGWGEDFESCEERRYVQ--SAQSQIHNT  Targ: AWALMGLIHSGQADRDPKPLHRAAKLLINSQMEDGDFPQQEITGVFMKNCMLHYAAYRNIYPLWGLAEYR  1W6K: CWAMMGLMAVRHP--DIEAQERGVRCLLEKQLPNGDWPQENIAGVFNKSCAISYTSYRNIFPIWALGRFS  Targ: KNVLLPLENN--  1W6K: QLYPERALAGHP |
| 300591999 | Targ: ----------------------------------------------------------------------  1W6K: ---------TEGTCLRRRGGPYKTEPATDLGRWRLNCERGRQTWTYLQDERAGREQTGLEAYALGLDTKN  Targ: ---------------------------------KTTTTLRRGTHHLATLQTSDGHWPAQIAGPLFFMPPL  1W6K: ------------------------YFKDLPKAHTAFEGALNGMTFYVGLQAEDGHWTGDYGGPLFLLPGL  Targ: VFCVYITGHLDSVFPPEHRKEILRYIYCHQNEDGGWGLHIEGHSTMFCTALNYICMRILGEGPDGGEDNA  1W6K: LITCHVAR---IPLPAGYREEIVRYLRSVQLPDGGWGLHIEDKSTVFGTALNYVSLRILGVGPD---DPD  Targ: CVRARNWIRQHGGVTHIPSWGKTWLSILGVFDWLGSNPMPPEFWILPSFLPMHPAKMWCYCRLVYMPMSY  1W6K: LVRARNILHKKGGAVAIPSWGKFWLAVLNVYSWEGLNTLFPEMWLFPDWAPAHPSTLWCHCRQVYLPMSY  Targ: LYGKRFVGPITPLILQLREELHTEPYEKINWTKTRHLCAKEDIYYPHPLIQDLIWDSLYIFTEPLLTRWP  1W6K: CYAVRLSAAEDPLVQSLRQELYVEDFASIDWLAQRNNVAPDELYTPHSWLLRVVYALLNLYEHHHSAH--  Targ: FNKLVRKRALEVTMKHIHYEDENSRYLTIGCVEKVLCMLACW-VEDPNGDAFKKHIARVPDYLWISEDGM  1W6K: ----LRQRAVQKLYEHIVADDRFTKSISIGPISKTINMLVRWYVDGPASTAFQEHVSRIPDYLWMGLDGM  Targ: TM-QSFGSQEWDAGFAVQALLATN--LIEEIKPALAKGHDFIKKSQVTENPSGDFKSMHRHISKGSWTFS  1W6K: KMQGTNGSQIWDTAFAIQALLEAGGHHRPEFSSCLQKAHEFLRLSQVPDNPP-DYQKYYRQMRKGGFSFS  Targ: DQDHGWQVSDCTAEGLKCCLLLSLLPPEIVGEKMEPERLFDSVNLLLSLQSKKGGLAAWEPAGAQEWLEL  1W6K: TLDCGWIVSDCTAEALKAVLLLQEKCPH-VTEHIPRERLCDAVAVLLNMRNPDGGFATYETKRGGHLLEL  Targ: LNPTEFFADIVVEHEYVECTGSAIQALVLFKKLYPGHRKKEIENFIFNAVRFLEDTQTEDGSWYGNWGVC  1W6K: LNPSEVFGDIMIDYTYVECTSAVMQALKYFHKRFPEHRAAEIRETLTQGLEFCRRQQRADGSWEGSWGVC  Targ: FTYGSWFALGGLAAAGKTYTN---CAAIRKGVKFLLTTQREDGGWGESYLSSPKKIYVPLEGNRSNVVHT  1W6K: FTYGTWFGLEAFACMGQTYRDGTACAEVSRACDFLLSRQMADGGWGEDFESCEERRYV--QSAQSQIHNT  Targ: AWALMGLIHAGQSERDPTPLHRAAKLLINSQLEQGDWPQQEITGVFMKNCMLHYPMYRDIYPLWALAEYR  1W6K: CWAMMGLMAVRHP--DIEAQERGVRCLLEKQLPNGDWPQENIAGVFNKSCAISYTSYRNIFPIWALGRFS  Targ: RRVPLP------  1W6K: QLYPERALAGHP |
| 300591997 | Targ: ----------------------------------------------------------------------  1W6K: ------TEGTCLRRRGGPYKTEPATDLGRWRLNCERGRQTWTYLQDERAGREQTGLEAYALGLDTKNYFK  Targ: ---------------------------EEISEENVAITLRRAVHHLSTLQSNDGHWPALNAGPLFYFPPL  1W6K: ---------------------------DLPKAHTAFEGALNGMTFYVGLQAEDGHWTGDYGGPLFLLPGL  Targ: VFCMYVTGHLDSIFPYEYRKEILRYIYCHQNEDGGWGLHVEGHSIMFCTVLNYICMRILGEGPNGGKEDA  1W6K: LITCHVAR---IPLPAGYREEIVRYLRSVQLPDGGWGLHIEDKSTVFGTALNYVSLRILGVGPD---DPD  Targ: CARARKWIHDHGSVTHVSSWGKIWLSVLGIFDWCASNPMPPEFWMLPSFLLKHPAKMLCYCRLVYMPMSY  1W6K: LVRARNILHKKGGAVAIPSWGKFWLAVLNVYSWEGLNTLFPEMWLFPDWAPAHPSTLWCHCRQVYLPMSY  Targ: LYGKRFVGPITPLILMLREELLTQPYEKVNWKKTRHLCAKEDLYYPHPLIQDLIWDSLYIFVEPLLTHWP  1W6K: CYAVRLSAAEDPLVQSLRQELYVEDFASIDWLAQRNNVAPDELYTPHSWLLRVVYALLNLYEHHHSAH--  Targ: FNKLLREKALQTVMKHIHYEDENSRYITIGCVEKVLCILACW-VEDPNGDAFKKHLARLPDYLWVSEDGM  1W6K: ----LRQRAVQKLYEHIVADDRFTKSISIGPISKTINMLVRWYVDGPASTAFQEHVSRIPDYLWMGLDGM  Targ: TLHSF-GSQTWDASLIIQALLATNL--IEDVGPILTKAHEFIKKSQVRDNPSGDFKSMYRHISKGSWTFS  1W6K: KMQGTNGSQIWDTAFAIQALLEAGGHHRPEFSSCLQKAHEFLRLSQVPDNPP-DYQKYYRQMRKGGFSFS  Targ: DKDHGWQVSDCTAESLKCCLLLSMLPPEIVGEKMEPEMLYDSVNILLSLQGKKGGLPAWEPSEAVEWLEL  1W6K: TLDCGWIVSDCTAEALKAVLLLQEKCPH-VTEHIPRERLCDAVAVLLNMRNPDGGFATYETKRGGHLLEL  Targ: FNPIEFLEEIVVEREYVECTSSAIQALVLFKKLYPEHRKKEVENFIANAVRFLEYKQTSDGSWYGNWGIC  1W6K: LNPSEVFGDIMIDYTYVECTSAVMQALKYFHKRFPEHRAAEIRETLTQGLEFCRRQQRADGSWEGSWGVC  Targ: FTYGSWFALNGLVAAGKTYDN---CAAIRKGVEFLLTTQREDGGWGESHLSSSKKIYVPLERSQSNIVQT  1W6K: FTYGTWFGLEAFACMGQTYRDGTACAEVSRACDFLLSRQMADGGWGEDFESCEERRYV--QSAQSQIHNT  Targ: SWAIMGLIHAGQMERDPTPLHRAVKLIINFQQEEGDWPQQELTGVFMKNCMLQYAMYRDIFPTWALAEYR  1W6K: CWAMMGLMAV--RHPDIEAQERGVRCLLEKQLPNGDWPQENIAGVFNKSCAISYTSYRNIFPIWALGRFS  Targ: RRILLASPAVAI--  1W6K: QLYPERA--LAGHP |
| 257623103 | Targ: ----------------------------------------------------------------------  1W6K: ------TEGTCLRRRGGPYKTEPATDLGRWRLNCERGRQTWTYLQDERAGREQTGLEAYALGLDTKNYFK  Targ: ----------------------------ENITVEALVTTITRAISFYSSIQAHDGHWPAESAGPLFFLQP  1W6K: ----------------------------DLPKAHTAFEGALNGMTFYVGLQAEDGHWTGDYGGPLFLLPG  Targ: LVMALYITGSL-DDVLGPEHKKEIVRYLYNHQNEDGGWGFHIEGHSTMFGSALSYVALRILGEGPQDKAM  1W6K: LL----ITCHVARIPLPAGYREEIVRYLRSVQLPDGGWGLHIEDKSTVFGTALNYVSLRILGVGPDDPDL  Targ: AKGRKWILDHGGLVAIPSWGKFWVTVLGAYEWSGCNPLPPELWLLPKFAPFHPGKMLCYCRLVYMPMSYL  1W6K: VRARNILHKKGGAVAIPSWGKFWLAVLNVYSWEGLNTLFPEMWLFPDWAPAHPSTLWCHCRQVYLPMSYC  Targ: YGKKFVGPITALIRSLREELYNEPYNQINWNTARNTVAKEDLYYPHPLIQDMLWGFLYHVGERFLNCWPF  1W6K: YAVRLSAAEDPLVQSLRQELYVEDFASIDWLAQRNNVAPDELYTPHSWLLRVVYALL-----NLYEHHHS  Targ: SMLRRKALEIAINHVHYEDENSRYLCIGSVEKVLCLIARW-VEDPNSEAYKLHLARIPDYFWLAEDGLKI  1W6K: AHLRQRAVQKLYEHIVADDRFTKSISIGPISKTINMLVRWYVDGPASTAFQEHVSRIPDYLWMGLDGMKM  Targ: Q-SFGCQMWDAAFAIQAILACN--VSEEYGPTLRKAHHFVKASQVRENPSGDFNAMYRHISKGAWTFSMH  1W6K: QGTNGSQIWDTAFAIQALLEAGGHHRPEFSSCLQKAHEFLRLSQVPDNPP-DYQKYYRQMRKGGFSFSTL  Targ: DHGWQVSDCTAEGLKAALLLSEMPSELVGGKMETERFYDAVNVILSLQSSNGGFPAWEPQKAYRWLEKFN  1W6K: DCGWIVSDCTAEALKAVLLLQEKCPH-VTEHIPRERLCDAVAVLLNMRNPDGGFATYETKRGGHLLELLN  Targ: PTEFFEDTMIEREYVECTGSAMQGLALFRKQFPQHRSKEIDRCIAKAIRYIENMQNPDGSWYGCWGICYT  1W6K: PSEVFGDIMIDYTYVECTSAVMQALKYFHKRFPEHRAAEIRETLTQGLEFCRRQQRADGSWEGSWGVCFT  Targ: YGTWFAVEGLTACGKNCHN---SLSLRKACQFLLSKQLPNAGWGESYLSSQNKVYTNLEGNRANLVQSSW  1W6K: YGTWFGLEAFACMGQTYRDGTACAEVSRACDFLLSRQMADGGWGEDFESCEERRYV--QSAQSQIHNTCW  Targ: ALLSLTHAGQAEIDPTPIHRGMKLLINSQMEDGDFPQQEITGVFMRNCTLNYSSYRNIFPIWAMGEYRRQ  1W6K: AMMGLMAVRHPDI--EAQERGVRCLLEKQLPNGDWPQENIAGVFNKSCAISYTSYRNIFPIWALGRFSQL  Targ: VLCAHSY---  1W6K: YPERALAGHP |
| 300591979 | Targ: ----------------------------------------------------------------------  1W6K: --------TEGTCLRRRGGPYKTEPATDLGRWRLNCERGRQTWTYLQDERAGREQTGLEAYALGLDTKNY  Targ: --------------------------------VITTLRRSLSFYSSIQAHDGHWPGESAGPLFFLQPFVM  1W6K: -----------------------FKDLPKAHTAFEGALNGMTFYVGLQAEDGHWTGDYGGPLFLLPGLLI  Targ: ALYITGDLNTIFSPAHQKEIIRYLYNHQNEDGGWGFHIEGHSTMFGSALSYIALRILGEGLEDGEDGAMA  1W6K: TCHVA---RIPLPAGYREEIVRYLRSVQLPDGGWGLHIEDKSTVFGTALNYVSLRILGVGPDDPD---LV  Targ: KSRKWILDHGGLVAIPSWGKFWVTVLGLYEWSGCNPLPPEFWFLPDIFPIHPGKMLCYCRLVYMPMSYLY  1W6K: RARNILHKKGGAVAIPSWGKFWLAVLNVYSWEGLNTLFPEMWLFPDWAPAHPSTLWCHCRQVYLPMSYCY  Targ: GKRFVGPITGLIQSLRQELYNEPYHQINWNKARSTVAKEDLYYPHPLIQDLLWGFLHHVAEPVLTRWPFS  1W6K: AVRLSAAEDPLVQSLRQELYVEDFASIDWLAQRNNVAPDELYTPHSWLLRVVYALLN-----LYEHHHSA  Targ: MLREKALKAAIGHVHYEDENSKYLCIGSVEKVLCLIACW-AEDPNGEAYKLHLGRIPDNYWVAEDGLKIQ  1W6K: HLRQRAVQKLYEHIVADDRFTKSISIGPISKTINMLVRWYVDGPASTAFQEHVSRIPDYLWMGLDGMKMQ  Targ: -SFGCQMWDAGFAIQAILSCNLN--EEYWPTLRKAHEFVKASQVPENPSGDFKAMYRHINKGAWTFSMQD  1W6K: GTNGSQIWDTAFAIQALLEAGGHHRPEFSSCLQKAHEFLRLSQVPDNPP-DYQKYYRQMRKGGFSFSTLD  Targ: HGWQVSDCTAEGLKVAILFSQMPPDLVGEKIEKERLYDAVNVILSLQSSNGGFPAWEPQRAYGWLEKFNP  1W6K: CGWIVSDCTAEALKAVLLLQEKCPH-VTEHIPRERLCDAVAVLLNMRNPDGGFATYETKRGGHLLELLNP  Targ: TEFFEDTLIEREYVECTSPAVHGLALFRKFYPRHRGTEIDSSIYRGIQYIEDVQEPDGSWYGHWGICYTY  1W6K: SEVFGDIMIDYTYVECTSAVMQALKYFHKRFPEHRAAEIRETLTQGLEFCRRQQRADGSWEGSWGVCFTY  Targ: GTWFAVGALAACGRNYKN---CPALRKSCEFLLSKQLPNGGWGESYLSSQNKVWTNIEGNRANLVQTAWA  1W6K: GTWFGLEAFACMGQTYRDGTACAEVSRACDFLLSRQMADGGWGEDFESCEERRYV--QSAQSQIHNTCWA  Targ: LLSLIDARQAEIDPTPIHRGVRVLINSQMEDGDFPQQEITGVFMRNCTLNYSSYRNIFPIWALGEYRRRV  1W6K: MMGLMAVRHPDI--EAQERGVRCLLEKQLPNGDWPQENIAGVFNKSCAISYTSYRNIFPIWALGRFSQLY  Targ: LFA------  1W6K: PERALAGHP |
| 30699380 | Targ: ----------------------------------------------------------------------  1W6K: -------TEGTCLRRRGGPYKTEPATDLGRWRLNCERGRQTWTYLQDERAGREQTGLEAYALGLDTKNYF  Targ: ---------------------------------ETTTNALRRGVRYFTALQASDGHWPGEITGPLFFLPP  1W6K: ---------------------------KDLPKAHTAFEGALNGMTFYVGLQAEDGHWTGDYGGPLFLLPG  Targ: LIFCLYITGHL-EEVFDAEHRKEMLRHIYCHQNEDGGWGLHIESKSVMFCTVLNYICLRMLGENPEQDAC  1W6K: LL----ITCHVARIPLPAGYREEIVRYLRSVQLPDGGWGLHIEDKSTVFGTALNYVSLRILGVGPDDPDL  Targ: KRARQWILDRGGVIFIPSWGKFWLSILGVYDWSGTNPTPPELLMLPSFLPIHPGKILCYSRMVSIPMSYL  1W6K: VRARNILHKKGGAVAIPSWGKFWLAVLNVYSWEGLNTLFPEMWLFPDWAPAHPSTLWCHCRQVYLPMSYC  Targ: YGKRFVGPITPLILLLREELYLEPYEEINWKKSRRLYAKEDMYYAHPLVQDLLSDTLQNFVEPLLTRWPL  1W6K: YAVRLSAAEDPLVQSLRQELYVEDFASIDWLAQRNNVAPDELYTPHSWLLRVVYALLNLYEHHH------  Targ: NKLVREKALQLTMKHIHYEDENSHYITIGCVEKVLCMLACW-VENPNGDYFKKHLARIPDYMWVAEDGMK  1W6K: SAHLRQRAVQKLYEHIVADDRFTKSISIGPISKTINMLVRWYVDGPASTAFQEHVSRIPDYLWMGLDGMK  Targ: MQ-SFGCQLWDTGFAIQALLASN--LPDETDDALKRGHNYIKASQVRENPSGDFRSMYRHISKGAWTFSD  1W6K: MQGTNGSQIWDTAFAIQALLEAGGHHRPEFSSCLQKAHEFLRLSQVPDNPP-DYQKYYRQMRKGGFSFST  Targ: RDHGWQVSDCTAEALKCCLLLSMMSADIVGQKIDDEQLYDSVNLLLSLQSGNGGVNAWEPSRAYKWLELL  1W6K: LDCGWIVSDCTAEALKAVLLLQEKCPH-VTEHIPRERLCDAVAVLLNMRNPDGGFATYETKRGGHLLELL  Targ: NPTEFMANTMVEREFVECTSSVIQALDLFRKLYPDHRKKEINRSIEKAVQFIQDNQTPDGSWYGNWGVCF  1W6K: NPSEVFGDIMIDYTYVECTSAVMQALKYFHKRFPEHRAAEIRETLTQGLEFCRRQQRADGSWEGSWGVCF  Targ: IYATWFALGGLAAAGETYND---CLAMRNGVHFLLTTQRDDGGWGESYLSCSEQRYIPSEGERSNLVQTS  1W6K: TYGTWFGLEAFACMGQTYRDGTACAEVSRACDFLLSRQMADGGWGEDFESCEERRYV--QSAQSQIHNTC  Targ: WAMMALIHTGQAERDLIPLHRAAKLIINSQLENGDFPQQEIVGAFMNTCMLHYATYRNTFPLWALAEYRK  1W6K: WAMMGLMAVRHPDIE--AQERGVRCLLEKQLPNGDWPQENIAGVFNKSCAISYTSYRNIFPIWALGRFSQ  Targ: VVFIVN-----  1W6K: LYPERALAGHP |
| 353558864 | Targ: ----------------------------------------------------------------------  1W6K: ------TEGTCLRRRGGPYKTEPATDLGRWRLNCERGRQTWTYLQDERAGREQTGLEAYALGLDTKNYFK  Targ: ---------------------------EEITREKATTALRRAVQFFSALQASDGHWPAENAGPLFFLPPL  1W6K: ---------------------------DLPKAHTAFEGALNGMTFYVGLQAEDGHWTGDYGGPLFLLPGL  Targ: VMCMCITGHLDT-VFPAEHRKEILRYIYYHQNEDGGWGLHIEGHSTMFCTALNYICMRILGEGPNGGQDD  1W6K: L----ITCHVARIPLPAGYREEIVRYLRSVQLPDGGWGLHIEDKSTVFGTALNYVSLRILGVGPD---DP  Targ: ACTRARKWIHDHGSVTNIPSWGKTWLSILGVYDWSGCNPMPPEFWMLPSFLPMHPAKMWCYCRMVYMPMS  1W6K: DLVRARNILHKKGGAVAIPSWGKFWLAVLNVYSWEGLNTLFPEMWLFPDWAPAHPSTLWCHCRQVYLPMS  Targ: YLYGKRFVGLITPLIQQLREELFTQPYDQINWKKNCHQCAPEDLYYPHPFIQDLIWDCLYISMEPLLTRW  1W6K: YCYAVRLSAAEDPLVQSLRQELYVEDFASIDWLAQRNNVAPDELYTPHSWLLRVVYALLNLYE------H  Targ: PLNMIIRKKALELTMKHIHYEDGSSRYITIGCVEKVLCMLACWV-EDPNGDYFKKHLARIPDYIWVAEDG  1W6K: HHSAHLRQRAVQKLYEHIVADDRFTKSISIGPISKTINMLVRWYVDGPASTAFQEHVSRIPDYLWMGLDG  Targ: MKMQSF-GSQQWDTGFAIQALLATN--LTDEIGGVLRRGHDFIKKSQVQDNPSGDFKSMYRHISKGSWTF  1W6K: MKMQGTNGSQIWDTAFAIQALLEAGGHHRPEFSSCLQKAHEFLRLSQVPDNPP-DYQKYYRQMRKGGFSF  Targ: SDQDHGWQVSDCTAEGLKCCLLFSMMPPEIVGEHMEPERLYDSVNVLLSLQSKNGGLSAWEPAGAQDWLE  1W6K: STLDCGWIVSDCTAEALKAVLLLQEKCPH-VTEHIPRERLCDAVAVLLNMRNPDGGFATYETKRGGHLLE  Targ: LLNPTEFFADIVIEHEYVERTSSAIHALVLFKKLYPGHRKKEIEDFIAKSVRFLESIQTSDGTWYGNWGV  1W6K: LLNPSEVFGDIMIDYTYVECTSAVMQALKYFHKRFPEHRAAEIRETLTQGLEFCRRQQRADGSWEGSWGV  Targ: CFTYGTWFALGGLAAAGKTYNS---CLAMRKAVDFLLRIQKDDGGWGESYLSCPEKKYVPLEANHSNLVH  1W6K: CFTYGTWFGLEAFACMGQTYRDGTACAEVSRACDFLLSRQMADGGWGEDFESCEERRYV--QSAQSQIHN  Targ: TAWAMMALVHAGQMDRDPTPLHRAAKLMINSQLEDGDFPQQEITGVFNRNCMLHYAAYRNIYPLWALAEY  1W6K: TCWAMMGLMAVRHPDIE--AQERGVRCLLEKQLPNGDWPQENIAGVFNKSCAISYTSYRNIFPIWALGRF  Targ: CRRVPLPS-----  1W6K: SQLYPERALAGHP |
| 300807974 | Targ: ----------------------------------------------------------------------  1W6K: ----------TEGTCLRRRGGPYKTEPATDLGRWRLNCERGRQTWTYLQDERAGREQTGLEAYALGLDTK  Targ: ----------------------------------EEITYETATTALKRAVHFYSALQASDGHWPAENSGP  1W6K: ------------------------------NYFKDLPKAHTAFEGALNGMTFYVGLQAEDGHWTGDYGGP  Targ: LFFLPPLVMCLYITGHLNTVFPAEHQREILRYIYYHQNEDGGWGLHIEGHSTMFCTALSYICMRILGEGP  1W6K: LFLLPGLLIT---CHVARIPLPAGYREEIVRYLRSVQLPDGGWGLHIEDKSTVFGTALNYVSLRILGVGP  Targ: DGGLDNAVARGRKWILDHGTVTAMPSWGKTWLSIMGLFDWSGSNPMPPEFWLLPSFLPMYPAKMWCYCRM  1W6K: D---DPDLVRARNILHKKGGAVAIPSWGKFWLAVLNVYSWEGLNTLFPEMWLFPDWAPAHPSTLWCHCRQ  Targ: VYMPMSYLYGKRFVGPITPLILQLREELYDQPYEQVNWKQVRHECAKEDIYYPHPKIQDLLWDTLYIAIE  1W6K: VYLPMSYCYAVRLSAAEDPLVQSLRQELYVEDFASIDWLAQRNNVAPDELYTPHSWLLRVVYALLN----  Targ: PLLTRWPFNKLVRERALQRTMKHIHYEDENSRYITIGCVEKVLCMLACW-VEDPNGDYFKKHLARVPDYI  1W6K: --LYEHHHSAHLRQRAVQKLYEHIVADDRFTKSISIGPISKTINMLVRWYVDGPASTAFQEHVSRIPDYL  Targ: WVAEDGMKMQ-SFGSQQWDTGFAIQALLASN--MSDEIGETLAKGHDFVKKSQVKDNPSGDFKSMHRHIS  1W6K: WMGLDGMKMQGTNGSQIWDTAFAIQALLEAGGHHRPEFSSCLQKAHEFLRLSQVPDNPP-DYQKYYRQMR  Targ: KGSWTFSDQDHGWQVSDCTAEGLKCCLLFSLMPPELVGEKMEPERLYDSVNILLSLQSKNGGLAAWEPAG  1W6K: KGGFSFSTLDCGWIVSDCTAEALKAVLLLQEKCPH-VTEHIPRERLCDAVAVLLNMRNPDGGFATYETKR  Targ: APEWLELLNPTEFFADIVIEHEYVECTASAIQALVLFKKLYPGHRKKDIETFIKGAAQYIEDRQMPDGSW  1W6K: GGHLLELLNPSEVFGDIMIDYTYVECTSAVMQALKYFHKRFPEHRAAEIRETLTQGLEFCRRQQRADGSW  Targ: YGSWGVCFTYGTWFALGGLAAAGKNYDN---CAAIRKGTEFLLNTQCENGGWGESYRSCPEKRYVPLEEN  1W6K: EGSWGVCFTYGTWFGLEAFACMGQTYRDGTACAEVSRACDFLLSRQMADGGWGEDFESCEERRYV--QSA  Targ: KSNLVHTAWALMGLIHSRQAERDITPLHRAAKLLINSQLENGDFPQQEITGVFMKNCMQHYAAYRNIYPL  1W6K: QSQIHNTCWAMMGLMAV--RHPDIEAQERGVRCLLEKQLPNGDWPQENIAGVFNKSCAISYTSYRNIFPI  Targ: WGIAEYRKQIPLPLR----  1W6K: WALGRFSQLYPERALAGHP |
| 300807976 | Targ: ----------------------------------------------------------------------  1W6K: -------TEGTCLRRRGGPYKTEPATDLGRWRLNCERGRQTWTYLQDERAGREQTGLEAYALGLDTKNYF  Targ: --------------------------GEEVSYEAVTAALRRGVHLYSALQASDGHWPAENAGPMFFMPPM  1W6K: --------------------------KDLPKAHTAFEGALNGMTFYVGLQAEDGHWTGDYGGPLFLLPGL  Targ: VMCLYITGHLNAIFTEEHRSETLRYIYYHQNEDGGWGFHIEGHSTMFGTVLNYICMRLLGEGPEGGQDNA  1W6K: LITCHVAR---IPLPAGYREEIVRYLRSVQLPDGGWGLHIEDKSTVFGTALNYVSLRILGVGPD---DPD  Targ: VSRGRKWILDHGGATSIPSWGKTWLSIMGLCDWSGCNPMPPEFWLLPSYLPMHPGKMWCYCRMVYMPMSY  1W6K: LVRARNILHKKGGAVAIPSWGKFWLAVLNVYSWEGLNTLFPEMWLFPDWAPAHPSTLWCHCRQVYLPMSY  Targ: LYGKRFTARITPLILQLREEIHIQPYDQIDWKKVRHVCCKEDMYYPHPLLQDLLWDTLYLTTEPLLTRWP  1W6K: CYAVRLSAAEDPLVQSLRQELYVEDFASIDWLAQRNNVAPDELYTPHSWLLRVVYALLN------LYEHH  Targ: LNKLIRQRALQKTMKHIHYEDENSRYITIGTVEKVLCMLACW-VEDPNGDYFKKHLARVPDYFWVAEDGM  1W6K: HSAHLRQRAVQKLYEHIVADDRFTKSISIGPISKTINMLVRWYVDGPASTAFQEHVSRIPDYLWMGLDGM  Targ: KIQSF-GSQHWDTVFSAQALLASD--MADEIGTTLAKAHYCIKESQVKDNPSGDFRSMYRHISKGSWTFS  1W6K: KMQGTNGSQIWDTAFAIQALLEAGGHHRPEFSSCLQKAHEFLRLSQVPDNPP-DYQKYYRQMRKGGFSFS  Targ: DQDHGWQLSDCTAEGLKCCLLFSLMQPEVVGEAMPPERLFDSVNILLYLQSKNGGMPGWEPAGASEWLEL  1W6K: TLDCGWIVSDCTAEALKAVLLLQEKCPH-VTEHIPRERLCDAVAVLLNMRNPDGGFATYETKRGGHLLEL  Targ: LNPTEFFENIVIEHEYVECTSSAVQALVLFKKLHPGHRRKEVERFITNGAKYIEDIQMPDGAWYGNWGVC  1W6K: LNPSEVFGDIMIDYTYVECTSAVMQALKYFHKRFPEHRAAEIRETLTQGLEFCRRQQRADGSWEGSWGVC  Targ: FTYGAWFALGGLAAAGKTYNN---CAAVRKGVDFLLRIQLEDGGWGESYQSCPDKKYVPLEDNRSNLVHT  1W6K: FTYGTWFGLEAFACMGQTYRDGTACAEVSRACDFLLSRQMADGGWGEDFESCEERRYV--QSAQSQIHNT  Targ: SWALMGLLCSGQADRDPNPLHRAAKLLINSQLEDGDFPQQEITGVFKMNCMLHFAAYRSIFPVWALAEYK  1W6K: CWAMMGLMAVRHP--DIEAQERGVRCLLEKQLPNGDWPQENIAGVFNKSCAISYTSYRNIFPIWALGRFS  Targ: RFCNLSSEAISKPSK  1W6K: QLYPERALAGH-P-- |
| 15233798 | Targ: ----------------------------------------------------------------------  1W6K: ----------TEGTCLRRRGGPYKTEPATDLGRWRLNCERGRQTWTYLQDERAGREQTGLEAYALGLDTK  Targ: ---------------------------AEKITYEDAKTALRRGLLYFTALQADDGHWPAENAGSIFFNAP  1W6K: ------------------------NYFKDLPKAHTAFEGALNGMTFYVGLQAEDGHWTGDYGGPLFLLPG  Targ: FVICLYITGHLEKIFTHEHRVELLRYMYNHQNEDGGWGLHVESPSNMFCSVINYICLRILGVEAGHDDKG  1W6K: LLITCHVA---RIPLPAGYREEIVRYLRSVQLPDGGWGLHIEDKSTVFGTALNYVSLRILGVGPD----D  Targ: SACARARKWILDHGGATYSPLIGKAWLSVLGVYDWSGCKPIPPEFWFLPSFFPVNGGTLWIYLRDIFMGL  1W6K: PDLVRARNILHKKGGAVAIPSWGKFWLAVLNVYSWEGLNTLFPEMWLFPDWAPAHPSTLWCHCRQVYLPM  Targ: SYLYGKNFVATSTPLILQLREEIYPEPYTNISWRQARNRCAKEDLYYPQSFLQDLFWKGVHVFSENILNR  1W6K: SYCYAVRLSAAEDPLVQSLRQELYVEDFASIDWLAQRNNVAPDELYTPHSWLLRVVYALLNLYEHH----  Targ: WPFNNLIRQRALRTTMELVHYHDEATRYITGGSVPKVIAVFHMLACWVEDPESDYFKKHLARVPDFIWIG  1W6K: --HSAHLRQRAVQKLYEHIVADDRFTKSISIGPISKTINML--VRWYVDGPASTAFQEHVSRIPDYLWMG  Targ: EDGLKIQSF-GSQVWDTALSLHVFIDGFDDDVDEEIRSTLLKGYDYLEKSQVTENPPGDYMKMFRHMAKG  1W6K: LDGMKMQGTNGSQIWDTAFAIQALLEAGGHHR-PEFSSCLQKAHEFLRLSQVPDNPP-DYQKYYRQMRKG  Targ: GWTFSDQDQGWPVSDCTAESLECCLFFESMSSEFIGKKMDVEKLYDAVDFLLYLQSDNGGITAWQPADG-  1W6K: GFSFSTLDCGWIVSDCTAEALKAVLLLQEKCPH-VTEHIPRERLCDAVAVLLNMRNPDGGFATYETKRGG  Targ: ------KLVEFIEDAVVEHEYVECTGSAIVALAQFNKQFPGYKKEEVERFITKGVKYIEDLQMVDGSWYG  1W6K: HLLELLNPSEVFGDIMIDYTYVECTSAVMQALKYFHKRFPEHRAAEIRETLTQGLEFCRRQQRADGSWEG  Targ: NWGVCFIYGTFFAVRGLVAAGKCYNN---CEAIRRAVRFILDTQNTEGGWGESYLSCPRKKYIPLIGNKT  1W6K: SWGVCFTYGTWFGLEAFACMGQTYRDGTACAEVSRACDFLLSRQMADGGWGEDFESCEERRYVQ--SAQS  Targ: NVVNTGQALMVLIMGNQMKRDPLPVHRAAKVLINSQMDNGDFPQQEIMGVFKMNVMLHFPTYRNMFTLWA  1W6K: QIHNTCWAMMGLMAVRHP--DIEAQERGVRCLLEKQLPNGDWPQENIAGVFNKSCAISYTSYRNIFPIWA  Targ: LTHYTKALRGL------  1W6K: LGRFSQLYPERALAGHP |
| 15218390 | Targ: ----------------------------------------------------------------------  1W6K: ------TEGTCLRRRGGPYKTEPATDLGRWRLNCERGRQTWTYLQDERAGREQTGLEAYALGLDTKNYFK  Targ: ----------------------------ENITYKDAKTALRRGILYFKALQAEDGHWPAENSGCLFFEAP  1W6K: ----------------------------DLPKAHTAFEGALNGMTFYVGLQAEDGHWTGDYGGPLFLLPG  Targ: FVICLYITGHLEKILTLEHRKELLRYMYNHQNEDGGWGIHVEGQSAMFCTVINYICLRILGVEADLDDIK  1W6K: LLITCHVA---RIPLPAGYREEIVRYLRSVQLPDGGWGLHIEDKSTVFGTALNYVSLRILGVGPDDPD--  Targ: GSGCARARKWILDHGGATYTPLIGKAWLSILGVYDWSGCKPIPPEVWMLPTFSPFNGGTLWIYFRDIFMG  1W6K: ---LVRARNILHKKGGAVAIPSWGKFWLAVLNVYSWEGLNTLFPEMWLFPDWAPAHPSTLWCHCRQVYLP  Targ: VSYLYGKKFVATPTPLILQLREELYPQPYDKILWSQARNQCAKEDLYYPQSFLQEMFWKCVHILSENILN  1W6K: MSYCYAVRLSAAEDPLVQSLRQELYVEDFASIDWLAQRNNVAPDELYTPHSWLLRVVYALLNLYEHHH--  Targ: RWPCNKLIRQKALRTTMELLHYQDEASRYFTGGCVPKPFHMLACW-VEDPDGDYFKKHLARVPDYIWIGE  1W6K: ----SAHLRQRAVQKLYEHIVADDRFTKSISIGPISKTINMLVRWYVDGPASTAFQEHVSRIPDYLWMGL  Targ: DGLKIQ-SFGSQLWDTAFSLQVMLAYQDVDDDDDEIRSTLIKGYSFLNKSQLTQNPPGDHRKMLKDIAKG  1W6K: DGMKMQGTNGSQIWDTAFAIQALLEAGG--HHRPEFSSCLQKAHEFLRLSQVPDNPP-DYQKYYRQMRKG  Targ: GWTFSDQDQGWPVSDCTAESLECCLVFGSMPSELIGEKMDVERLYDAVNLLLYFQSKNGGITVWEAARGR  1W6K: GFSFSTLDCGWIVSDCTAEALKAVLLLQEKCPH-VTEHIPRERLCDAVAVLLNMRNPDGGFATYETKRGG  Targ: TWLEWLSPVEFMEDTIVEHEYVECTGSAIVALARFLKEFPEHRREEVEKFIKNAVKYIESFQMPDGSWYG  1W6K: HLLELLNPSEVFGDIMIDYTYVECTSAVMQALKYFHKRFPEHRAAEIRETLTQGLEFCRRQQRADGSWEG  Targ: NWGVCFMYGTFFAVRGLVAAGKTYQN---CEPIRKAVQFILETQNVEGGWGESYLSCPNKKYTLLEGNRT  1W6K: SWGVCFTYGTWFGLEAFACMGQTYRDGTACAEVSRACDFLLSRQMADGGWGEDFESCEERRYV--QSAQS  Targ: NVVNTGQALMVLIMGGQMERDPLPVHRAAKVLINSQLDNGDFPQEEIMGVFKMNVMVHYATYRNIFTLWA  1W6K: QIHNTCWAMMGLMAV--RHPDIEAQERGVRCLLEKQLPNGDWPQENIAGVFNKSCAISYTSYRNIFPIWA  Targ: LTYYTKALRVPLC----  1W6K: LGRFSQLYPERALAGHP |
