# Supplementary material for: Predicting the Functions and Specificity of Triterpenoid Synthases: A Mechanism-Based Multi-intermediate Docking Approach
Source: PLoS Comput Biol. 2014 Oct 9;10(10):e1003874. doi: 10.1371/journal.pcbi.1003874 (PMC4191879; doi:10.1371/journal.pcbi.1003874)
Supplement: Table S4 — Quality assessment of homology models by using discrete optimized protein energy (DOPE) score. (DOCX) [file pcbi.1003874.s008.docx]

Table S4. Quality assessment of homology models by using discrete optimized protein energy (DOPE) score. All the models have Z score smaller than -0.9, indicating that the current automated workflow generates reasonable models for docking.

| Model Name | DOPE Z score |
| --- | --- |
| 108864084-1W6K.pdb | -1.15087 |
| 114053041-1W6K.pdb | -1.83592 |
| 118901781-1W6K.pdb | -1.18109 |
| 119499584-1W6K.pdb | -1.15534 |
| 13591981-1W6K.pdb | -1.76685 |
| 15076955-1W6K.pdb | -1.42354 |
| 15218390-1W6K.pdb | -1.05705 |
| 15233798-1W6K.pdb | -0.97281 |
| 16519641-1SQC.pdb | -1.23680 |
| 167295241-1W6K.pdb | -1.25457 |
| 167296361-1SQC.pdb | -1.06527 |
| 2113823-1SQC.pdb | -1.07030 |
| 211926830-1W6K.pdb | -0.97763 |
| 224177558-1W6K.pdb | -1.76299 |
| 240256372-1W6K.pdb | -1.10709 |
| 257623103-1W6K.pdb | -1.07385 |
| 260037884-1W6K.pdb | -0.95784 |
| 26346907-1W6K.pdb | -1.78101 |
| 270303608-1W6K.pdb | -1.07137 |
| 300591899-1W6K.pdb | -1.35429 |
| 300591911-1W6K.pdb | -1.41542 |
| 300591913-1W6K.pdb | -1.36182 |
| 300591977-1W6K.pdb | -1.00668 |
| 300591979-1W6K.pdb | -1.19085 |
| 300591981-1W6K.pdb | -1.33676 |
| 300591983-1W6K.pdb | -1.45150 |
| 300591987-1W6K.pdb | -1.02189 |
| 300591997-1W6K.pdb | -1.16530 |
| 300591999-1W6K.pdb | -1.21760 |
| 300592003-1W6K.pdb | -1.14591 |
| 300592007-1W6K.pdb | -1.38254 |
| 300592019-1W6K.pdb | -1.40383 |
| 300807974-1W6K.pdb | -1.05173 |
| 300807976-1W6K.pdb | -1.01763 |
| 300807978-1W6K.pdb | -1.24478 |
| 300807980-1W6K.pdb | -1.09985 |
| 300807982-1W6K.pdb | -1.27795 |
| 30699377-1W6K.pdb | -1.07676 |
| 30699380-1W6K.pdb | -1.11778 |
| 350538403-1W6K.pdb | -1.17449 |
| 350538549-1W6K.pdb | -1.09452 |
| 353558692-1W6K.pdb | -1.07847 |
| 353558864-1W6K.pdb | -0.95322 |
| 353678016-1W6K.pdb | -1.05261 |
| 353678133-1W6K.pdb | -1.06251 |
| 357580429-1SQC.pdb | -1.21187 |
| 403377906-1W6K.pdb | -0.98865 |
| 452446-1W6K.pdb | -1.45359 |
| 6090879-1W6K.pdb | -1.41701 |
| 63054562-1W6K.pdb | -1.33763 |
| 6466213-1SQC.pdb | -1.07924 |
| 66825783-1W6K.pdb | -1.64660 |
| 68466833-1W6K.pdb | -1.19628 |
| 70993016-1W6K.pdb | -1.28092 |
| 82468803-1W6K.pdb | -1.07294 |
| 82468805-1W6K.pdb | -1.37038 |
