# Supplementary material for: Predicting the Functions and Specificity of Triterpenoid Synthases: A Mechanism-Based Multi-intermediate Docking Approach
Source: PLoS Comput Biol. 2014 Oct 9;10(10):e1003874. doi: 10.1371/journal.pcbi.1003874 (PMC4191879; doi:10.1371/journal.pcbi.1003874)
Supplement: Table S5 — RMSD for the active site residues of crystal structures and those in the IFD. (DOCX) [file pcbi.1003874.s009.docx]

Table S5. RMSD for the active site residues of crystal structures and those in the IFD.

Table S5a. RMSD for 1SQC. Three residues show large RMSD variance (>0.7 Å, see red entries), all of which have aromatic rings.

|  | A-I1 | B-I1 | C-I1 | D-I1 | A-I2 | B-I2 | C-I2 | D-I2 |
| --- | --- | --- | --- | --- | --- | --- | --- | --- |
| LEU 36 | 0.214505 | - | 0.237181 | - | 0.213009 | - | 0.187298 | - |
| MET 42 | 0.342088 | - | 0.320396 | - | 0.376369 | - | 0.339859 | - |
| TRP 169 | 0.298217 | - | 0.333647 | - | 0.299219 | - | 0.336134 | - |
| ILE 261 | 0.143164 | - | 0.424320 | - | 0.137465 | - | 0.472064 | - |
| GLN 262 | 0.502244 | - | 0.569003 | - | 0.516688 | - | 0.604605 | - |
| PRO 263 | 0.256694 | - | 0.221104 | - | 0.176004 | - | 0.214103 | - |
| ALA 306 | 0.244515 | - | 0.224134 | - | 0.306836 | - | 0.316490 | - |
| SER 307 | 0.319561 | - | 0.271393 | - | 0.297940 | - | 0.406644 | - |
| TRP 312 | 0.360543 | - | 0.303391 | - | 0.336005 | - | 0.377340 | - |
| PHE 365 | 0.273725 | - | 0.364347 | - | 0.280450 | - | 0.270564 | - |
| GLN 366 | 0.367594 | - | 0.390349 | - | 0.371579 | - | 0.362172 | - |
| ASP 374 | 0.363257 | - | 0.239179 | - | 0.374195 | - | 0.280566 | - |
| ASP 376 | 0.406802 | - | 0.690577 | - | 0.133477 | - | 0.629060 | - |
| ASP 377 | 0.446177 | - | 0.464752 | - | 0.527370 | - | 0.501001 | - |
| ALA 419 | 0.178439 | - | 0.165067 | - | 0.209941 | - | 0.170516 | - |
| TYR 420 | 0.460251 | - | 0.254661 | - | 0.483718 | - | 0.275483 | - |
| PHE 437 | 0.757997 | - | 0.433315 | - | 0.523241 | - | 0.361048 | - |
| TRP 489 | 0.292224 | - | 0.493425 | - | 0.230839 | - | 0.401560 | - |
| TYR 495 | 0.190593 | - | 0.200924 | - | 0.186579 | - | 0.187575 | - |
| GLY 600 | 0.147431 | - | 0.118729 | - | 0.546160 | - | 0.169947 | - |
| PHE 601 | 0.801468 | - | 0.587161 | - | 0.926953 | - | 0.447990 | - |
| PHE 605 | 0.269801 | - | 0.202829 | - | 0.258144 | - | 0.239409 | - |
| LEU 607 | 0.353717 | - | 0.402518 | - | 0.365473 | - | 0.422802 | - |
| TYR 609 | 0.290899 | - | 0.702012 | - | 0.309849 | - | 0.368370 | - |
| TYR 612 | 0.239009 | - | 0.188555 | - | 0.235230 | - | 0.247351 | - |

Table S5b. RMSD for 1W6K. Three residues show large RMSD variance (>0.7 Å, see red entries), two of which have aromatic rings, and the other is leucine.

|  | A-I1 | B-I1 | C-I1 | D-I1 | A-I2 | B-I2 | C-I2 | D-I2 |
| --- | --- | --- | --- | --- | --- | --- | --- | --- |
| TYR 98 | 0.276885 | 0.340509 | 0.281010 | 0.205587 | 0.255938 | 0.333547 | 0.416043 | 0.537770 |
| PRO 101 | 0.182105 | 0.171729 | 0.177735 | 0.162258 | 0.192653 | 0.176497 | 0.183519 | 0.165039 |
| PHE 103 | 0.838656 | 0.210687 | 0.491044 | 0.314453 | 0.656677 | 0.386625 | 0.367527 | 0.355683 |
| TRP 192 | 0.442532 | 0.281096 | 0.332638 | 0.812534 | 0.388753 | 0.233790 | 0.337227 | 0.609005 |
| TRP 230 | 0.117889 | 0.115107 | 0.149308 | 0.117826 | 0.184338 | 0.160594 | 0.124433 | 0.109175 |
| HID 232 | 0.392206 | 0.364639 | 0.309381 | 0.299397 | 0.343226 | 0.401096 | 0.361932 | 0.376153 |
| CYS 233 | 0.243189 | 0.188954 | 0.209195 | 0.273841 | 0.235989 | 0.228580 | 0.217248 | 0.251353 |
| VAL 236 | 0.190899 | 0.209448 | 0.207026 | 0.323508 | 0.197791 | 0.181712 | 0.212761 | 0.170494 |
| TYR 237 | 0.602640 | 0.537782 | 0.567473 | 0.709210 | 0.581989 | 0.570737 | 0.570461 | 0.641872 |
| ILE 335 | 0.156942 | 0.153892 | 0.141632 | 0.151147 | 0.128204 | 0.116632 | 0.127487 | 0.171325 |
| GLY 336 | 0.196944 | 0.160005 | 0.161066 | 0.200807 | 0.165980 | 0.146981 | 0.159156 | 0.203118 |
| PRO 337 | 0.158799 | 0.188768 | 0.159625 | 0.168195 | 0.144074 | 0.145505 | 0.148538 | 0.182930 |
| ILE 338 | 0.145074 | 0.554971 | 0.238060 | 0.923883 | 0.165120 | 1.012261 | 0.214348 | 0.760926 |
| GLY 380 | 0.118603 | 0.126112 | 0.114694 | 0.152494 | 0.162219 | 0.154417 | 0.203663 | 0.305726 |
| THR 381 | 0.263714 | 0.277869 | 0.271028 | 0.252342 | 0.219989 | 0.243124 | 0.235554 | 0.255705 |
| TRP 387 | 0.627420 | 0.508970 | 0.600612 | 0.569903 | 0.586633 | 0.379382 | 0.533567 | 0.341891 |
| PHE 444 | 0.199965 | 0.218996 | 0.207612 | 0.217194 | 0.212058 | 0.189387 | 0.192030 | 0.135239 |
| VAL 453 | 0.172297 | 0.154260 | 0.151161 | 0.165746 | 0.176902 | 0.089273 | 0.157140 | 0.074378 |
| ASP 455 | 0.319543 | 0.356889 | 0.440914 | 0.290299 | 0.245466 | 0.357496 | 0.434544 | 0.272180 |
| CYS 456 | 0.198065 | 0.183172 | 0.185292 | 0.187514 | 0.193788 | 0.193086 | 0.205500 | 0.158854 |
| THR 502 | 0.180194 | 0.169618 | 0.174808 | 0.162951 | 0.165388 | 0.174833 | 0.178794 | 0.228407 |
| TYR 503 | 0.271018 | 0.175259 | 0.167548 | 0.207449 | 0.178804 | 0.205393 | 0.142650 | 0.217053 |
| SER 518 | 0.106830 | 0.107562 | 0.107327 | 0.112401 | 0.108565 | 0.110146 | 0.109624 | 0.110044 |
| PHE 521 | 0.465927 | 0.286136 | 0.303103 | 1.237279 | 0.440578 | 0.256367 | 0.262763 | 1.150318 |
| ILE 524 | 0.213837 | 0.164651 | 0.205127 | 0.232231 | 0.212392 | 0.187483 | 0.200549 | 0.232970 |
| GLU 532 | 0.148807 | 0.181418 | 0.176752 | 0.162483 | 0.171539 | 0.180141 | 0.176632 | 0.168501 |
| CYS 533 | 0.167069 | 0.188102 | 0.186503 | 0.192465 | 0.182946 | 0.104300 | 0.205188 | 0.161151 |
| TRP 581 | 0.361784 | 0.419798 | 0.501968 | 0.349982 | 0.361861 | 0.389863 | 0.538311 | 0.306076 |
| TYR 587 | 0.113378 | 0.124508 | 0.098048 | 0.096070 | 0.120697 | 0.126061 | 0.114044 | 0.124501 |
| VAL 695 | 0.174406 | 0.148042 | 0.151423 | 0.182788 | 0.157787 | 0.130010 | 0.139951 | 0.167530 |
| PHE 696 | 0.203167 | 0.345064 | 0.295227 | 0.288561 | 0.233611 | 0.327202 | 0.343626 | 0.280907 |
| ASN 697 | 0.411920 | 0.345477 | 0.387314 | 0.671595 | 0.335919 | 0.277125 | 0.337942 | 0.604650 |
| ILE 702 | 0.076277 | 0.109113 | 0.085320 | 0.058127 | 0.078072 | 0.114432 | 0.103626 | 0.113097 |
| TYR 704 | 0.873730 | 0.575414 | 0.846459 | 0.827567 | 0.531561 | 0.222408 | 0.829295 | 0.736039 |
